# Modulating Receptor Activity, Immune Response, and Kinetic Solubility: The Impact of Linker Chemistry in Conjugated NOD2/TLR4 Agonists

**DOI:** 10.1021/acsomega.5c05358

**Published:** 2025-08-22

**Authors:** Emiliano Paradiso, Špela Janež, Žiga Jakopin

**Affiliations:** Faculty of Pharmacy, Department of Pharmaceutical Chemistry, University of Ljubljana, Aškerčeva 7, SI-1000 Ljubljana, Slovenia

## Abstract

Novel immunopotentiators are essential for advancing our understanding of immune receptor crosstalk and for addressing infectious diseases. Previous studies have suggested that coactivation of nucleotide-binding oligomerization domain-containing protein 2 (NOD2) and Toll-like receptor 4 (TLR4) can synergistically enhance the immune response. To investigate this synergy, we synthesized and evaluated a series of conjugated NOD2/TLR4 dual agonists comprising our in-house NOD2 agonist and two structurally distinct TLR4 agonists connected via flexible or rigid linkers. Our findings indicate that dual agonist activity toward both NOD2 and TLR4 is diminished upon conjugation. We also show that the linker chemistry significantly influences the kinetic solubility of these conjugates. Furthermore, the conjugates elicit distinct immunomodulatory effects in human primary peripheral blood mononuclear cells, characterized by a Th2-polarized cytokine response. These results provide insights into the structure–activity relationship of conjugated NOD2/TLR4 agonists and offer preliminary guidelines for tuning their solubility profiles.

## Graphical abstract

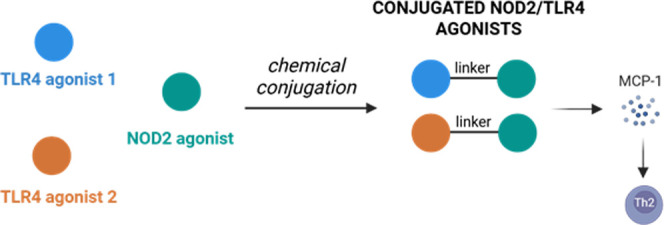

## Introduction

The innate immune system, the body’s first line of defense against infection, comprises immune cells that express various pattern recognition receptors (PRRs). These include Toll-like receptors (TLRs), nucleotide-binding oligomerization domain (NOD)-like receptors (NLRs), retinoic-acid-inducible gene I-like receptors, C-type lectin receptors, and the stimulator of interferon genes. Upon activation, PRRs initiate early immune signaling by inducing inflammatory cytokines, interferons, and costimulatory molecules, thereby shaping the magnitude and quality of the immune response. Consequently, PRR agonists represent promising candidates for the development of immunoenhancing therapeutics. Simultaneous activation of different PRRs can lead to signal amplification through receptor crosstalk, often producing synergistic effects that surpass the sum of individual receptor pathways.^2^ Notably, the chemical conjugation of distinct PRR agonists has emerged as an effective strategy to further enhance immunostimulatory activity compared to unconjugated mixtures.^1–3^

Among these, the coengagement of NOD2 and TLR4 has been extensively studied and shown to induce synergistic immune responses.^4,5^ NOD2, a cytosolic receptor of the NLR family, recognizes bacterial cell wall components such as muramyl dipeptide (MDP). Upon MDP binding, NOD2 oligomerizes and activates the nuclear factor κ-light-chain-enhancer of activated B cells (NF-κB) and mitogen-activated protein kinase signaling pathways, resulting in the expression of proinflammatory genes and cytokines.^6^ In contrast, TLR4, located on the cell surface, detects lipopolysaccharide (LPS), a key constituent of Gram-negative bacterial membranes, and signals through the MyD88 and TRIF pathways to induce inflammatory mediators.^7^ Recently, a first-generation conjugated NOD2/TLR4 agonist (compound **1**, Figure 1) was synthesized and shown to act as an adjuvant, synergistically enhancing T cell cytokine secretion relative to that of an unconjugated agonist mixture.^8^ In contrast, our previously constructed chimeric NOD2/TLR4 agonist (compound **2**, Figure 1) lacked immunostimulatory activity.^9^

**Figure 1:**
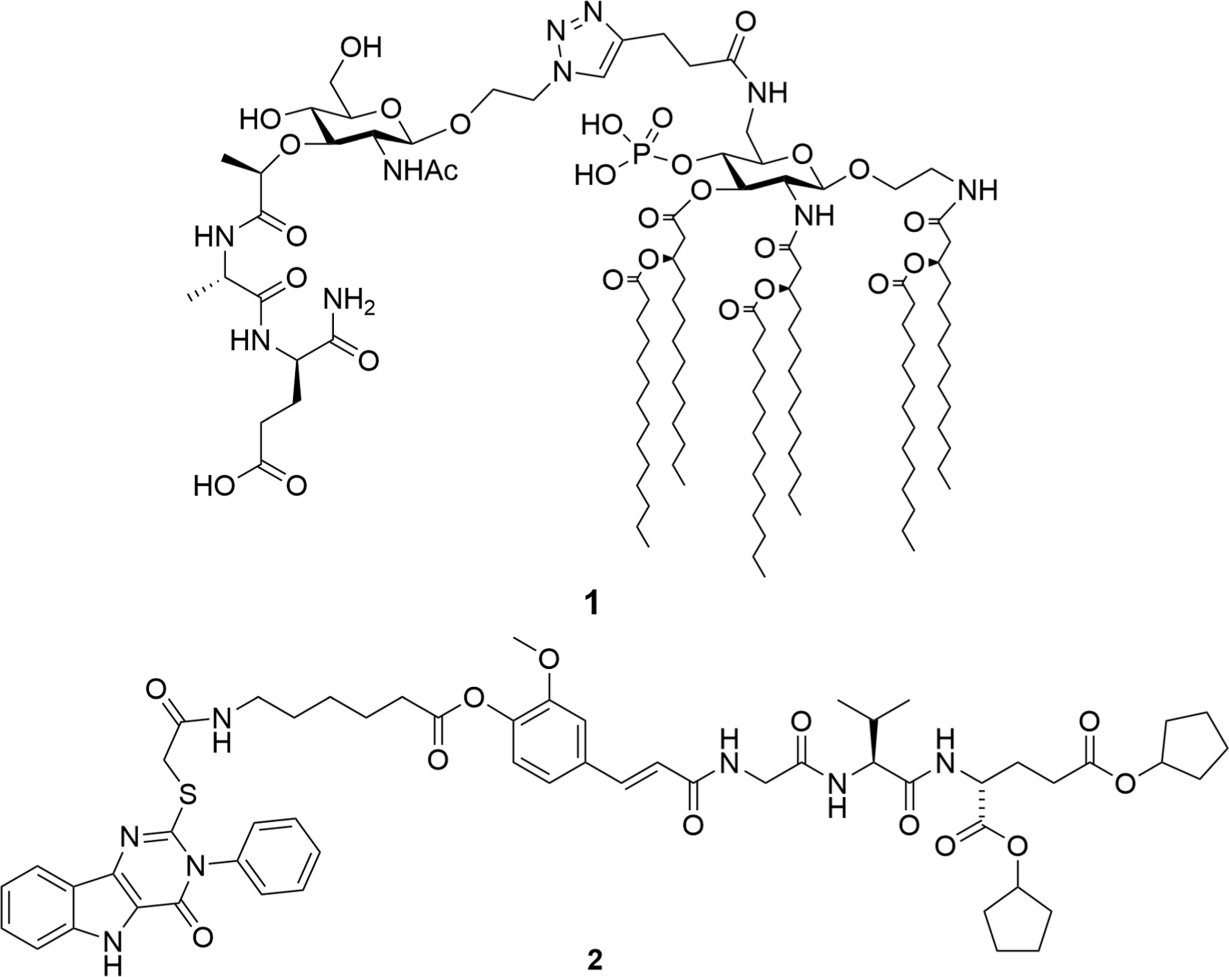
Chemical structures of the first reported conjugated NOD2/TLR4 agonists **1** ^8^ and **2**.^9^

This unexpected discrepancy, together with our prior findings on NOD2/TLR7 conjugates—where subtle changes in linker type and connectivity modulated immune activity and kinetic solubility^10,11^ —motivated us to further explore structure–activity relationships in conjugated NOD2/TLR4 agonists. In this study, we investigate how variations in TLR4 agonist structure and linker chemistry influence receptor-specific activity, immunomodulatory potential, and kinetic solubility of two newly synthesized series of conjugated NOD2/TLR4 agonists.

## Results and Discussion

As depicted in Figure 2, we synthesized a library of chimeric conjugates composed of our in-house flagship NOD2 agonist **3** ^10^ and two structurally distinct TLR4 agonists, merged by linkers commonly employed in bioconjugate chemistry.^12–14^ The two chemotypes of TLR4 agonists are represented by an α-aminoacyl amide Ugi compound **4** ^15^ and a carboxylic acid precursor **6** of the pyrimido­[5,4-*b*]­indole derivative **5**,^16^ which are synthetically accessible and well-suited to functionalization.

**Figure 2:**
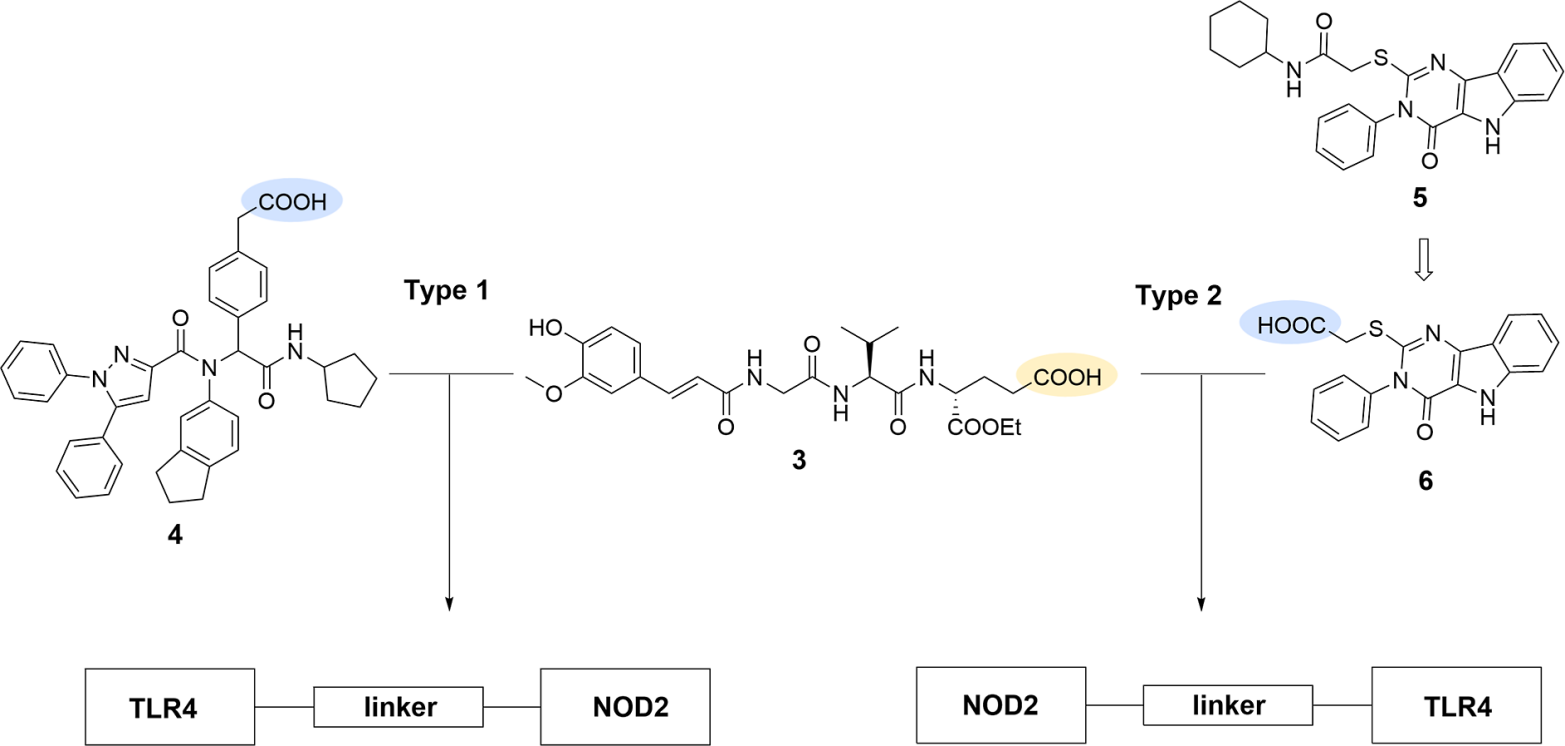
Design of novel conjugated NOD2/TLR4 agonists.

The length, flexibility, steric hindrance of the linker, as well as the linkage site and bonding type are critical determinants of the activity and physicochemical properties of conjugates, which in turn influence their pharmacokinetic profiles.^14^ In particular, PEG-based linkers have been incorporated into numerous FDA-approved therapeutics due to their ability to improve solubility, targeted delivery, cellular uptake, and circulation time postadministration.^12^ Accordingly, a variety of flexible alkyl- and PEG-based linkers were employed in the synthesis of the conjugates described here. In addition, we selected the more rigid 1-(piperidin-4-ylmethyl)­piperazine linker, previously used in the first proteolysis-targeting chimeras (PROTACs) to enter clinical trials, ARV-110 and ARV-471.^17^ The inclusion of piperazine moieties in PROTACs has been exploited to fine-tune solubility and conformational rigidity.^18^

The first series of conjugated NOD2/TLR4 agonists (compounds **17**–**21**), featuring TLR4 agonist **4** (Scheme 1), was synthesized by covalently attaching linkers to the carboxylic acid group of compound **4** using hexafluorophosphate azabenzotriazole tetramethyl uronium (HATU) as the coupling reagent, in the presence of *N*,*N*-diisopropylethylamine (DIPEA) and 4-dimethylaminopyridine (DMAP).^19^ The NOD2 agonist **3** was prepared according to our established procedure.^10^ It includes a tetrahydropyranyl (THP)-protected phenolic OH to prevent undesired cross-coupling and a free γ-carboxylic acid suitable for coupling with linker amines. TLR4 agonist **4** was synthesized as previously reported.^15^ All mono-Boc-protected diamine linkers (compounds **7**–**10**), with the exception of 1-(piperidin-4-ylmethyl)­piperazine (compound **11**), were synthesized as described in the Supporting Information.

**Scheme 1:**
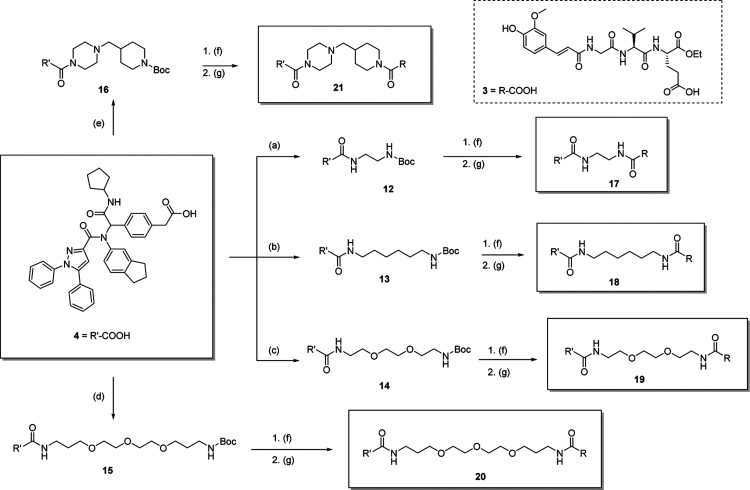
Synthesis of Conjugated NOD2/TLR4 Agonists **17–21** Based on the TLR4 Agonist **4** ^a^

a Reagents and conditions: (a) **9**, HATU, DIPEA, DCM, DMAP, rt, 16 h; (b) **10**, HATU, DIPEA, DCM, DMAP, rt, 16 h; (c) **7**, HATU, DIPEA, DCM, DMAP, rt, 16 h; (d) **8**, HATU, DIPEA, DCM, DMAP, rt, 16 h; (e) **11**, HATU, DIPEA, DCM, DMAP, rt, 16 h; (f) TFA/DCM (1:5), rt, 4 h; (g) **3** (THP-protected), COMU, DIPEA, DCM, DMAP, rt, 16 h.

These linkers were first coupled to compound **4** under HATU-mediated conditions, yielding amide intermediates **12**–**16**. Subsequent Boc deprotection via trifluoroacetic acid (TFA)-mediated acidolysis afforded intermediates bearing free primary or secondary amines, which were immediately coupled to the THP-protected NOD2 agonist **3** using COMU as the coupling agent, yielding final conjugates **17**–**21**. The THP protecting group was removed during workup.

Similarly, a second series of conjugated NOD2/TLR4 agonists (compounds **27–31**) based on the TLR4 agonist **6** (Scheme 2) was synthesized. The pyrimido­[5,4-*b*]­indole derivative **6** was prepared following the reported procedure.^16^ It was then coupled with five different linkers to afford intermediates **22–26**. After Boc deprotection via TFA-mediated acidolysis, these intermediates were coupled to the NOD2 agonist **3** to produce conjugates **27**–**31**.

**Scheme 2:**
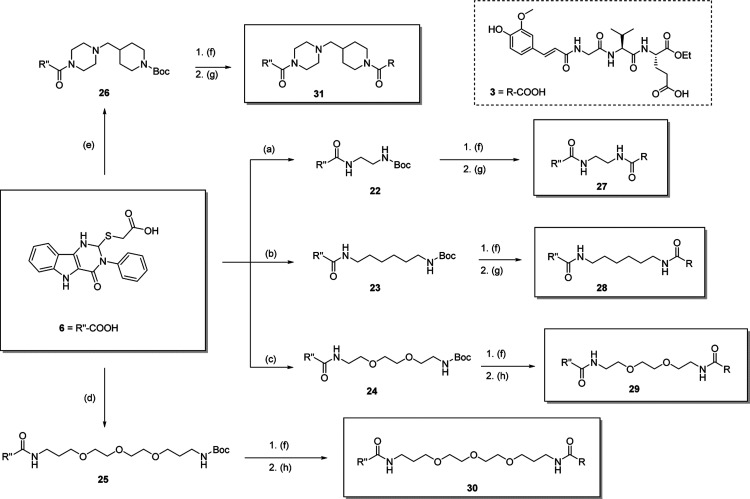
Synthesis of Conjugated NOD2/TLR4 Agonists **27–31** Based on TLR4 Agonist **6** ^a^

a Reagents and conditions: (a) **9**, HATU, DIPEA, DCM, DMAP, rt, 16 h; (b) **10**, HATU, DIPEA, DCM, DMAP, rt, 16 h; (c) **7**, HATU, DIPEA, DCM, DMAP, rt, 16 h; (d) **8**, HATU, DIPEA, DCM, DMAP, rt, 16 h; (e) **11**, HATU, DIPEA, DCM, DMAP, rt, 16 h; (f) TFA/DCM (1:5), rt, 4 h; (g) **3** (THP-protected), HATU, DIPEA, DCM, DMAP, rt, 16 h; (h) **3** (THP-protected), COMU, DIPEA, DCM, DMAP, rt, 16 h;

To evaluate the impact of different linkers on the physicochemical properties of the conjugates, we analyzed the kinetic solubility profiles of both conjugate series, their individual building blocks, and the structurally related conjugated NOD2/TLR4 agonist **2**,^9^ which features an alternative linkage site (Table 1). The amide-to-amide linking strategy, used to connect the carboxylic handles of both TLR4 agonists to the γ-carboxylic group of the NOD2 agonist, significantly influenced the kinetic solubility compared to the unconjugated agonists. Notably, shifting the linker attachment site on the NOD2 agonist from the phenolic OH group (reference conjugate **2**, kinetic solubility 0.35 μM) to the γ-carboxylic group generally improved solubility, as evidenced by conjugates **27**–**31**, which share the same TLR4 agonist. We next assessed the influence of linker identity on the kinetic solubility within each series. Conjugates **17**–**21**, incorporating the bulkier TLR4 agonist **4**, showed substantially reduced solubility—by nearly 3 orders of magnitude (0.32–2.08 μM)—relative to the individual building blocks **3** (414.8 μM) and **4** (237.6 μM). Among these, conjugate **21**, carrying the 1-(piperidin-4-ylmethyl)­piperazine moiety, exhibited the lowest solubility (0.33 μM). In contrast, most conjugates bearing the smaller TLR4 agonist **6** demonstrated kinetic solubilities similar to those of agonist building blocks **3** and **6** (254.1 μM). A notable exception was conjugate **31**, which also contained a 1-(piperidin-4-ylmethyl)­piperazine linker and displayed a low solubility of 6.68 μM. In this series, conjugate **27**, incorporating a short diaminoethane linker, exhibited a high solubility of 310.1 μM. The 3-unit PEG linker in conjugate **29** (237.9 μM) also conferred high solubility, outperforming conjugate **30** (122.4 μM), which contains a polyalkylether linker, and conjugate **28** (80.0 μM), which features a simple six-carbon alkyl chain.

**Table 1:** Kinetic Solubility^a^ Data and Receptor Activity^b^ Profile of Conjugated NOD2/TLR4 Agonists

compound	solubility in PBS, μM	NOD2 activation at 10 μM, fold increase	TLR4 activation at 10 μM (100 μM), fold increase
**3**	414.8^c^	3.06^d^	
**4**	237.6		7.23
**6**	254.1		1.22 (3.10)
**17**	0.76	3.66	5.10
**18**	1.01	2.43	1.46
**19**	2.14	3.06	2.78
**20**	1.50	3.53	2.68
**21**	0.33	3.31	5.30
**27**	310.1	2.17	1.06 (2.60)
**28**	80.0	1.65	1.06 (1.72)
**29**	237.9	1.60	1.00 (1.43)
**30**	122.4	1.55	1.05 (1.36)
**31**	6.68	3.33	1.05 (2.26)
**2**	0.35		

a Values obtained by HPLC solubility assay conducted as described in the literature.^20^

b SEAP activities were measured in NOD2-specific/TLR4-specific HEK-Blue cell supernatants after incubation for 18 h with compounds of interest (10 μM). The data are shown as fold increases of NF-κB transcriptional activity relative to the negative control (0.1% DMSO) and are expressed as means ± SEM of at least two independent experiments.

c As reported in previous studies.^11^

d Incubation for 18 h at 2 μM.^21^

To evaluate receptor-specific activity, all synthesized conjugates and their respective building blocks were tested using commercially available HEK-Blue reporter cell lines.^22^ These engineered HEK293 cells express an NF-κB-inducible secreted embryonic alkaline phosphatase (SEAP) reporter gene, enabling the real-time monitoring of immune receptor activation. Metabolic activity assays confirmed that none of the conjugates were toxic to hNOD2 or hTLR4 HEK-Blue cells (Figure 3). Stimulation of NOD2 and TLR4 reporter cell lines with conjugates **17**–**21** and **27**–**31** has been quantified colorimetrically (NOD2 and TLR4 activities at 10 μM are listed in Table 1 (for the TLR4 agonist **6** and **6**-incorporating conjugates, additional measurements at 100 μM are shown)). Dose–response profiles were also generated: NOD2 activity was assessed at 0.5, 1, 5, and 10 μM, while TLR4 activity was measured across two concentration ranges, 0.5–10 μM for conjugates **17**–**21** and 1–100 μM for conjugates **27**–**31**, due to the differing intrinsic potencies of the TLR4 agonists used (Figure 4). Overall, the conjugation of NOD2 and TLR4 agonists revealed nuanced effects on receptor activation with distinct impacts on both potency and efficiency across the two conjugate series (**17**–**21** and **27**–**31**).

**Figure 3:**
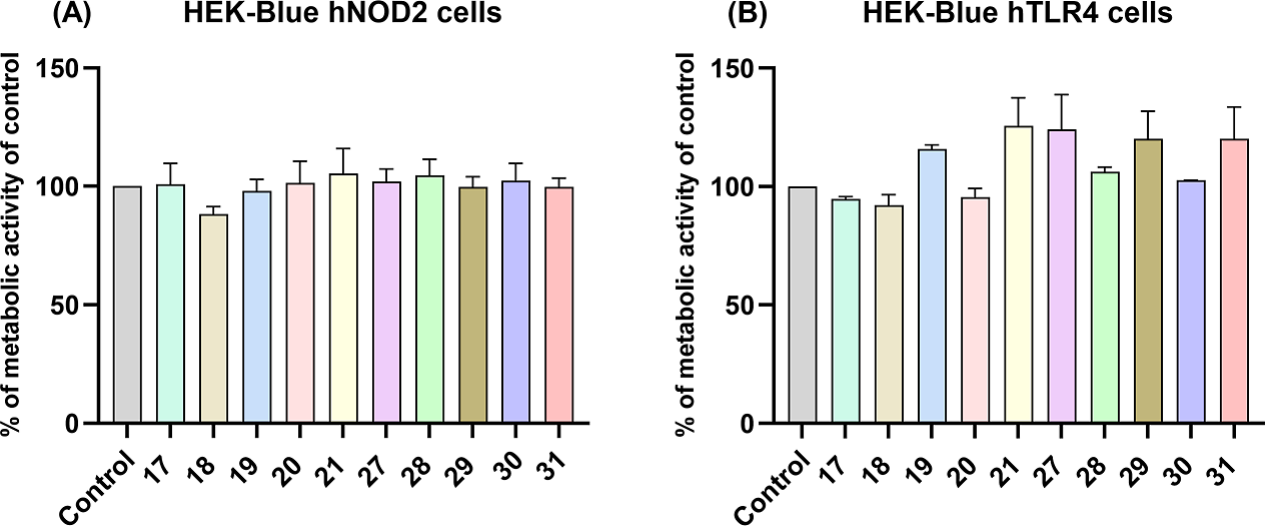
Metabolic activities/proliferation rates of HEK-Blue hNOD2 (A) and HEK-Blue hTLR4 (B) cells were measured after 18 h treatment with compounds (10 μM). Data are shown relative to the untreated control (0.1% DMSO). Data are means ± SEM of two independent experiments; ns, not significant versus control (one-way ANOVA post hoc Dunnett’s tests).

**Figure 4:**
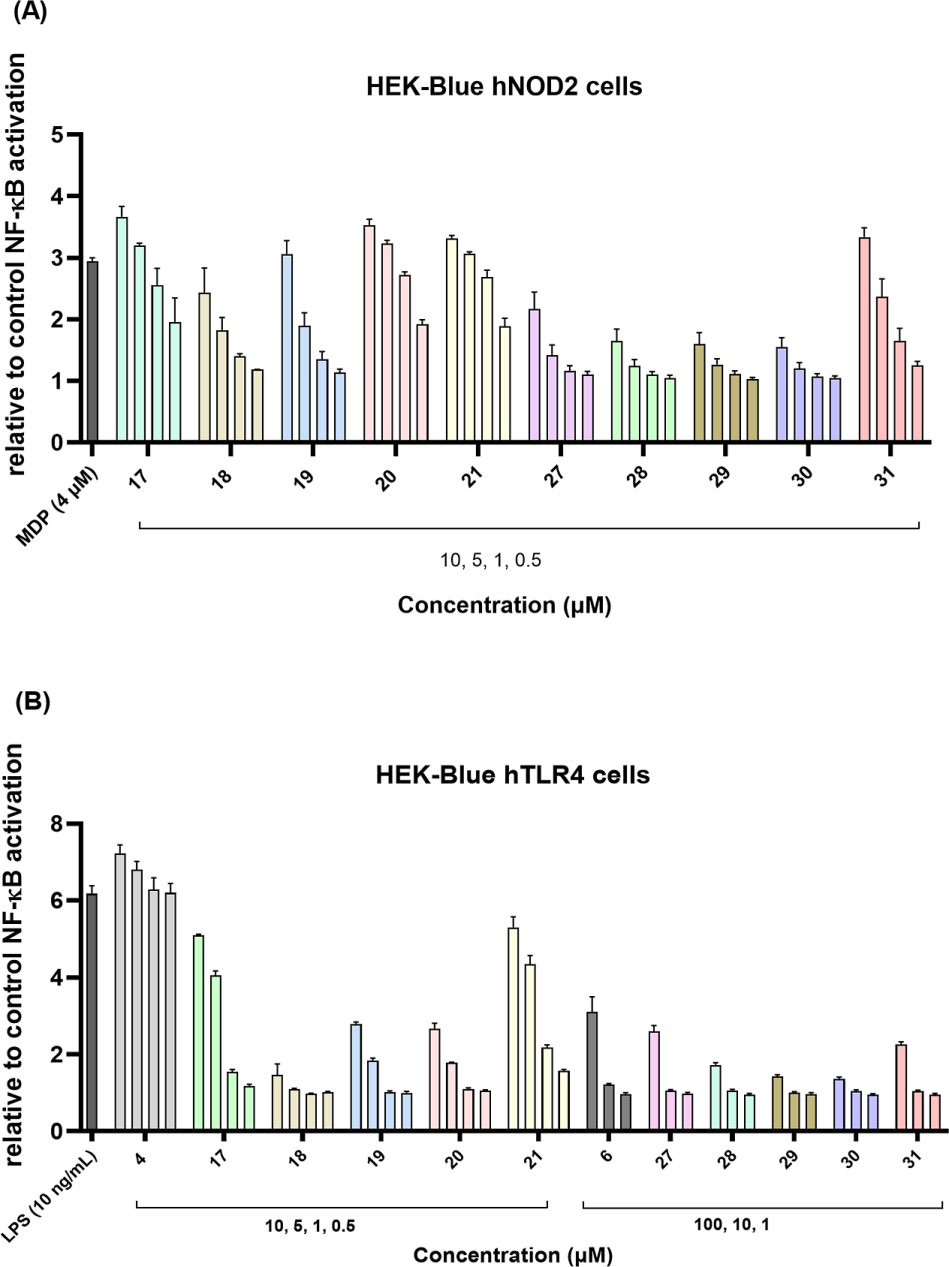
Concentration-dependent NF-κB transcriptional activities of conjugated NOD2/TLR4 agonists. SEAP activity was measured in (A) HEK-Blue hNOD2 cells after incubation for 18 h with MDP (positive control; 4 μM), and compounds (0.5–10 μM); and (B) HEK-Blue hTLR4 (B) cells after incubation for 18 h with LPS (positive control; 10 ng/mL) and compounds (0.5–10 μM or 1–100 μM concentration range as indicated). Data are shown as relative activities to the vehicle-treated control (0.1% DMSO) and are means ± SEM of three independent experiments.

For NOD2 activity, conjugation via the γ-carboxylic group in compounds **17**–**21** generally preserved the agonistic function of the individual NOD2 agonist **3** (EC_50_ of approximately 80 nM), with compounds **17**, **20**, and **21** exhibiting comparable efficiency to MDP at a single concentration, although their potencies were more markedly reduced. This decrease may be attributed to the unavailability of the γ-carboxylic group for interaction with NOD2, as the amide bond is not hydrolyzed in HEK cells.^9^ In contrast, the same conjugation strategy in compounds **27**–**31** led to a substantial loss of activity in both potency and efficiency, likely due to altered ligand orientation that impairs beneficial receptor interactions. Notably, despite bearing the bulkier TLR4 agonist **4**, compounds **17**–**21** exhibited higher NOD2 agonist activity than their counterparts incorporating TLR4 agonist **6**, potentially due to differential behavior of the TLR4 moiety, which may interfere with receptor-mediated endocytosis upon extracellular TLR4 binding. In both series, conjugates containing either the 1-(piperidin-4-ylmethyl)­piperazine spacer (compounds **17** and **27**) or the short diaminoethane linker (compounds **21** and **31**) generally demonstrated enhanced NOD2 activity compared to those with other linkers. Within compounds **17**–**21**, the addition of a third ethylene glycol unit in conjugate **20** also proved beneficial for the NOD2 activity. However, this modification did not improve the activity toward either receptor in the case of compound **30**.

With respect to the TLR4 agonist activity, conjugation in both series significantly reduced the potency of the parent agonists, reflecting the strength of their baseline activities. Nevertheless, a consistent trend was evident within each series, mirroring findings from the HEK-Blue hNOD2 assays. Conjugates **17**–**21** exhibited stronger TLR4 agonist activity than **27**–**31**, a difference attributable to the significantly higher baseline potency of compound **4** (EC_50_ = 3.3 nM; maximum activation 7.23-fold) compared to compound **6** (EC_50_ = 94 μM; maximum activation 3.10-fold), making it a more favorable TLR4 moiety for conjugation. For example, compound **21** activated TLR4 at 0.5 μM, compound **17** at 1.0 μM, and compounds **19** and **20** at 5 μM. The only exception was compound **18**, which showed weak TLR4 agonism at 10 μM. The maximum TLR4 activation of compound **21** (5.30-fold at 10 μM) was slightly lower than that of LPS and compound **4**. In contrast, conjugates **27**–**31** were only active at 100 μM and achieved significantly lower efficiencies (1.34- to 2.60-fold activation), consistent with their weaker TLR4 agonist component. As with NOD2 activation, the 1-(piperidin-4-ylmethyl)­piperazine (compounds **17** and **27**) and diaminoethane linkers (compounds **21** and **31**) appeared to enhance the TLR4 activity, supporting their further exploration in future conjugate designs.

Collectively, these findings highlight the pivotal role of linker chemistry, particularly its nature and flexibility, in modulating receptor activation and, by extension, biological activity. To further investigate their immune signatures, we evaluated the immunomodulatory properties of the conjugates in primary human peripheral blood mononuclear cells (PBMCs), comparing them to the corresponding unlinked agonist mixtures and focusing on their ability to modulate proinflammatory cytokine production in vitro (Figure 5). We tested our set of compounds at 1 μM, consistent with concentrations used in previous studies.^10,11^

**Figure 5:**
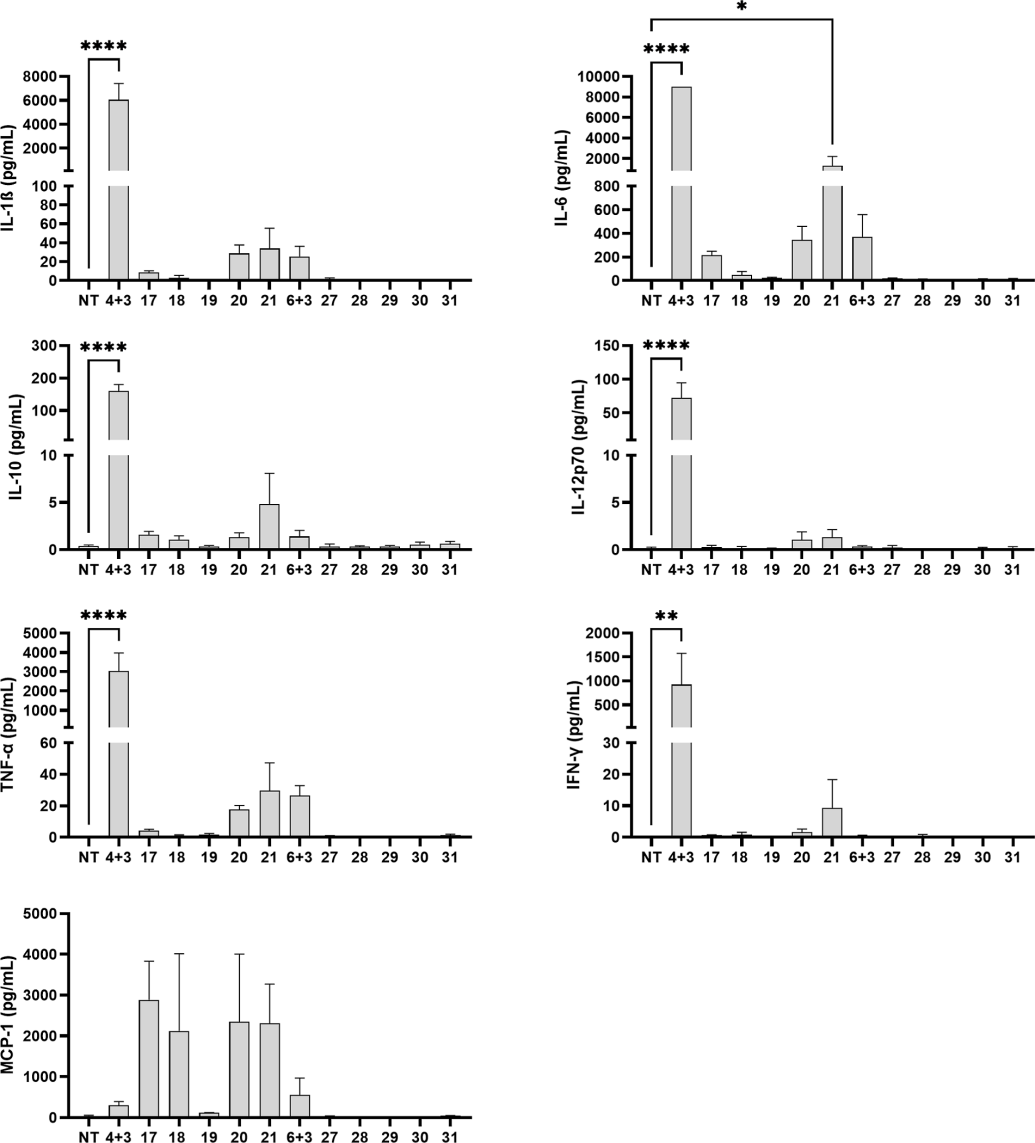
Effects of conjugate treatments on the release of cytokines from human PBMCs. Cytokine concentrations were measured after 18 h stimulation with unlinked mixtures of corresponding agonists (1 μM), conjugated agonists (1 μM), or the control vehicle (0.1% DMSO). Data are expressed as mean ± SEM of two independent experiments. *p ≤ 0.05, **p ≤ 0.01, ***p ≤ 0.001 versus control.

Overnight treatment of PBMCs with a combination of individual agonists **3** and **4** resulted in a substantial increase in the level of overall cytokine production. In contrast, costimulation with agonists **3** and **6** led to a much weaker response, likely due to the low intrinsic potency of TLR4 agonist **6**. Furthermore, the conjugated agonists from both series were generally less effective than the corresponding unlinked mixtures in inducing cytokine production. Chemical conjugation notably reduced the secretion of IL-12p70, IFN-γ, IL-6, IL-10, IL-1β, and TNF-α compared to the levels elicited by the unlinked agonist mixtures. Among the tested conjugates, only conjugate **21** moderately enhanced the production of IL-6, IL-10, TNF-α, and IFN-γ, broadly reflecting the results obtained in the reporter gene assays. In contrast, conjugates **27**–**31**, which incorporated TLR4 agonist **6**, were inactive and did not induce detectable cytokine responses, consistent with the previously reported inactivity of compound **2**.^9^ Interestingly, these findings contrast with earlier reports on compound **1**, which enhanced TNF-α and IFN-γ secretion by CD4^+^ T cells by 1.6-fold and 1.2-fold, respectively, and by CD8^+^ T cells by 3.3-fold, relative to the individual agonist mixture (MDP and lipid A mimetic).^8^ These discrepancies may stem from differences in the in vitro models used, variations in the intrinsic properties of the individual components, or conformational and steric constraints introduced by conjugation that impair receptor engagement. Notably, a distinct synergistic effect on monocyte chemoattractant protein-1 (MCP-1) secretion was observed for conjugates **17**–**21** (with the exception of compound **19**), all of which contain TLR4 agonist **4**. These conjugates induced a pronounced, albeit not statistically significant, increase in the level of MCP-1 that exceeded the response elicited by the unlinked agonist mixture. MCP-1 promotes the recruitment of monocytes, memory T cells, dendritic cells, and NK cells to sites of infection or inflammation. Beyond its chemoattractant function, it also regulates T cell proliferation and Th2 differentiation, supporting humoral immunity and antibody production.^23–25^

## Conclusions

In conclusion, we successfully synthesized a series of conjugated NOD2/TLR4 agonists incorporating diverse linkers and TLR4-activating moieties. Our findings offer new insights into the structure–activity relationships of these conjugates. The best performing compounds exhibited moderate immunomodulatory activity, characterized by increased MCP-1 secretion and a concurrent suppression of the proinflammatory cytokine signature from primary human immune cells, indicating a Th2-skewed immune response in an in vitro setting. Comparative analysis of linker strategies highlighted their pivotal role in modulating both the physicochemical properties and biological activities of the conjugates. Notably, the amide-to-amide linkage strategy markedly enhanced compound solubility compared to the previously used amide-to-phenol ester approach. Both TLR4 agonists’ carboxylic acid groups were confirmed as effective attachment points, and the γ-carboxylic group of the NOD2 agonist—already established as a functionalization site—proved advantageous for conjugation. Interestingly, conjugate **21** with the lowest solubility also emerged as the most potent immunomodulator in the series. These findings provide foundational design principles for developing conjugated NOD2/TLR4 agonists with distinct solubility and immunological profiles. Further in vitro and in vivo investigations will be essential to assess their potential as vaccine adjuvants and better understand their therapeutic applicability.

## Experimental Section

### Materials

Chemicals were obtained from Sigma-Aldrich (St. Louis, MO, USA), BLD Pharm (Germany), Acros Organics (Geel, Belgium), ABCR (Germany), Enamine (Monmouth Junction, NJ, USA), and Apollo (Stockport, UK) and were used without further purification. Analytical TLC was performed on Merck 60 F254 silica gel plates (0.25 mm), with visualization using ultraviolet light and ninhydrin. Flash column chromatography was carried out on Merck silica gel 60 (particle size 240–400 mesh) and on Biotage Isolera One Flash Chromatograph using Biotage Sfär C18 D Duo 100 Å 30 μm 30 g. ^1^H and ^13^C NMR spectra were recorded at 400 and 100 MHz, respectively, on an AVANCE III spectrometer (Bruker Corporation, Billerica, MA, USA) in MeOD or DMSO-*d*_6_ with tetramethylsilane as the internal standard. Mass spectra were obtained using an Exactive Plus Orbitrap mass spectrometer (Thermo Fisher Scientific, Waltham, MA, USA) or on Expression CMS mass spectrometer (Advion Inc., Ithaca, NY, USA). Analytical UHPLC analyses were performed on a Dionex UltiMate 3000 Rapid Separation Binary System (Thermo Fisher Scientific, Waltham, MA, USA) equipped with an autosampler, a binary pump system, a photodiode array detector, a thermostated column compartment, and the Chromeleon Chromatography data system. The column used was Waters Acquity UPLC BEH C18 (1.7 μm, 2.1 × 50 mm), with a flow rate of 0.3 mL/min. The eluent was a mixture of 0.1% TFA in water (A) and acetonitrile (B), with a gradient of (%B): 0–10 min, 5–95%; 10–12 min, 95%; 12–12.5 min, 95–5%. The columns were thermostated at 40 °C. The purity of all biologically tested compounds was >95%.

### Characterization of Compounds

#### Ethyl *N* ^5^-(2-(2-(4-(2-(Cyclopentylamino)-1-(*N*-(2,3-dihydro-1*H*-inden-5-yl)-1,5-diphenyl-1*H*-pyrazole-3-carboxamido)-2-oxoethyl)­phenyl)­acetamido)­ethyl)-*N* ^2^-((*E*)-3-(4-hydroxy-3-methoxyphenyl)­acryloyl)­glycyl-l-valyl-d-glutaminate (**17**)

Compound **12** (115 mg, 147 μmol) was dissolved in a solution of TFA and DCM (1:5, v/v, 5 mL) and stirred at room temperature for 4 h. After solvent removal under reduced pressure, the resulting residue was triturated with diethyl ether (3 × 5 mL) to obtain Boc-deprotected compound **12**, which was then used without further purification. A solution of **3** (95.59 mg, 161.6 μmol) in dry DMF (3 mL) was prepared under an argon atmosphere and then cooled on ice. To the stirring solution were then added COMU (69.2 mg, 161.6 μmol) and DIPEA (64 μL, 367 μmol). After 30 min, crude Boc-deprotected compound **12** (100 mg, 146.9 μmol) and DMAP (2 mg, 16.37 μmol) were added to the reaction mixture, which was allowed to warm to room temperature and stirred overnight. The following day, 3 mL of 1 M HCl was added to the reaction mixture. After 30 min, the reaction mixture was diluted with DCM (20 mL) and extracted sequentially with 1 M HCl (3 × 10 mL), saturated NaHCO_3_ solution (3 × 10 mL), and brine (10 mL). The organic phase was dried over anhydrous Na_2_SO_4_, filtered, and concentrated under reduced pressure. The crude product was purified by reverse-phase column chromatography (Isolera system) using a gradient of 5–95% ACN in water containing 0.1% TFA to afford **17** as an amorphous solid (15.1 mg, 8% yield). ^1^H NMR (400 MHz, DMSO-*d*_6_): δ 9.45 (s, 1H), 8.42–8.33 (m, 1H), 8.20 (t, *J* = 5.7 Hz, 1H), 8.16–8.05 (m, 1H), 7.97 (t, *J* = 5.6 Hz, 1H), 7.87 (d, *J* = 9.1 Hz, 1H), 7.82–7.74 (m, 1H), 7.38–7.25 (m, 6H), 7.17–7.12 (m, 1H), 7.09–6.97 (m, 6H), 6.95–6.90 (m, 3H), 6.82–6.73 (m, 1H), 6.61–6.52 (m, 1H), 6.23–6.19 (m, 1H), 4.29–4.12 (m, 2H), 4.13–3.98 (m, 3H), 3.90 (d, *J* = 5.7 Hz, 1H), 3.80 (s, 3H), 3.73 (s, 1H), 3.53–3.40 (m, 7H), 3.28 (s, 2H), 3.05 (q, *J* = 6.5 Hz, 4H), 2.74 (t, *J* = 7.6 Hz, 2H), 2.67–2.63 (m, 2H), 2.16–2.06 (m, 2H), 2.05–1.88 (m, 3H), 1.87–1.72 (m, 3H), 1.64–1.56 (m, 4H), 1.45 (s, 4H), 1.33–1.21 (m, 2H), 1.17 (t, *J* = 7.1 Hz, 3H), 0.88–0.77 (m, 5H). ^13^C NMR (100 MHz, MeOD): δ 174.96, 173.99, 173.00, 172.62, 172.41, 171.79, 169.78, 165.69, 150.08, 149.31, 148.89, 148.42, 148.04, 145.56, 145.19, 142.94, 140.71, 140.48, 139.39, 137.16, 134.59, 133.66, 131.95, 130.83, 130.36, 130.00, 129.95, 129.81, 129.71, 129.62, 129.33, 128.48, 128.27, 128.04, 126.41, 125.47, 124.74, 123.45, 121.67, 120.28, 118.05, 116.52, 116.19, 115.76, 114.51, 113.06, 111.72, 110.38, 66.95, 62.50, 61.04, 56.44, 52.97, 52.75, 44.03, 43.48, 40.06, 38.97, 33.51, 33.34, 32.99, 31.40, 31.40, 30.72, 27.73, 26.78, 24.82, 24.79, 19.73, 18.22, 14.51. HRMS *m*/*z*: calcd for C_66_H_75_N_9_O_11_, 1170.5659 (M + H)^+^; found, 1170.5623.

#### Ethyl *N* ^5^-(6-(2-(4-(2-(Cyclopentylamino)-1-(*N*-(2,3-dihydro-1*H*-inden-5-yl)-1,5-diphenyl-1*H*-pyrazole-3-carboxamido)-2-oxoethyl)­phenyl)­acetamido)­hexyl)-*N* ^2^-((*E*)-3-(4-hydroxy-3-methoxyphenyl)­acryloyl)­glycyl-l-valyl-d-glutaminate (**18**)

Compound **13** (119 mg, 142.4 μmol) was dissolved in a solution of TFA and DCM (1:5, v/v, 5 mL) and stirred at room temperature for 4 h. After solvent removal under reduced pressure, the resulting residue was triturated with diethyl ether (3 × 5 mL) to obtain Boc-deprotected compound **13**, which was then used without further purification. A solution of **3** (88.31 mg, 149.26 μmol) in dry DMF (3 mL) was prepared under an argon atmosphere and cooled on ice. To the stirring solution were then added COMU (61.26 mg, 149.26 μmol) and DIPEA (59 μL, 339 μmol). After 30 min, crude Boc-deprotected compound **13** (100 mg, 135.7 μmol) and DMAP (2 mg, 16.37 μmol) were added to the reaction mixture, which was allowed to warm to room temperature and stirred overnight. The following day, 3 mL of 1 M HCl was added to the reaction mixture. After 30 min, the reaction mixture was diluted with DCM (20 mL) and extracted sequentially with 1 M HCl (3 × 10 mL), saturated NaHCO_3_ solution (3 × 10 mL), and brine (10 mL). The organic phase was dried over anhydrous Na_2_SO_4_, filtered, and concentrated under reduced pressure. The crude product was purified by reversed-phase column chromatography (Isolera system) using a gradient of 5–95% ACN in water containing 0.1% TFA to afford **18** as an amorphous solid (25 mg, 15% yield). ^1^H NMR (400 MHz, DMSO-*d*_6_): δ 8.38 (t, *J* = 8.2 Hz, 1H), 8.20 (t, *J* = 5.8 Hz, 1H), 8.16–8.05 (m, 1H), 7.94 (t, *J* = 5.1 Hz, 1H), 7.90–7.73 (m, 2H), 7.38–7.26 (m, 7H), 7.16–7.14 (m, 1H), 7.11–6.99 (m, 7H), 6.95–6.91 (m, 2H), 6.81–6.73 (m, 1H), 6.64 (d, *J* = 7.9 Hz, 1H), 6.60–6.52 (m, 1H), 6.23–6.18 (m, 1H), 4.29–4.12 (m, 3H), 4.11–3.97 (m, 4H), 3.92–3.82 (m, 2H), 3.82–3.70 (m, 5H), 3.28 (s, 3H), 2.98 (t, *J* = 6.4 Hz, 4H), 2.78–2.61 (m, 6H), 2.39 (t, *J* = 6.6 Hz, 1H), 2.14–2.06 (m, 2H), 1.96 (m, 4H), 1.81–1.72 (m, 2H), 1.63–1.04 (m, 21H), 0.88–0.79 (m, 6H). ^13^C NMR (100 MHz, MeOD): δ 176.09, 174.46, 173.78, 173.54, 173.01, 172.98, 172.42, 172.17, 171.83, 169.70, 165.69, 150.06, 149.30, 148.89, 148.07, 145.89, 145.51, 145.14, 144.55, 142.85, 140.71, 140.26, 139.35, 137.40, 134.56, 133.67, 131.97, 130.82, 130.42, 130.00, 129.79, 129.70, 129.61, 129.32, 128.54, 128.07, 126.43, 124.70, 123.42, 121.66, 118.10, 116.53, 116.20, 115.77, 114.42, 113.06, 111.75, 110.38, 66.86, 62.42, 60.67, 60.52, 56.44, 53.35, 52.73, 44.08, 43.52, 40.37, 39.02, 33.50, 33.35, 33.14, 32.37, 31.62, 31.50, 30.27, 30.23, 30.16, 27.98, 27.52, 27.46, 26.78, 24.78, 19.76, 18.13, 14.50. HRMS *m*/*z*: calcd for C_70_H_83_N_9_O_11_, 1226.62848 (M + H)^+^; found, 1226.62794.

#### Ethyl (16*S*)-1-(4-(2-(Cyclopentylamino)-1-(*N*-(2,3-dihydro-1*H*-inden-5-yl)-1,5-diphenyl-1*H*-pyrazole-3-carboxamido)-2-oxoethyl)­phenyl)-16-((*R*)-2-(2-((*E*)-3-(4-hydroxy-3-methoxyphenyl)­acrylamido)­acetamido)-3-methylbutanamido)-2,13-dioxo-6,9-dioxa-3,12-diazaheptadecan-17-oate (**19**)

Compound **14** (125.2 mg, 160.3 μmol) was dissolved in a solution of TFA and DCM (1:5, v/v, 5 mL) and stirred at room temperature for 4 h. After solvent removal under reduced pressure, the resulting residue was triturated with diethyl ether (3 × 5 mL) to obtain Boc-deprotected compound **14**, which was then used without further purification. A solution of **3** (84.64 mg, 143.1 μmol) in dry DMF (3 mL) was prepared under an argon atmosphere and cooled on ice. To the stirred solution were then added COMU (61.26 mg, 143.1 μmol) and DIPEA (57 μL, 325 μmol). After 30 min, crude Boc-deprotected compound **14** (100 mg, 130.1 μmol) and DMAP (2 mg, 16.37 μmol) were added to the reaction mixture, which was allowed to warm to room temperature and stirred overnight. The following day, 3 mL of 1 M HCl was added to the reaction mixture. After 30 min, the reaction mixture was diluted with DCM (20 mL) and extracted sequentially with 1 M HCl (3 × 10 mL), saturated NaHCO_3_ solution (3 × 10 mL), and brine (10 mL). The organic phase was dried over anhydrous Na_2_SO_4_, filtered, and concentrated under reduced pressure. The crude product was purified by reverse-phase column chromatography (Isolera system) using a gradient of 5–95% ACN in water containing 0.1% TFA to afford **19** as an amorphous solid (19.6 mg, 12% yield). ^1^H NMR (400 MHz, MeOD): δ 8.37 (d, *J* = 7.6 Hz, 1H), 8.07 (d, *J* = 7.1 Hz, 1H), 7.50–7.44 (m, 1H), 7.36–7.11 (m, 3H), 7.10–6.93 (m, 11H), 6.81–6.62 (m, 2H), 6.52 (d, *J* = 15.7 Hz, 1H), 6.23 (s, 1H), 5.89 (d, *J* = 12.5 Hz, 1H), 4.35 (s, 2H), 4.25–4.07 (m, 9H), 3.99–3.79 (m, 7H), 3.61–3.46 (m, 16H), 3.42 (m, 3H), 2.76 (m, 8H), 2.30–2.10 (m, 3H), 1.94 (m, 5H), 1.63 (m, 5H), 1.41–1.21 (m, 4H), 1.04–0.93 (m, 6H). ^13^C NMR (100 MHz, MeOD): δ 174.09, 173.80, 173.72, 172.91, 171.92, 171.70, 170.51, 168.95, 165.75, 163.91, 152.92, 148.07, 145.57, 145.18, 144.59, 142.84, 142.53, 141.67, 140.70, 139.48, 137.45, 137.21, 135.22, 134.70, 131.84, 130.79, 130.30, 130.02, 129.84, 129.69, 129.63, 129.50, 129.36, 129.13, 129.08, 128.42, 126.41, 124.74, 124.20, 123.44, 121.75, 121.59, 112.62, 110.34, 71.27, 70.51, 70.37, 67.37, 67.01, 62.47, 60.10, 60.07, 56.45, 56.41, 53.20, 52.74, 49.71, 49.50, 45.95, 44.03, 43.36, 40.40, 40.14, 33.34, 31.76, 31.74, 31.25, 27.24, 26.79, 24.82, 24.78, 20.44, 19.75, 18.06, 14.46. HRMS *m*/*z*: calcd for C_70_H_83_N_9_O_13_, 1258.61831 (M + H)^+^; found, 1258.61731.

#### Ethyl (21*R*)-1-(4-(2-(Cyclopentylamino)-1-(*N*-(2,3-dihydro-1*H*-inden-5-yl)-1,5-diphenyl-1*H*-pyrazole-3-carboxamido)-2-oxoethyl)­phenyl)-21-((*S*)-2-(2-((*E*)-3-(4-hydroxy-3-methoxyphenyl)­acrylamido)­acetamido)-3-methylbutanamido)-2,18-dioxo-7,10,13-trioxa-3,17-diazadocosan-22-oate (**20**)

Compound **15** (138.5 mg, 177 μmol) was dissolved in a solution of TFA and DCM (1:5, v/v, 5 mL) and stirred at room temperature for 4 h. After solvent removal under reduced pressure, the resulting residue was triturated with diethyl ether (3 × 5 mL) to obtain Boc-deprotected compound **15**, which was then used without further purification. A solution of **3** (77.38 mg, 130.8 μmol) in dry DMF (3 mL) was prepared under an argon atmosphere and cooled on ice. To the stirring solution were then added COMU (56.1 mg, 130.8 μmol) and DIPEA (52 μL, 297 μmol). After 30 min, crude Boc-deprotected compound **15** (100 mg, 118.9 μmol) and DMAP (2 mg, 16.37 μmol) were added to the reaction mixture, which was allowed to warm to room temperature and stirred overnight. The following day, 3 mL of 1 M HCl was added to the reaction mixture. After 30 min, the reaction mixture was diluted with DCM (20 mL) and extracted sequentially with 1 M HCl (3 × 10 mL), saturated NaHCO_3_ solution (3 × 10 mL), and brine (10 mL). The organic phase was dried over anhydrous Na_2_SO_4_, filtered, and concentrated under reduced pressure. The crude product was purified by reverse-phase column chromatography (Isolera system) using a gradient of 5–95% ACN in water containing 0.1% TFA to afford **20** as an amorphous solid (15.8 mg, 10% yield). ^1^H NMR (400 MHz, MeOD): δ 8.36 (d, *J* = 7.5 Hz, 1H), 8.20–7.84 (m, 2H), 7.54–7.42 (m, 1H), 7.41–7.20 (m, 11H), 7.16 (m, 7H), 7.11–6.92 (m, 9H), 6.79 (d, *J* = 7.7 Hz, 1H), 6.74–6.61 (m, 1H), 6.52 (d, *J* = 15.7 Hz, 1H), 6.24 (s, 1H), 5.89 (d, *J* = 12.2 Hz, 1H), 4.35 (s, 2H), 4.27–4.06 (m, 8H), 4.00–3.90 (m, 1H), 3.90–3.80 (m, 4H), 3.67–3.38 (m, 11H), 3.23 (s, 4H), 2.76 (m, 3H), 2.20 (m, 3H), 2.06–1.81 (m, 4H), 1.77–1.47 (m, 7H), 1.41–1.19 (m, 6H), 1.04–0.91 (m, 5H). ^13^C NMR (100 MHz, MeOD): δ 176.10, 174.55, 173.59, 173.00, 172.38, 171.83, 169.69, 165.72, 150.08, 149.32, 148.91, 148.08, 145.53, 145.16, 144.56, 142.85, 140.74, 140.25, 139.37, 137.37, 134.60, 131.99, 130.85, 130.43, 130.00, 129.83, 129.72, 129.62, 129.32, 128.56, 128.09, 126.43, 125.47, 124.72, 123.43, 121.68, 120.37, 118.12, 116.53, 116.20, 115.77, 114.43, 113.08, 111.76, 110.38, 71.52, 71.21, 69.85, 69.80, 66.87, 62.43, 60.60, 56.51, 56.45, 56.39, 53.41, 52.74, 44.07, 43.57, 38.06, 37.85, 33.52, 33.35, 33.16, 32.37, 31.65, 31.53, 30.74, 30.39, 30.31, 27.92, 26.80, 24.83, 24.79, 19.77, 18.09, 14.50. HRMS *m*/*z*: calcd for C_74_H_91_N_9_O_14_, 1330.67582 (M + H)^+^; found, 1330.67510.

#### Ethyl (2*R*)-5-(4-((4-(2-(4-(2-(Cyclopentylamino)-1-(*N*-(2,3-dihydro-1*H*-inden-5-yl)-1,5-diphenyl-1*H*-pyrazole-3-carboxamido)-2-oxoethyl)­phenyl)­acetyl)­piperazin-1-yl)­methyl)­piperidin-1-yl)-2-((*S*)-2-(2-((*E*)-3-(4-hydroxy-3-methoxyphenyl)­acrylamido)­acetamido)-3-methylbutanamido)-5-oxopentanoate (**21**)

Compound **16** (124.6 mg, 137.1 μmol) was dissolved in a solution of TFA and DCM (1:5, v/v, 5 mL) and stirred at room temperature for 4 h. After solvent removal under reduced pressure, the resulting residue was triturated with diethyl ether (3 × 5 mL) to obtain Boc-deprotected compound **16**, which was then used without further purification. A solution of **3** (80.94 mg, 136.8 μmol) in dry DMF (3 mL) was prepared under an argon atmosphere and cooled on ice. To the stirring solution were then added COMU (58.59 mg, 136.8 μmol) and DIPEA (54 μL, 311 μmol). After 30 min, crude Boc-deprotected compound **16** (100 mg, 124.37 μmol) and DMAP (2 mg, 16.37 μmol) were added to the reaction mixture, which was allowed to warm to room temperature and stirred overnight. The following day, 3 mL of 1 M HCl was added to the reaction mixture. After 30 min, the reaction was then diluted with DCM (20 mL) and extracted sequentially with saturated NaHCO_3_ solution (3 × 10 mL), water (3 × 10 mL), and brine (10 mL). The organic phase was dried over anhydrous Na_2_SO_4_, filtered, and concentrated under reduced pressure. The crude product was purified by reverse-phase column chromatography (Isolera system) using a gradient of 5–95% ACN in water containing 0.1% TFA to afford **21** as an amorphous solid (28.9 mg, 18% yield). ^1^H NMR (400 MHz, MeOD): δ 8.35–8.18 (m, 1H), 8.14–7.91 (m, 1H), 7.52–7.40 (m, 1H), 7.36–7.19 (m, 10H), 7.19–6.94 (m, 9H), 6.88–6.64 (m, 4H), 6.51 (d, *J* = 13.3 Hz, 1H), 6.25–6.17 (m, 1H), 6.02–5.83 (m, 1H), 4.58–4.34 (m, 2H), 4.17 (m, 4H), 4.00–3.73 (m, 6H), 3.14–2.91 (m, 3H), 2.85–2.59 (m, 4H), 2.54–2.37 (m, 3H), 2.30–2.11 (m, 4H), 2.07–1.47 (m, 13H), 1.43–1.31 (m, 3H), 1.31–1.09 (m, 11H), 1.02 (s, 6H). ^13^C NMR (100 MHz, CDCl_3_): δ: 169.31, 169.28, 169.15, 164.10, 146.96, 144.80, 144.77, 143.95, 143.93, 142.86, 139.62, 135.24, 135.14, 133.64, 133.59, 130.67, 129.98, 128.79, 128.58, 128.55, 128.14, 127.86, 127.83, 126.34, 126.30, 125.16, 124.18, 110.19, 77.36, 67.30, 64.69, 63.94, 53.80, 53.23, 53.13, 52.29, 51.70, 46.24, 45.04, 41.88, 40.84, 38.75, 33.71, 33.08, 33.04, 32.89, 32.68, 32.60, 30.80, 30.46, 29.84, 29.15, 25.92, 23.92, 23.88. HRMS *m*/*z*: calcd for C_74_H_88_N_10_O_11_, 1293.67068 (M + H)^+^; found, 1293.66990.

#### Ethyl *N* ^2^-((*E*)-3-(4-Hydroxy-3-methoxyphenyl)­acryloyl)­glycyl-d-valyl-*N* ^5^-(2-(2-((4-oxo-3-phenyl-4,5-dihydro-3*H*-pyrimido­[5,4-*b*]­indol-2-yl)­thio)­acetamido)­ethyl)-l-glutaminate (**27**)

Compound **22** (111 mg, 224.8 μmol) was dissolved in a solution of TFA and DCM (1:5, v/v, 5 mL) and stirred at room temperature for 4 h. After solvent removal under reduced pressure, the resulting residue was triturated with diethyl ether (3 × 5 mL) to obtain Boc-deprotected compound **22**, which was then used without further purification. A solution of **3** (82.70 mg, 139.8 μmol) in dry DMF (3 mL) was prepared under an argon atmosphere and cooled on ice. To the stirring solution were then added HATU (53.15 mg, 139.8 μmol) and DIPEA (55 μL, 317 μmol). After 30 min, crude Boc-deprotected compound **22** (50 mg, 127 μmol) and DMAP (2 mg, 16.37 μmol) were added to the reaction mixture, which was allowed to warm to room temperature and stirred overnight. The following day, 3 mL of 1 M HCl was added to the reaction mixture. After 30 min, the reaction mixture was diluted with DCM (20 mL) and extracted sequentially with 1 M HCl (3 × 10 mL), saturated NaHCO_3_ solution (3 × 10 mL), and brine (10 mL). The organic phase was dried over anhydrous Na_2_SO_4_, filtered, and concentrated under reduced pressure. The crude product was purified by reversed-phase column chromatography (Isolera system) using a gradient of 5–95% ACN in water containing 0.1% TFA to afford **27** as an amorphous solid (11.2 mg, 10% yield). ^1^H NMR (400 MHz, MeOD): δ 8.14–8.04 (m, 1H), 7.67–7.39 (m, 9H), 7.32–7.19 (m, 1H), 7.17–6.96 (m, 1H), 6.84–6.44 (m, 3H), 4.45–4.06 (m, 6H), 4.01–3.71 (m, 4H), 3.47 (t, *J* = 6.1 Hz, 1H), 3.25–3.15 (m, 1H), 3.04–2.95 (m, 2H), 2.92–2.69 (m, 3H), 2.55–2.38 (m, 2H), 2.31–1.98 (m, 6H), 1.92–1.81 (m, 1H), 1.36–1.10 (m, 7H), 1.04–0.87 (m, 6H). ^13^C NMR (100 MHz, DMSO): δ 172.08, 171.72, 171.28, 171.04, 169.06, 167.12, 154.94, 152.36, 148.36, 147.81, 147.35, 144.58, 139.47, 138.92, 137.21, 136.04, 132.07, 129.86, 129.55, 127.29, 126.32, 121.60, 120.38, 120.20, 119.26, 118.50, 115.64, 115.25, 112.84, 112.36, 110.91, 60.46, 57.43, 55.52, 51.63, 48.59, 38.21, 36.48, 31.35, 30.69, 29.00, 28.58, 26.52, 22.08, 19.16, 17.73, 14.01. HRMS *m*/*z*: calcd for C_44_H_50_N_8_O_10_S, 883.3457 (M + H)^+^; found, 883.3443.

#### Ethyl *N* ^2^-((*E*)-3-(4-Hydroxy-3-methoxyphenyl)­acryloyl)­glycyl-d-valyl-*N* ^5^-(6-(2-((4-oxo-3-phenyl-4,5-dihydro-3*H*-pyrimido­[5,4-*b*]­indol-2-yl)­thio)­acetamido)­hexyl)-l-glutaminate (**28**)

Compound **23** (131 mg, 239 μmol) was dissolved in a solution of TFA and DCM (1:5, v/v, 5 mL) and stirred at room temperature for 4 h. After solvent removal under reduced pressure, the resulting residue was triturated with diethyl ether (3 × 5 mL) to obtain Boc-deprotected compound **23**, which was then used without further purification. A solution of **3** (67.6 mg, 114.2 μmol) in dry DMF (3 mL) was prepared under an argon atmosphere and cooled on ice. To the stirring solution were then added COMU (48.9 mg, 114.2 μmol) and DIPEA (45 μL, 256 μmol). After 30 min, crude Boc-deprotected compound **23** (50 mg, 103.8 μmol) and DMAP (2 mg, 16.37 μmol) were added to the reaction mixture, which was allowed to warm to room temperature and stirred overnight. The following day, 3 mL of 1 M HCl was added to the reaction mixture. After 30 min, the reaction mixture was diluted with DCM (20 mL) and extracted sequentially with 1 M HCl (3 × 10 mL), saturated NaHCO_3_ solution (3 × 10 mL), and brine (10 mL). The organic phase was dried over anhydrous Na_2_SO_4_, filtered, and concentrated under reduced pressure. The crude product was purified by reverse-phase column chromatography (Isolera system) using a gradient of 5–95% ACN in water containing 0.1% TFA to afford **28** as an amorphous solid (17.7 mg, 17% yield). ^1^H NMR (400 MHz, MeOD) δ 8.15–8.08 (m, 1H), 7.63–7.39 (m, 5H), 7.30–7.22 (m, 1H), 7.08 (d, *J* = 2.0 Hz, 1H), 6.98 (dd, *J* = 8.2, 1.9 Hz, 1H), 6.83–6.73 (m, 2H), 6.67 (d, *J* = 8.1 Hz, 1H), 6.46 (d, *J* = 15.7 Hz, 1H), 4.38–4.30 (m, 1H), 4.22–4.09 (m, 3H), 3.97–3.76 (m, 10H), 3.35 (s, 1H), 3.20 (q, *J* = 7.0 Hz, 3H), 2.95–2.75 (m, 3H), 2.55–2.38 (m, 2H), 2.26–2.08 (m, 3H), 2.03–1.90 (m, 2H), 1.49–1.35 (m, 2H), 1.30–1.19 (m, 3H), 1.17–1.03 (m, 6H), 1.02–0.84 (m, 6H). HRMS *m*/*z*: calcd for C_48_H_58_N_8_O_10_S, 939.40694 (M + H)^+^; found, 939.40516.

#### Ethyl (*S*)-16-((*R*)-2-(2-((*E*)-3-(4-Hydroxy-3-methoxyphenyl)­acrylamido)­acetamido)-3-methylbutanamido)-2,13-dioxo-1-((4-oxo-3-phenyl-4,5-dihydro-3*H*-pyrimido­[5,4-*b*]­indol-2-yl)­thio)-6,9-dioxa-3,12-diazaheptadecan-17-oate (**29**)

Compound **24** (135.75 mg, 233.4 μmol) was dissolved in a solution of TFA and DCM (1:5, v/v, 5 mL) and stirred at room temperature for 4 h. After solvent removal under reduced pressure, the resulting residue was triturated with diethyl ether (3 × 5 mL) to obtain Boc-deprotected compound **24**, which was then used without further purification. A solution of **3** (72.4 mg, 122.3 μmol) in dry DMF (3 mL) was prepared under an argon atmosphere and cooled on ice. To the stirred solution were then added HATU (46.5 mg, 122.3 μmol) and DIPEA (49 μL, 279 μmol). After 30 min, crude Boc-deprotected compound **24** (50 mg, 111.2 μmol) and DMAP (2 mg, 16.37 μmol) were added to the reaction mixture, which was allowed to warm to room temperature and stirred overnight. The following day, 3 mL of 1 M HCl was added to the reaction mixture. After 30 min, the reaction mixture was diluted with DCM (20 mL) and extracted sequentially with 1 M HCl (3 × 10 mL), saturated NaHCO_3_ solution (3 × 10 mL), and brine (10 mL). The organic phase was dried over anhydrous Na_2_SO_4_, filtered, and concentrated under reduced pressure. The crude product was purified by reverse-phase column chromatography (Isolera system) using a gradient of 5–95% ACN in water containing 0.1% TFA to afford **29** as an amorphous solid (13.7 mg, 12% yield). ^1^H NMR (400 MHz, MeOD): δ 8.16–8.08 (m, 1H), 7.65–7.39 (m, 8H), 7.26 (dddd, *J* = 8.0, 6.9, 3.2, 1.1 Hz, 1H), 7.17–6.97 (m, 2H), 6.84–6.73 (m, 2H), 6.72–6.57 (m, 2H), 6.48 (d, *J* = 15.7 Hz, 1H), 4.37–4.27 (m, 1H), 4.25–4.05 (m, 4H), 4.05–3.72 (m, 8H), 3.59–3.33 (m, 9H), 3.30–3.20 (m, 2H), 2.88–2.76 (m, 1H), 2.50 (m, 1H), 2.28–2.10 (m, 4H), 2.03–1.90 (m, 1H), 1.31–1.17 (m, 4H), 1.04–0.88 (m, 8H). ^13^C NMR (100 MHz, MeOD): δ 174.74, 173.77, 172.99, 172.34, 172.12, 171.15, 169.69, 157.31, 153.83, 150.07, 149.30, 142.86, 141.08, 139.93, 137.27, 131.34, 130.88, 130.70, 129.05, 128.07, 123.38, 122.19, 121.87, 121.70, 120.52, 118.07, 116.51, 116.18, 113.77, 113.07, 111.73, 71.17, 71.08, 70.50, 62.43, 60.54, 56.42, 53.41, 44.03, 40.90, 40.31, 39.01, 37.09, 33.04, 32.34, 31.53, 27.85, 19.75, 18.08, 14.48. HRMS *m*/*z*: calcd for C_48_H_58_N_8_O_12_S, 971.39677 (M + H)^+^; found, 971.39549.

#### Ethyl (*R*)-21-((*S*)-2-(2-((*E*)-3-(4-Hydroxy-3-methoxyphenyl)­acrylamido)­acetamido)-3-methylbutanamido)-2,18-dioxo-1-((4-oxo-3-phenyl-4,5-dihydro-3*H*-pyrimido­[5,4-*b*]­indol-2-yl)­thio)-7,10,13-trioxa-3,17-diazadocosan-22-oate (**30**)

Compound **25** (151.1 mg, 236.2 μmol) was dissolved in a solution of TFA and DCM (1:5, v/v, 5 mL) and stirred at room temperature for 4 h. After solvent removal under reduced pressure, the resulting residue was triturated with diethyl ether (3 × 5 mL) to obtain Boc-deprotected compound **25**, which was then used without further purification. A solution of **3** (58.8 mg, 99.3 μmol) in dry DMF (3 mL) was prepared under an argon atmosphere and cooled on ice. To the stirring solution were then added COMU (42.5 mg, 99.3 μmol) and DIPEA (29 μL, 226 μmol). After 30 min, crude Boc-deprotected compound **25** (50 mg, 90.3 μmol) and DMAP (2 mg, 16.37 μmol) were added to the reaction mixture, which was allowed to warm to room temperature and stirred overnight. The following day, 3 mL of 1 M HCl was added to the reaction mixture. After 30 min, the reaction mixture was diluted with DCM (20 mL) and extracted sequentially with 1 M HCl (3 × 10 mL), saturated NaHCO_3_ solution (3 × 10 mL), and brine (10 mL). The organic phase was dried over anhydrous Na_2_SO_4_, filtered, and concentrated under reduced pressure. The crude product was purified by reverse-phase column chromatography (Isolera system) using a gradient of 5–95% ACN in water containing 0.1% TFA to afford **30** as an amorphous solid (10.4 mg, 11% yield). ^1^H NMR (400 MHz, MeOD): δ 8.01–7.92 (m, 2H), 7.49–7.26 (m, 6H), 7.16–7.07 (m, 2H), 6.96 (d, *J* = 2.0 Hz, 1H), 6.87 (dd, *J* = 8.3, 1.9 Hz, 1H), 6.65–6.62 (m, 1H), 6.54 (d, *J* = 8.0 Hz, 1H), 6.47 (dd, *J* = 8.0, 2.0 Hz, 1H), 6.35 (d, *J* = 15.7 Hz, 1H), 4.24–4.16 (m, 2H), 4.10–4.05 (m, 2H), 4.04–3.93 (m, 5H), 3.84–3.75 (m, 4H), 3.72–3.65 (m, 5H), 3.39–3.21 (m, 11H), 3.18–3.15 (m, 4H), 3.10–2.98 (m, 3H), 2.77 (s, 1H), 2.67 (m, 1H), 2.37 (m, 1H), 2.13–1.98 (m, 3H), 1.89–1.78 (m, 1H), 1.65–1.48 (m, 3H), 1.13–1.05 (m, 4H), 0.88–0.75 (m, 6H). ^13^C NMR (100 MHz, MeOD): δ 176.09, 174.55, 173.78, 173.01, 172.37, 172.14, 170.85, 169.69, 157.30, 153.81, 150.05, 149.29, 148.89, 145.88, 142.85, 141.07, 139.93, 137.27, 133.69, 131.32, 130.87, 130.70, 129.02, 128.07, 123.40, 122.18, 121.83, 121.66, 120.50, 118.08, 116.51, 116.18, 113.77, 113.06, 111.73, 71.42, 71.34, 71.14, 71.06, 69.80, 69.77, 62.43, 60.58, 56.43, 56.38, 53.44, 53.40, 44.04, 43.74, 40.42, 39.02, 38.33, 37.85, 37.29, 33.15, 32.35, 31.64, 31.52, 30.75, 30.34, 30.28, 27.95, 27.89, 19.75, 18.14, 18.08, 14.49. HRMS *m*/*z*: calcd for C_52_H_66_N_8_O_13_S, 1043.4543 (M + H)^+^; found, 1043.4528.

#### Ethyl (*S*)-2-((*R*)-2-(2-((*E*)-3-(4-Hydroxy-3-methoxyphenyl)­acrylamido)­acetamido)-3-methylbutanamido)-5-oxo-5-(4-((4-(2-((4-oxo-3-phenyl-4,5-dihydro-3*H*-pyrimido­[5,4-*b*]­indol-2-yl)­thio)­acetyl)­piperazin-1-yl)­methyl)­piperidin-1-yl)­pentanoate (**31**)

Compound **26** (121.3 mg, 196.6 μmol) was dissolved in a solution of TFA and DCM (1:5, v/v, 5 mL) and stirred at room temperature for 4 h. After solvent removal under reduced pressure, the resulting residue was triturated with diethyl ether (3 × 5 mL) to obtain Boc-deprotected compound **26**, which was then used without further purification. A solution of **3** (63 mg, 106.45 μmol) in dry DMF (3 mL) was prepared under an argon atmosphere and then cooled on ice. To the stirring solution were then added HATU (40.48 mg, 106.45 μmol) and DIPEA (42 μL, 242 μmol). After 30 min, crude Boc-deprotected compound **26** (100 mg, 96.8 μmol) and DMAP (2 mg, 16.37 μmol) were added to the reaction mixture, which was allowed to warm to room temperature and stirred overnight. The following day, 3 mL of 1 M HCl was added to the reaction mixture. After 30 min, the reaction was then diluted with DCM (20 mL) and extracted sequentially with saturated NaHCO_3_ solution (3 × 10 mL), water (3 × 10 mL), and brine (10 mL). The organic phase was dried over anhydrous Na_2_SO_4_, filtered, and concentrated under reduced pressure. The crude product was purified by reverse-phase column chromatography (Isolera system) using a gradient of 5–95% ACN in water containing 0.1% TFA to afford **31** as an amorphous solid (14.6 mg, 15% yield). ^1^H NMR (400 MHz, MeOD): δ 8.10–8.01 (m, 1H), 7.70–7.33 (m, 9H), 7.25 (t, *J* = 7.5 Hz, 1H), 7.14 (d, *J* = 7.1 Hz, 1H), 7.05 (d, *J* = 8.2 Hz, 1H), 6.87–6.63 (m, 3H), 6.50 (d, *J* = 15.6 Hz, 1H), 4.49–4.37 (m, 2H), 4.28–4.10 (m, 7H), 3.98–3.74 (m, 8H), 3.66–3.54 (m, 2H), 3.35 (s, 3H), 3.03–2.96 (m, 1H), 2.86–2.81 (m, 1H), 2.66 (s, 1H), 2.59–2.32 (m, 4H), 2.29–2.10 (m, 4H), 2.07–1.91 (m, 1H), 1.33–1.23 (m, 6H), 1.04–0.91 (m, 6H). ^13^C NMR (100 MHz, DMSO): δ: 171.80, 171.15, 169.63, 166.07, 154.95, 152.19, 148.37, 147.83, 147.35, 139.78, 138.96, 137.19, 135.90, 132.08, 130.04, 129.69, 129.45, 127.50, 126.26, 121.79, 120.37, 120.24, 120.19, 119.22, 115.62, 115.24, 112.96, 112.30, 110.83, 60.65, 55.57, 54.77, 51.54, 44.19, 40.52, 39.74, 37.19, 35.41, 31.24, 30.36, 29.12, 28.93, 28.48, 26.26, 24.45, 22.06, 19.07, 17.71, 14.00. HRMS *m*/*z*: calcd for C_52_H_63_N_9_O_10_S, 1006.4491 (M + H)^+^; found, 1006.4481.

### Biology

#### HEK-Blue hNOD2 and hTLR4 Cells

HEK-Blue hNOD2 and hTLR4 cells (Invivogen, San Diego, CA, USA) were cultured according to the manufacturer’s instructions in Dulbecco’s modified Eagle’s medium (Sigma-Aldrich, St. Louis, MO, USA) supplemented with 10% heat-inactivated fetal bovine serum (Gibco), 2 mM l-glutamine (Sigma-Aldrich), 100 U/mL penicillin (Sigma-Aldrich), 100 μg/mL streptomycin (Sigma-Aldrich), and 100 μg/mL Normocin (Invivogen) for two passages. All subsequent passages were cultured in medium additionally supplemented with 100 μg/mL Zeocin and 30 μg/mL Blasticidin (Invivogen) for HEK-Blue hNOD2 cells or 1× HEK-Blue Selection for HEK-Blue hTLR4 cells. The cells were incubated in a humidified atmosphere at 37 °C and 5% CO_2_.

#### Peripheral Blood Mononuclear Cells

Human PBMCs from healthy and consenting donors were isolated from heparinized blood by density gradient centrifugation with a Ficoll–Paque (Pharmacia, Sweden). The isolated cells were washed twice with PBS, resuspended in RPMI 1640 medium (Sigma-Aldrich, St. Louis, MO, USA) supplemented with 10% heat-inactivated fetal bovine serum (Gibco), 2 mM l-glutamine (Sigma-Aldrich), 100 U/mL penicillin (Sigma-Aldrich), and 100 μg/mL streptomycin (Sigma-Aldrich), and used in the assays.

#### Metabolic Activity Assay

The tested compounds were dissolved in DMSO and further diluted in culture medium to the desired final concentrations such that the final DMSO concentration never exceeded 0.1%. HEK-Blue NOD2 and TLR4 cells were seeded (40,000 cells/well) in 96-well plates in 100 μL of culture medium and treated with 10 μM of each compound or with the corresponding vehicle (0.1% DMSO; control cells). After 18 h of incubation (37 °C, 5% CO_2_), the metabolic activity was assessed using the CellTiter 96 Aqueous One Solution cell proliferation assay (Promega, Madison, WI, USA), according to the manufacturer’s instructions. The experiments were run in duplicate and repeated as two independent biological replicates. Statistical significance was determined with one-way ANOVA with subsequent Dunnett’s multiple comparisons test.

#### NOD2/TLR4 NF-kB Reporter Assay

HEK-Blue hNOD2 cells (25,000 cells/well) and TLR4 cells (40,000 cells/well) were seeded in 96-well plates in 100 μL of HEK-Blue detection medium (Invivogen, San Diego, CA, USA) and treated with compounds (at 0.5 μM, 1 μM, 5 μM and 10 μM in the case of HEK-Blue hNOD2 cells (all conjugates) or with the corresponding vehicle (0.1% DMSO) and in the case of HEK-Blue hTLR4 cells at two different concentration ranges, 0.5 μM, 1 μM, 5 μM and 10 μM (conjugates **17–21**) and 1 μM, 10 μM and 100 μM (conjugates **27–31**) or the corresponding vehicle (0.1% DMSO). After 18 h of incubation (37 °C, 5% CO_2_), secreted embryonic alkaline phosphatase (SEAP) activity was determined spectrophotometrically as the absorbance at 630 nm (BioTek Synergy microplate reader; Winooski, VT, USA). EC_50_ values were calculated using Prism software (version 10; GraphPad Software, CA, USA). The experiments were run in duplicate and repeated in three independent biological replicates.

#### Cytokine Release from Peripheral Blood Mononuclear Cells

Peripheral blood mononuclear cells were seeded (1 × 10^6^ cells/mL) in 96-well plates in 100 μL of growth medium and treated with the compounds (1 μM) or the corresponding vehicle (0.1% DMSO). Cell-free supernatants were collected after incubation for 18 h (37 °C, 5% CO_2_) and stored at −80 °C until tested. Cytokine concentrations were determined with the LEGENDplex HU Essential Immune Response Panel (Biolegend) on an Attune NxT flow cytometer (Thermo Fisher Scientific, Waltham, MA). Standard curves were generated using recombinant cytokines contained in the kit. The data were analyzed using the FlowJo (Tree Star, Inc., Ashland, OR) and Prism (GraphPad, San Diego, CA) software. The experiments were run in duplicate and repeated as two independent biological replicates. Statistical significance was determined with one-way ANOVA with subsequent Dunnett’s multiple comparisons test.

#### Statistics

The data were analyzed using Prism software (version 10; GraphPad Software, CA, USA). Statistical significance was determined according to the specific procedures outlined in each experiment. A *p*-value <0.05 was considered statistically significant.

## Supplementary Material



## Notes

Full experimental details and compound characterization of compounds (^1^H NMR spectra and HPLC traces) and kinetic solubility data (PDF)

## Abbreviations

Boc: *tert*-butyloxycarbonyl
COMU: (1-cyano-2-ethoxy-2-oxoethylidenaminooxy)­dimethylamino-morpholino-carbenium hexafluorophosphate
DC: dendritic cell
DIPEA: *N*,*N*-diisopropylethylamine
DMAP: 4-dimethylaminopyridine
HATU: hexafluorophosphate azabenzotriazole tetramethyl uronium
IL: interleukin
IFN-γ: interferon γ
LPS: lipopolysaccharide
MCP-1: monocyte chemoattractant protein-1
MDP: muramyldipeptide
NF-κB: nuclear factor κ-light-chain-enhancer of activated B cells
NK: natural killer
NLR: NOD-like receptor
NOD2: nucleotide-binding oligomerization domain-containing protein 2
PBMC: peripheral blood mononuclear cells
PRR: pattern-recognition receptor
PROTAC: proteolysis targeting chimera
SEAP: secreted embryonic alkaline phosphatase
THP: tetrahydropyranyl
TLR: Toll-like receptor
TNF-α: tumor necrosis factor-α
