# Supplementary material for: Modulating Receptor Activity, Immune Response, and Kinetic Solubility: The Impact of Linker Chemistry in Conjugated NOD2/TLR4 Agonists
Source: ACS Omega. 2025 Aug 22;10(34):39060–72. doi: 10.1021/acsomega.5c05358 (PMC12409580; doi:10.1021/acsomega.5c05358)
Supplement: Supplementary file 1 [file ao5c05358_si_001.pdf]

SUPPORTING INFORMATION

Modulating Receptor Activity, Immune Response, and Kinetic Solubility: The Impact of Linker Chemistry in Conjugated NOD2/TLR4 Agonists

Emiliano Paradiso, Špela Janež, Žiga Jakopin*

University of Ljubljana, Faculty of Pharmacy, Department of Pharmaceutical Chemistry, Aškerčeva 7, SI-1000 Ljubljana, Slovenia

*Corresponding Author

Žiga Jakopin

Phone: +386 1 4769 646

Fax: +386 1 4258 031

E-mail: ziga.jakopin@ffa.uni-lj.si

Table of Contents

1. Synthetic procedures 2

1.1 General synthetic procedures 2

1.2 Characterization of compounds 2

2. Representative NMR spectra 7

3. Representative UHPLC traces 26

4. Solubility data 36

5. High-Resolution Mass Spectra (HRMS) 38

1. Synthetic procedures

1.1 General synthetic procedures

1.1.1 General procedure A: Boc protection of diamines

To an ice-chilled stirred solution of diamine (5 eq) in DCM, di-tert-butyl dicarbonate (1 eq) in DCM was added dropwise. The stirring was continued overnight at room temperature, after which DCM was evaporated off *in vacuo* and saturated $NaHCO_3$ (60 mL) was added. The resulting mixture was extracted with DCM (2 × 50 mL) and the combined organic phases were washed with brine (20 mL). The resulting organic layer was dried over anhydrous Na_2SO_4 and concentrated *in vacuo*.

Scheme S1. Synthesis of linkers **7-10**. MonoBoc-protected linker **11** was purchased from BLDPharm.

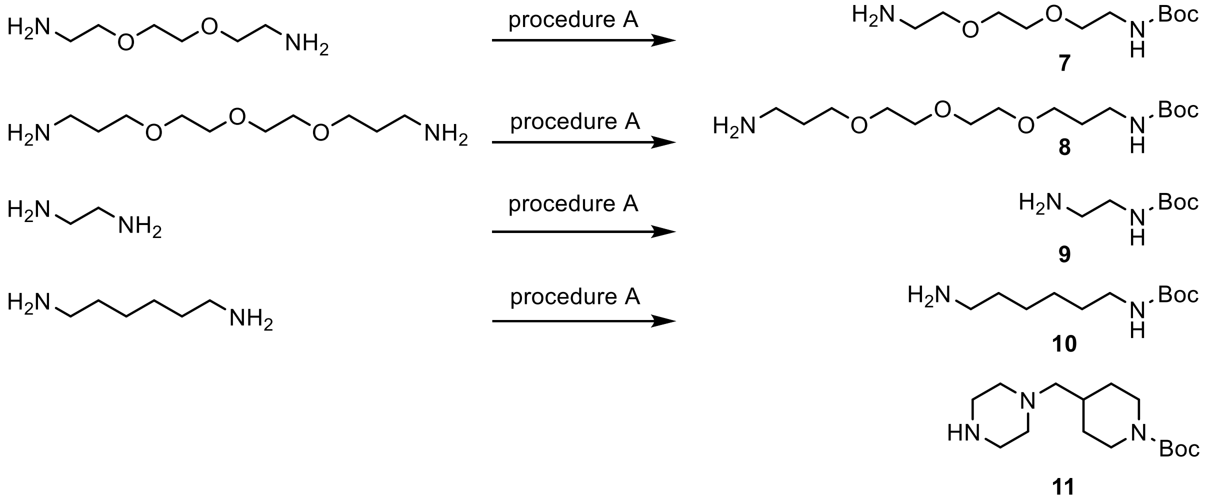

Reagents and conditions: Procedure A - Boc_2O, DCM, rt, 16h.

1.1.2 General procedure B: HATU-mediated coupling

To an ice-chilled stirred solution of the Boc-protected amine (1.2 eq) and carboxylic acid (1 eq) in DCM for **5**-based series (or DMF for **6**-based series), DIPEA (2.5 eq) and HATU (1.2 eq) were added. The mixture was allowed to warm to room temperature and the stirring continued overnight, after which it was diluted with DCM and washed with HCl 1M three times, $NaHCO_3$ sat. three times and brine once. Organic phase was then dried over Na_2SO_4 and concentrated *in vacuo* to give the desired compounds.

1.1.3 General procedure C: TFA-mediated acidolysis

The Boc-protected compound was added to an ice-chilled stirred mixture of TFA and DCM (1:5), and the mixture was allowed to warm to room temperature. After 4 h, the solvent was evaporated *in vacuo*. The residue was washed three times with diethyl ether.

1.2 Characterization of compounds

1.2.1 *Tert*-butyl (2-(2-(2-aminoethoxy)ethoxy)ethyl)carbamate (7)

Synthesized from 1,2-bis(2-aminoethoxy)ethane) (14.82 g, 100 mmol) using General procedure A. Colourless oil (3.24 g, 65%). ^1H NMR (400 MHz, DMSO-d_6) δ = 6.78 (q, *J*=8.5, 7.1 Hz, 1H), 3.49 (d, *J*=7.2 Hz, 4H), 3.36 (dt, *J*=10.2, 6.0 Hz, 4H), 3.06 (q, *J*=6.0 Hz, 2H), 2.64 (t, *J*=5.8 Hz, 2H), 1.37 (s, 9H).

1.2.2 *Tert*-butyl (3-(2-(2-(3-aminopropoxy)ethoxy)ethoxy)propyl)carbamate (8)

Synthesized from diethylene glycol bis(3-aminopropyl)ether) (15 g, 69 mmol) using General procedure A. Colourless oil (3.32 g, 74%). ^{1}H NMR (400 MHz, DMSO-d_6) δ = 6.78 (t, *J*=5.7 Hz, 1H), 3.56 – 3.32 (m, 11H), 3.02 – 2.86 (m, 2H), 2.57 (t, *J*=6.8 Hz, 2H), 1.57 (dt, *J* = 14.8, 6.7 Hz, 4H), 1.37 (s, 9H).

1.2.3 *Tert*-butyl (2-aminoethyl)carbamate (9)

Synthesized from 1,2-diaminoethane (10 g, 167 mmol) using General procedure A. Amorphous solid (2.927 g, 53%). ^{1}H NMR (400 MHz, DMSO-d_6) δ = 6.75 (dd, *J*=10.1, 4.8 Hz, 1H), 2.90 (q, *J*=6.3 Hz, 2H), 1.37 (s, 9H). (Signals for 2 protons are under solvent peak).

1.2.4 *Tert*-butyl (6-aminohexyl)carbamate (10)

Synthesized from 1,6-diaminohexane (10 g, 86 mmol) using General procedure A. White solid (2.47 g, 67%). ^{1}H NMR (400 MHz, DMSO-d_6) δ = 6.77 (t, *J*=5.7 Hz, 1H), 2.88 (td, *J*=7.1, 5.7 Hz, 2H), 1.46 – 1.13 (m, 19H). (Signals for 2 protons are under solvent peak).

1.2.5 *Tert*-butyl (2-(2-(4-(2-(cyclopentylamino)-1-(*N*-(2,3-dihydro-1*H*-inden-5-yl)-1,5-diphenyl-1*H*-pyrazole-3-carboxamido)-2-oxoethyl)phenyl)acetamido)ethyl)carbamate (12)

A solution of **4** (100 mg, 156.6 µmol) in dry DMF (3 mL) was prepared under an argon atmosphere and put on ice. To the stirring solution were then added HATU (102.9 mg, 187.9 µmol), DIPEA (68 µL, 391.5 µmol). After 30 min, the linker **9** (30.1 mg, 187.9 µmol), and DMAP (2 mg, 16.37 µmol) were add to the reactions and allowed to warm up. The reaction was then left at room temperature overnight. The following day, the mixture was diluted with dichloromethane (20 mL) and extracted sequentially with 1 M HCl (3 × 10 mL), saturated solution of $NaHCO_3$ (3 × 10 mL), and brine (10 mL). The organic phase was dried over anhydrous Na_2SO_4, filtered, and concentrated under reduced pressure. The crude product was purified by column chromatography (DCM/MeOH, 19:1) to afford **12** as pale oil (115 mg, 94%). ^{1}H NMR (400 MHz, DMSO-d_6) δ = 8.08 (d, *J*=7.0 Hz, 1H), 8.02 (t, *J*=5.6 Hz, 1H), 7.36 – 7.25 (m, 6H), 7.07 (s, 4H), 7.05 – 7.00 (m, 2H), 6.95 – 6.91 (m, 2H), 6.80 (t, *J*=5.7 Hz, 1H), 6.79 – 6.74 (m, 1H), 6.21 (s, 2H), 4.02 (q, *J*=6.7 Hz, 1H), 3.31 (s, 1H), 3.29 (s, 2H), 3.08 – 2.95 (m, 2H), 2.97 – 2.89 (m, 3H), 2.78 – 2.62 (m, 2H), 1.93 (p, *J*=7.4 Hz, 2H), 1.80 – 1.71 (m, 2H), 1.57 – 1.21 (m, 17H).

1.2.6 *Tert*-butyl (6-(2-(4-(2-(cyclopentylamino)-1-(*N*-(2,3-dihydro-1*H*-inden-5-yl)-1,5-diphenyl-1*H*-pyrazole-3-carboxamido)-2-oxoethyl)phenyl)acetamido)hexyl)carbamate (13)

A solution of **4** (100 mg, 156.6 µmol) in dry DMF (3 mL) was prepared under an argon atmosphere and put on ice. To the stirring solution were then added HATU (102.9 mg, 187.9 µmol), DIPEA (68 µL, 391.5 µmol). After 30 min, the linker **10** (50.9 mg, 187.9 µmol), and DMAP (2 mg, 16.37 µmol) were add to the reactions and allowed to warm up. The reaction was then left at room temperature overnight. The following day, the mixture was diluted with dichloromethane (20 mL) and extracted sequentially with 1 M HCl (3 × 10 mL), saturated solution of $NaHCO_3$ (3 × 10 mL), and brine (10 mL). The organic phase was dried over anhydrous Na_2SO_4, filtered, and concentrated under reduced pressure. The crude product was purified by column chromatography (DCM/MeOH, 19:1) to afford **13** as pale oil (119.25 mg, 91%). ^{1}H NMR (400 MHz, DMSO-d_6) δ = 8.07 (d, *J*=7.0 Hz, 1H), 7.94 (t, *J*=5.5 Hz, 1H), 7.42 – 7.26 (m, 6H), 7.17 – 6.97 (m, 7H), 6.99 – 6.85 (m, 3H), 6.76 (s, 1H), 6.33 – 6.11 (m, 2H), 4.02 (q, *J*=6.7 Hz, 1H), 3.05 – 2.94 (m, 2H), 2.93 – 2.82 (m, 3H), 2.78 – 2.70 (m, 2H), 2.00 – 1.85 (m, 2H), 1.85 – 1.67 (m, 2H), 1.67 – 1.13 (m, 23H).

1.2.7 *Tert*-butyl (2-(2-(2-(2-(4-(2-(cyclopentylamino)-1-(*N*-(2,3-dihydro-1*H*-inden-5-yl)-1,5-diphenyl-1*H*-pyrazole-3-carboxamido)-2-oxoethyl)phenyl)acetamido)ethoxy)ethoxy)ethyl)carbamate (14)

A solution of **4** (100 mg, 156.6 µmol) in dry DMF (3 mL) was prepared under an argon atmosphere and put on ice. To the stirring solution were then added HATU (102.9 mg, 187.9 µmol), DIPEA (68 µL, 391.5 µmol). After 30 min, the linker **7** (46.7 mg, 187.9 µmol), and DMAP (2 mg, 16.37 µmol) were add to the reactions and allowed to warm up. The reaction was then left at room temperature overnight. The following day, the mixture was diluted with dichloromethane (20 mL) and extracted sequentially with 1 M HCl (3 × 10 mL), saturated solution of $NaHCO_3$ (3 × 10 mL), and brine (10 mL). The organic phase was dried over anhydrous Na_2SO_4, filtered, and concentrated under reduced pressure. The crude product was purified by column chromatography (DCM/MeOH, 19:1) to afford **14** as pale oil (125.2 mg, 92%). ^{1}H NMR (400 MHz, DMSO-d_6) δ = 8.08 (t, *J*=5.2 Hz, 1H), 7.95 (s, 1H), 7.36 – 7.25 (m, 3H), 7.07 (s, 2H), 7.03 (s, 1H), 6.93 (s, 1H), 6.81 – 6.74 (m, 1H), 4.03 (q, *J*=6.7 Hz, 1H), 3.54 – 3.44 (m, 7H), 3.43 – 3.33 (m, 7H), 3.31 (s, 2H), 3.17 (q, *J*=5.7 Hz, 1H), 3.05 (q, *J*=5.9 Hz, 2H), 2.73 (m, 6H), 1.94 (q, *J*=7.5 Hz, 2H), 1.79 – 1.74 (m, 2H), 1.57 – 1.21 (m, 24H).

1.2.8 *Tert*-butyl (1-(4-(2-(cyclopentylamino)-1-(*N*-(2,3-dihydro-1*H*-inden-5-yl)-1,5-diphenyl-1*H*-pyrazole-3-carboxamido)-2-oxoethyl)phenyl)-2-oxo-7,10,13-trioxa-3-azahexadecan-16-yl)carbamate (15)

A solution of **4** (100 mg, 156.6 µmol) in dry DMF (3 mL) was prepared under an argon atmosphere and put on ice. To the stirring solution were then added HATU (102.9 mg, 187.9 µmol), DIPEA (68 µL, 391.5 µmol). After 30 min, the linker **8** (60.2 mg, 187.9 µmol), and DMAP (2 mg, 16.37 µmol) were add to the reactions and allowed to warm up. The reaction was then left at room temperature overnight. The following day, the mixture was diluted with dichloromethane (20 mL) and extracted sequentially with 1 M HCl (3 × 10 mL), saturated solution of $NaHCO_3$ (3 × 10 mL), and brine (10 mL). The organic phase was dried over anhydrous Na_2SO_4, filtered, and concentrated under reduced pressure. The crude product was purified by column chromatography (DCM/MeOH, 19:1) to afford **15** as pale oil (138.5 mg, 94%). ^{1}H NMR (400 MHz, DMSO-d_6) δ = 8.08 (d, J=7.1 Hz, 1H), 7.96 (t, J=5.6 Hz, 1H), 7.40 – 7.23 (m, 6H), 7.07 (s, 6H), 6.93 (s, 3H), 6.76 (t, J=5.6 Hz, 1H), 6.21 (s, 1H), 4.02 (q, J=6.7 Hz, 1H), 3.49 (m, 4H), 3.48 – 3.41 (m, 4H), 3.37 (m, 4H), 3.28 (s, 2H), 3.05 (q, J=6.6 Hz, 2H), 2.95 (q, J=6.7 Hz, 2H), 2.74 (t, J=7.4 Hz, 2H), 1.94 (q, J=7.4 Hz, 2H), 1.83 – 1.70 (m, 2H), 1.64 – 1.22 (m, 21H).

1.2.9 *Tert*-butyl 4-((4-(2-(4-(2-(cyclopentylamino)-1-(*N*-(2,3-dihydro-1*H*-inden-5-yl)-1,5-diphenyl-1*H*-pyrazole-3-carboxamido)-2-oxoethyl)phenyl)acetyl)piperazin-1-yl)methyl)piperidine-1-carboxylate (16)

A solution of **4** (100 mg, 156.6 µmol) in dry DMF (3 mL) was prepared under an argon atmosphere and put on ice. To the stirring solution were then added HATU (102.9 mg, 187.9 µmol), DIPEA (68 µL, 391.5 µmol). After 30 min, the linker **11** (53.3 mg, 187.9 µmol), and DMAP (2 mg, 16.37 µmol) were add to the reactions and allowed to warm up. The reaction was then left at room temperature overnight. The following day, the mixture was diluted with dichloromethane (20 mL) and extracted sequentially with water (3 × 10 mL), saturated solution of $NaHCO_3$ (3 × 10 mL), and brine (10 mL). The organic phase was dried over anhydrous Na_2SO_4, filtered, and concentrated under reduced pressure. The crude product was purified by column chromatography (DCM/MeOH, 19:1) to afford **16** as yellow solid (124.6 mg, 88%). ^{1}H NMR (400 MHz, DMSO-d_6) δ = 8.09 (d, *J*=7.0 Hz, 1H), 7.37 – 7.28 (m, 8H), 7.11 – 6.99 (m, 8H), 6.99 – 6.86 (m, 3H), 6.26 – 6.16 (m, 1H), 4.05 (q, *J*=6.7 Hz, 1H), 3.91 (m, 2H), 3.60 (s, 3H), 3.46 – 3.37 (m, 2H), 3.28 – 3.20 (m, 3H), 2.27 – 2.20 (m, 2H), 2.17 (s, 3H), 2.12 – 2.06 (m, 3H), 1.94 (p, *J*=7.5 Hz, 2H), 1.84 – 1.71 (m, 2H), 1.69 – 1.56 (m, 4H), 1.55 – 1.35 (m, 17H), 1.39 (s, 13H), 1.33 – 1.22 (m, 2H).

1.2.10 *Tert*-butyl (2-(2-((4-oxo-3-phenyl-4,5-dihydro-3H-pyrimido[5,4-*b*]indol-2-yl)thio)acetamido)ethyl)carbamate (22)

A solution of **6** (100 mg, 284.6 µmol) in dry DMF (3 mL) was prepared under an argon atmosphere and put on ice. To the stirring solution were then added HATU (129.9 mg, 341.5 µmol), DIPEA (124 µL, 711 µmol). After 30 min, the linker **9** (54.7 mg, 341.5 µmol), and DMAP (2 mg, 16.37 µmol) were add to the reactions and allowed to warm up. The reaction was then left at room temperature overnight. The following day, the mixture was diluted with dichloromethane (20 mL) and extracted sequentially with 1 M HCl (3 × 10 mL), saturated solution of $NaHCO_3$ (3 × 10 mL), and brine (10 mL). The organic phase was dried over anhydrous Na_2SO_4, filtered, and concentrated under reduced pressure. The crude product was purified by column chromatography (DCM/MeOH, 19:1) to afford **22** as yellow powder (111 mg, 79%). ^{1}H NMR (400 MHz, DMSO-d_6) δ = 12.08 (s, 1H), 8.28 (t, *J*=5.9 Hz, 1H), 8.03 (d, *J*=8.0 Hz, 1H), 7.65 – 7.56 (m, 3H), 7.55 – 7.42 (m, 4H), 7.33 – 7.17 (m, 1H), 6.81 – 6.75 (m, 1H), 5.95 (s, 1H), 3.89 (s, 2H), 3.13 – 3.05 (m, 2H), 3.02 – 2.86 (m, 8H), 2.69 (s, 2H), 1.37 (m, 18H).

1.2.11 *Tert*-butyl (6-(2-((4-oxo-3-phenyl-4,5-dihydro-3*H*-pyrimido[5,4-*b*]indol-2-yl)thio)acetamido)hexyl)carbamate (23)

A solution of **6** (100 mg, 284.6 µmol) in dry DMF (3 mL) was prepared under an argon atmosphere and put on ice. To the stirring solution were then added HATU (129.9 mg, 341.5 µmol), DIPEA (124 µL, 711 µmol). After 30 min, the linker **10** (77.9 mg, 341.5 µmol), and DMAP (2 mg, 16.37 µmol) were add to the reactions and allowed to warm up. The reaction was then left at room temperature overnight. The following day, the mixture was diluted with dichloromethane (20 mL) and extracted sequentially with 1 M HCl (3 × 10 mL), saturated solution of $NaHCO_3$ (3 × 10 mL), and brine (10 mL). The organic phase was dried over anhydrous Na_2SO_4, filtered, and concentrated under reduced pressure. The crude product was purified by column chromatography (DCM/MeOH, 19:1) to afford **23** as yellow powder (131 mg, 84%). ^{1}H NMR (400 MHz, MeOD) δ = 7.38 – 7.20 (m, 5H), 7.15 (q, *J*=8.2 Hz, 3H), 7.06 (d, *J*=7.1 Hz, 1H), 6.97 (d, *J*=7.4 Hz, 2H), 6.24 (s, 1H), 4.45 – 4.32 (m, 2H), 4.22 – 4.09 (m, 4H), 4.08 – 4.02 (m, 1H), 3.71 (m, 2H), 3.39 (s, 1H), 3.13 (t, *J*=7.0 Hz, 1H), 2.80 (t, *J*=7.4 Hz, 2H), 2.38 – 2.29 (m, 2H), 2.24 (t, *J*=7.5 Hz, 1H), 2.21 – 2.07 (m, 1H), 2.04 – 1.53 (m, 9H), 1.48 – 1.40 (m, 10H), 1.30 (s, 12H).

1.2.12 *Tert*-butyl (2-(2-(2-(2-((4-oxo-3-phenyl-4,5-dihydro-3*H*-pyrimido[5,4-*b*]indol-2-yl)thio)acetamido)ethoxy)ethoxy)ethyl)carbamate (24)

A solution of **6** (100 mg, 284.6 µmol) in dry DMF (3 mL) was prepared under an argon atmosphere and put on ice. To the stirring solution were then added HATU (129.9 mg, 341.5 µmol), DIPEA (124 µL, 711 µmol). After 30 min, the linker **7** (84.8 mg, 341.5 µmol), and DMAP (2 mg, 16.37 µmol) were add to the reactions and allowed to warm up. The reaction was then left at room temperature overnight. The following day, the mixture was diluted with dichloromethane (20 mL) and extracted sequentially with 1 M HCl (3 × 10 mL), saturated solution of $NaHCO_3$ (3 × 10 mL), and brine (10 mL). The organic phase was dried over anhydrous Na_2SO_4, filtered, and concentrated under reduced pressure. The crude product was purified by column chromatography (DCM/MeOH, 19:1) to afford **24** as yellow powder (135.75 mg, 82%). ^{1}H NMR (400 MHz, DMSO-d_6) δ = 12.10 (s, 1H), 8.31 (t, *J*=5.6 Hz, 1H), 8.04 (d, *J*=8.0 Hz, 1H), 7.66 – 7.54 (m, 3H), 7.55 – 7.41 (m, 4H), 7.31 – 7.18 (m, 1H), 6.76 (d, *J*=6.5 Hz, 1H), 3.91 (s, 2H), 3.53 – 3.45 (m, 2H), 3.46 – 3.38 (m, 6H), 3.23 (q, *J*=5.8 Hz, 2H), 3.04 (p, *J*=6.3 Hz, 3H), 1.36 (m, 12H).

1.2.13 *Tert*-butyl (14-oxo-15-((4-oxo-3-phenyl-4,5-dihydro-3*H*-pyrimido[5,4-*b*]indol-2-yl)thio)-3,6,9-trioxa-13-azapentadecyl)carbamate (25)

A solution of **6** (100 mg, 284.6 µmol) in dry DMF (3 mL) was prepared under an argon atmosphere and put on ice. To the stirring solution were then added HATU (129.9 mg, 341.5 µmol), DIPEA (124 µL, 711 µmol). After 30 min, the linker **8** (109.4 mg, 341.5 µmol), and DMAP (2 mg, 16.37 µmol) were add to the reactions and allowed to warm up. The reaction was then left at room temperature overnight. The following day, the mixture was diluted with dichloromethane (20 mL) and extracted sequentially with 1 M HCl (3 × 10 mL), saturated solution of $NaHCO_3$ (3 × 10 mL), and brine (10 mL). The organic phase was dried over anhydrous Na_2SO_4, filtered, and concentrated under reduced pressure. The crude product was purified by column chromatography (DCM/MeOH, 19:1) to afford **25** as yellow powder (130.8 mg, 83%). ^{1}H NMR (400 MHz, DMSO-*d*$_6$) δ = 8.22 (t, *J*=5.9 Hz, 1H), 8.04 (dt, *J*=8.1, 1.0 Hz, 1H), 7.63 – 7.56 (m, 3H), 7.55 – 7.43 (m, 5H), 7.29 – 7.21 (m, 1H), 6.75 (s, 4H), 5.76 (s, 7H), 3.54 – 3.35 (m, 21H), 3.05 – 2.89 (m, 8H), 1.68 – 1.52 (m, 6H), 1.36 (m, 14H).

1.2.14 *Tert*-butyl 4-((4-(2-((4-oxo-3-phenyl-4,5-dihydro-3*H*-pyrimido[5,4-*b*]indol-2-yl)thio)acetyl)piperazin-1-yl)methyl)piperidine-1-carboxylate (26)

A solution of **6** (100 mg, 284.6 µmol) in dry DMF (3 mL) was prepared under an argon atmosphere and put on ice. To the stirring solution were then added HATU (129.9 mg, 341.5 µmol), DIPEA (124 µL, 711 µmol). After 30 min, the linker **11** (88 mg, 341.5 µmol), and DMAP (2 mg, 16.37 µmol) were add to the reactions and allowed to warm up. The reaction was then left at room temperature overnight. The following day, the mixture was diluted with dichloromethane (20 mL) and extracted sequentially with water (3 × 10 mL), saturated solution of $NaHCO_3$ (3 × 10 mL), and brine (10 mL). The organic phase was dried over anhydrous Na_2SO_4, filtered, and concentrated under reduced pressure. The crude product was purified by column chromatography (DCM/MeOH, 19:1) to afford **26** as orange powder (121.3 mg, 76%). ^{1}H NMR (400 MHz, DMSO-*d*$_6$) δ = 12.12 (s, 1H), 8.04 (d, *J*=8.0 Hz, 1H), 7.66 – 7.55 (m, 3H), 7.56 – 7.41 (m, 4H), 7.24 (ddd, *J*=8.0, 6.8, 1.2 Hz, 1H), 4.25 (s, 2H), 3.92 (m, 2H), 3.67 – 3.62 (m, 2H), 3.46 – 3.41 (m, 2H), 2.49 – 2.43 (m, 11H), 2.31 – 2.27 (m, 2H), 2.16 (d, *J*=6.8 Hz, 2H), 1.69 (m, 3H), 1.39 (s, 10H), 0.95 (m, 2H).

2. Representative NMR spectra

Figure S1. Compound **17**: ^{1}H, 400 MHz, DMSO-d_6

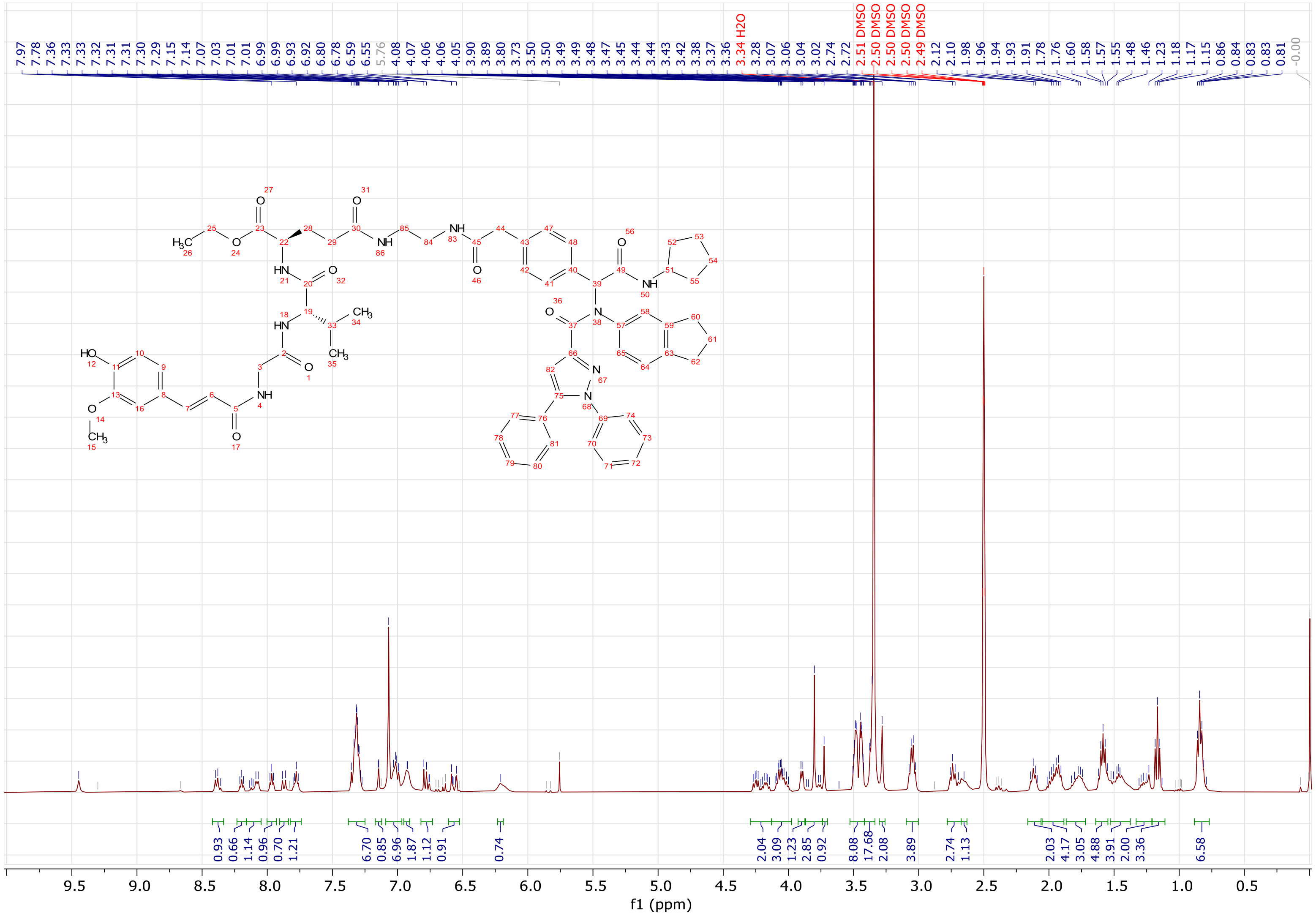

Figure S2. Compound **17**: ^{13}C, 400 MHz, MeOD

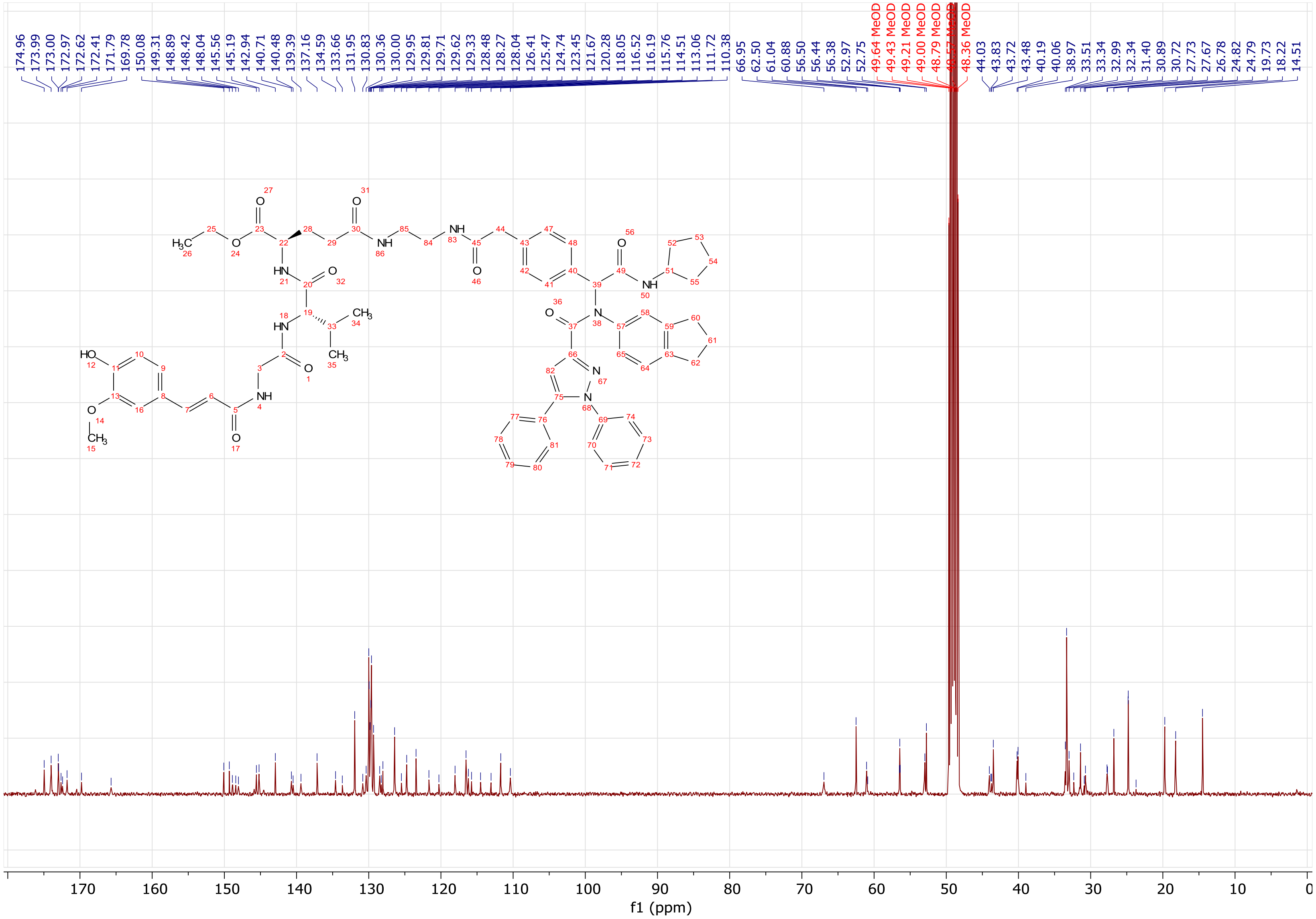

Figure S3. Compound **18**: ^{1}H, 400 MHz, DMSO-d_6

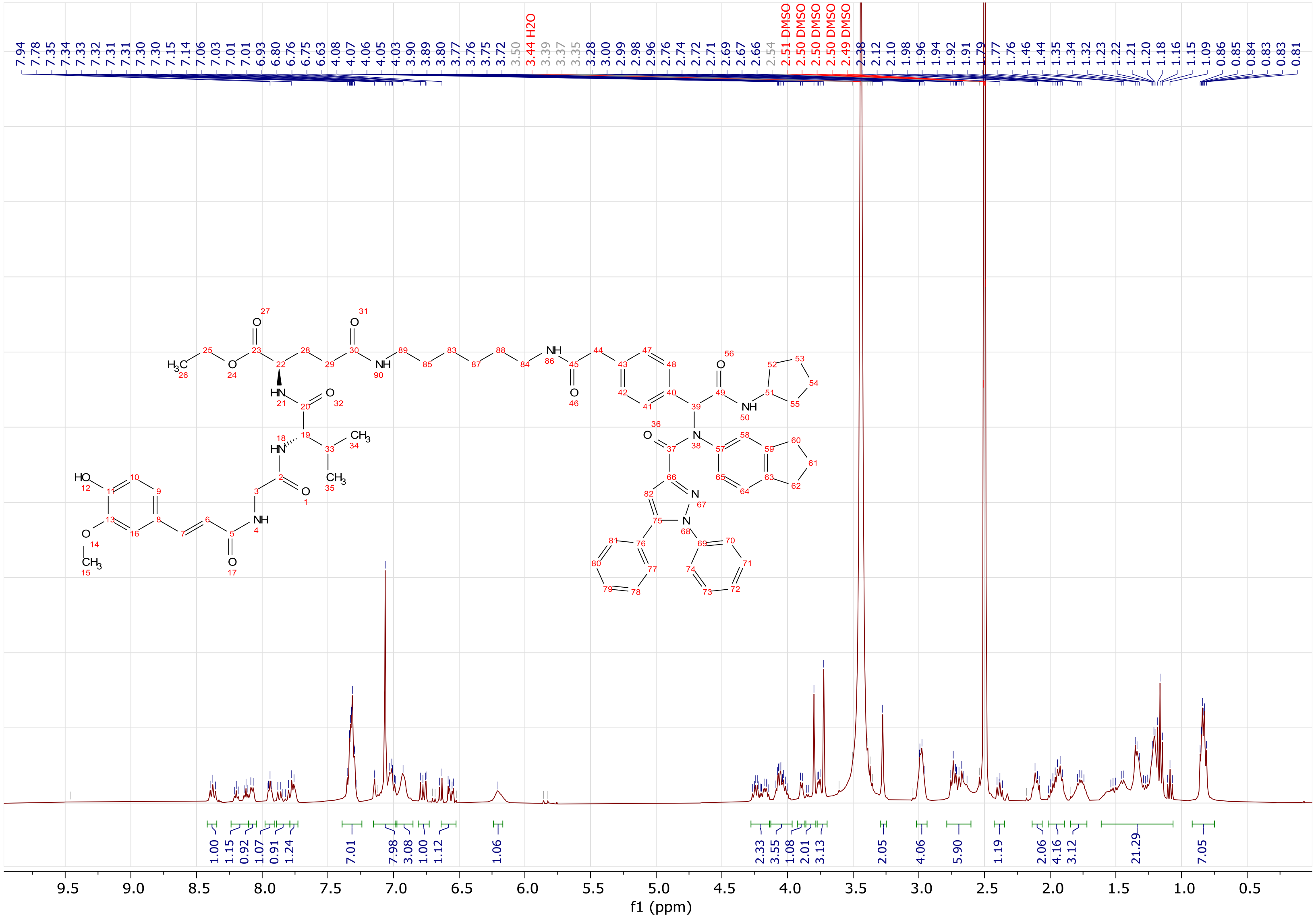

Figure S4. Compound **18**: ^{13}C, 400 MHz, MeOD

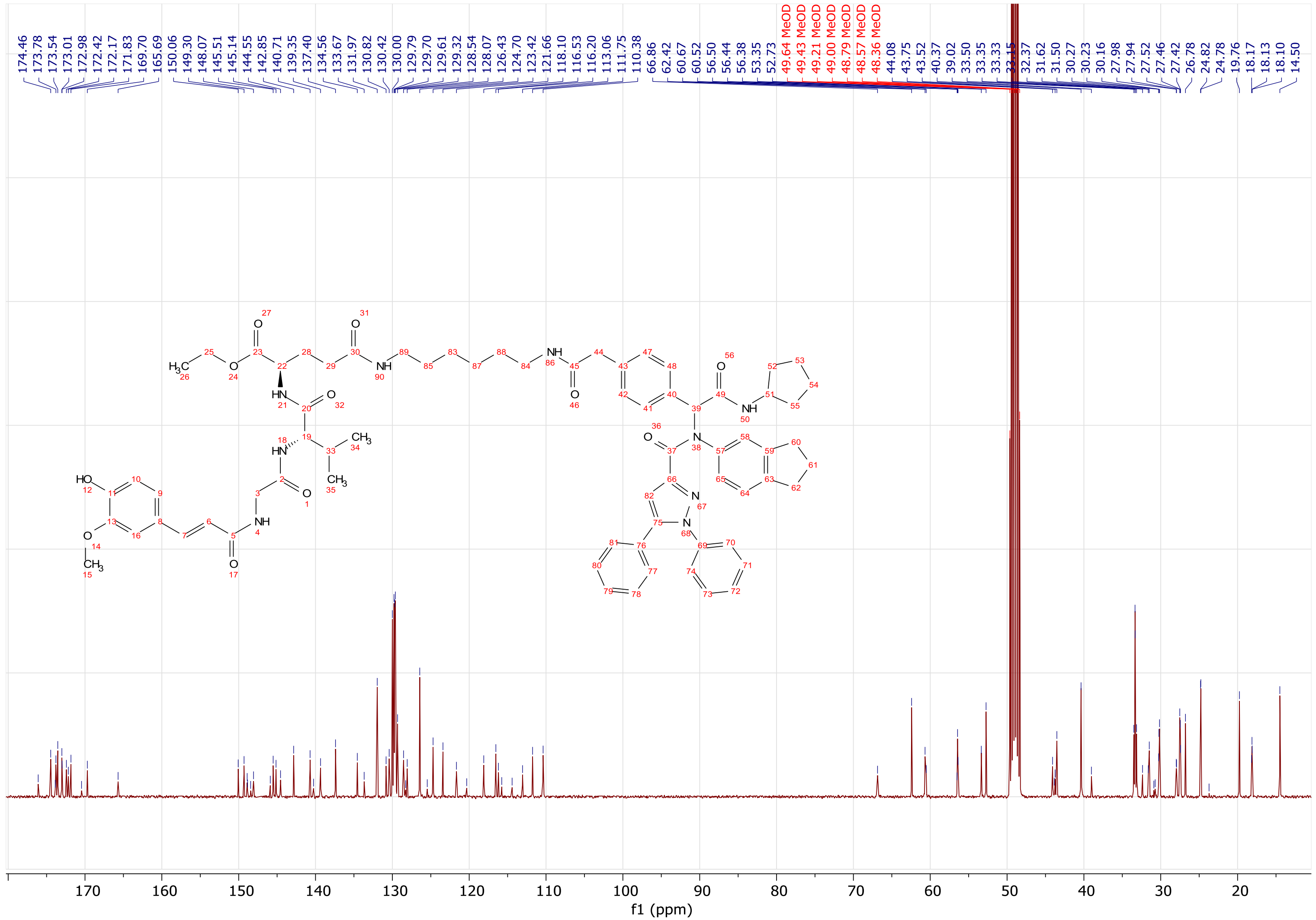

Figure S5. Compound **19**: ^{1}H, 400 MHz, MeOD

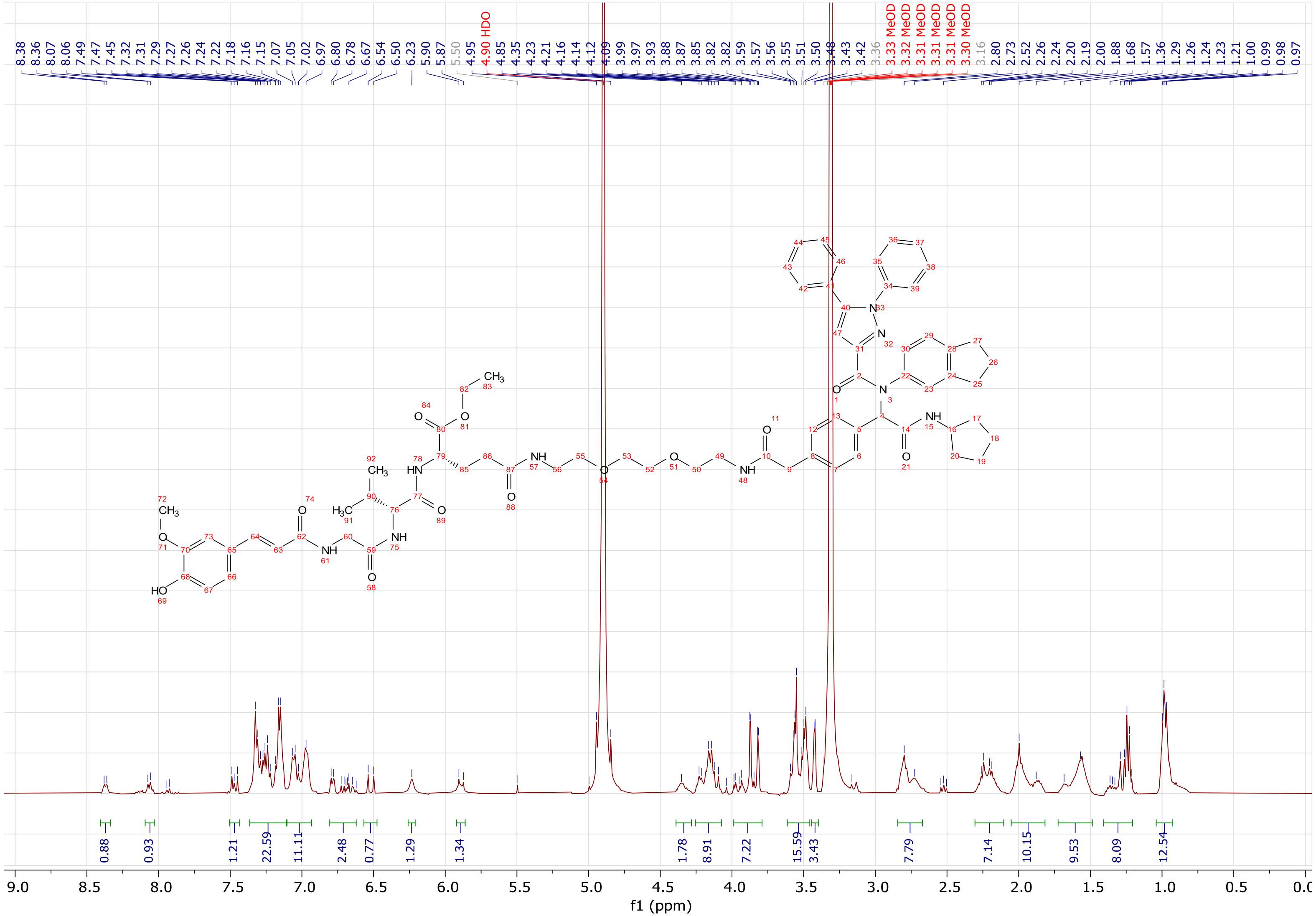

Figure S6. Compound **19**: ^{13}C, 400 MHz, MeOD

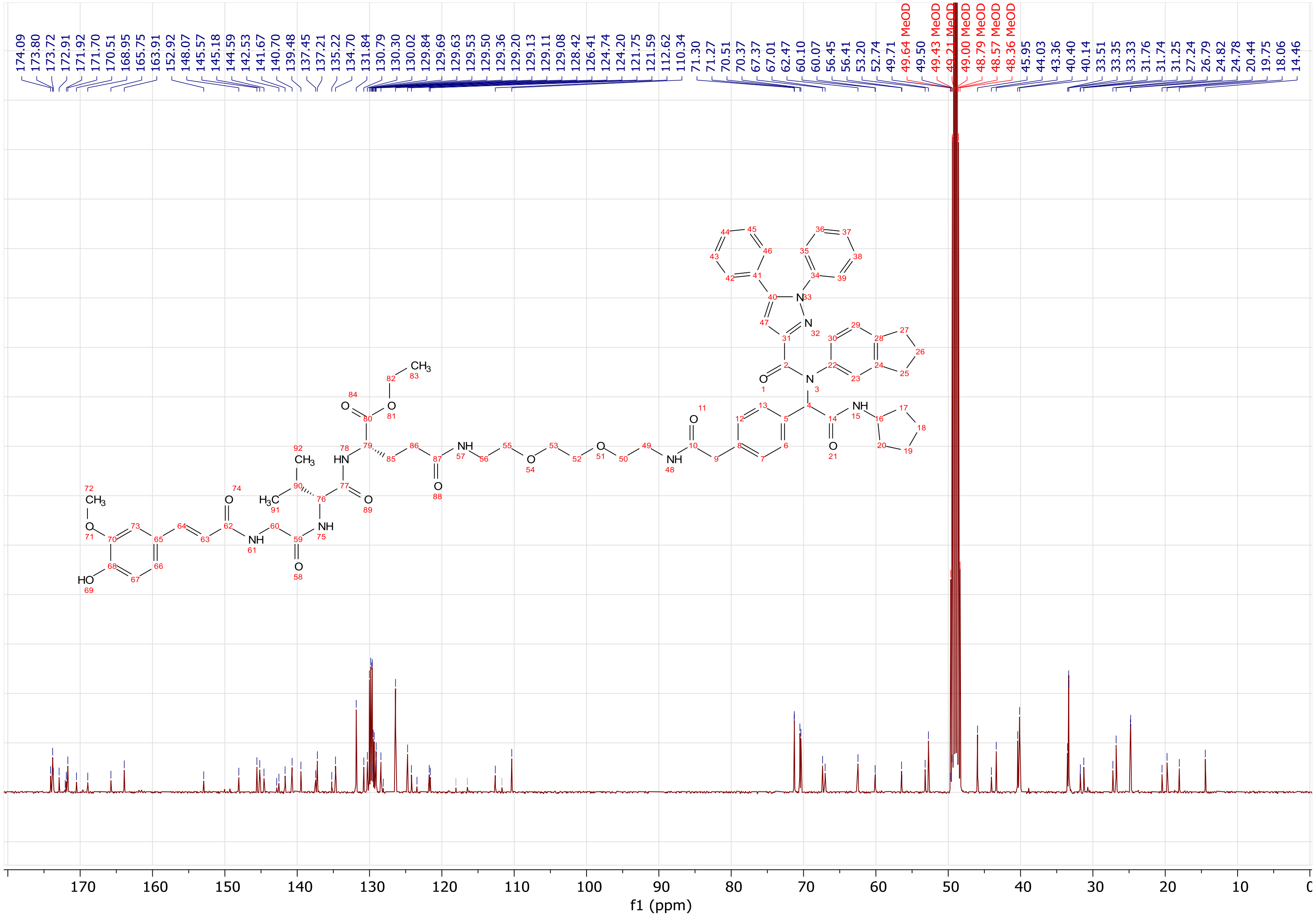

Figure S7. Compound **20**: ^{1}H, 400 MHz, MeOD

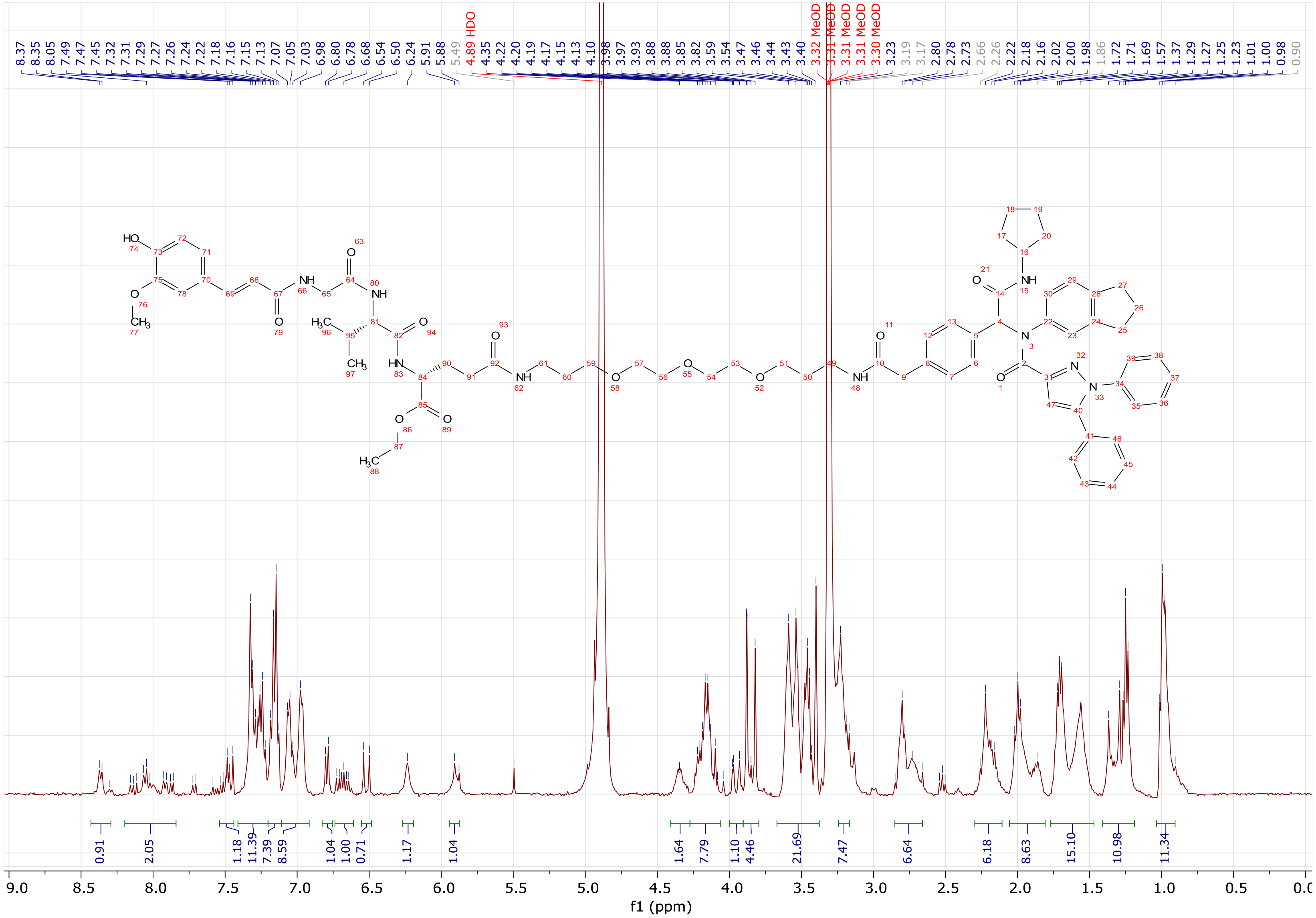

Figure S8. Compound **20**: ^{13}C, 400 MHz, MeOD

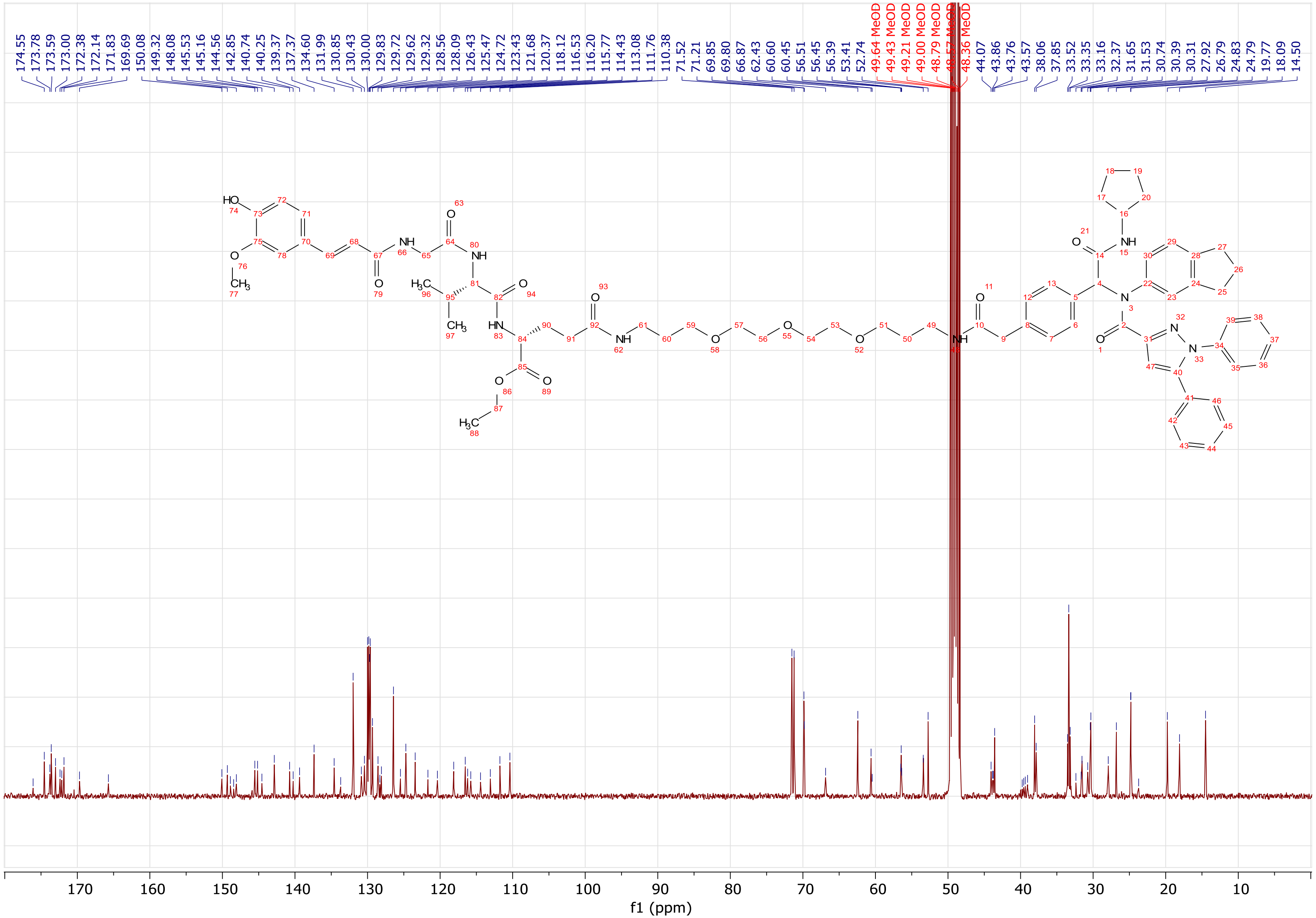

Figure S9. Compound **21**: ^{1}H, 400 MHz, MeOD

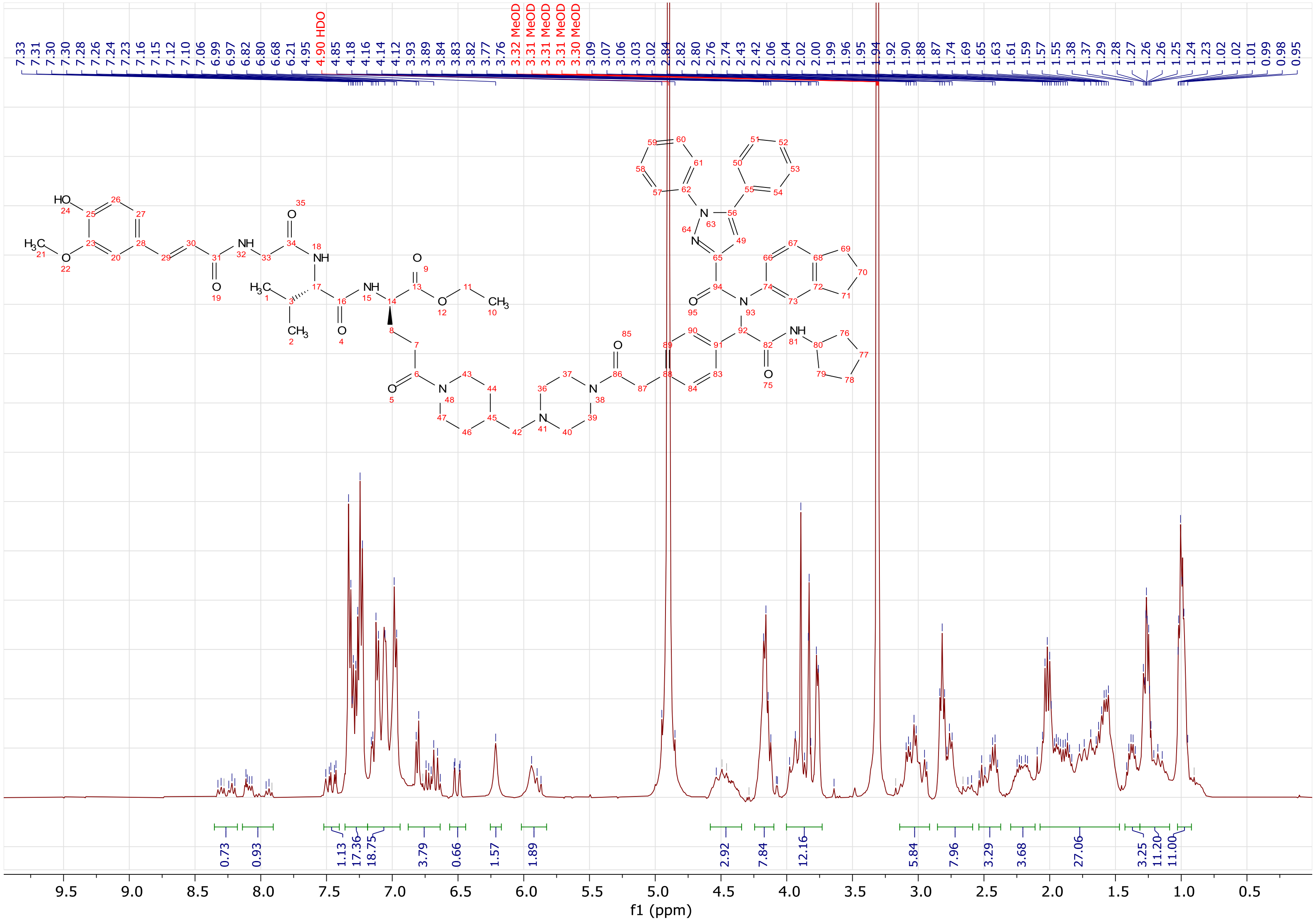

Figure S10. Compound **21**: ^{13}C, 400 MHz, MeOD

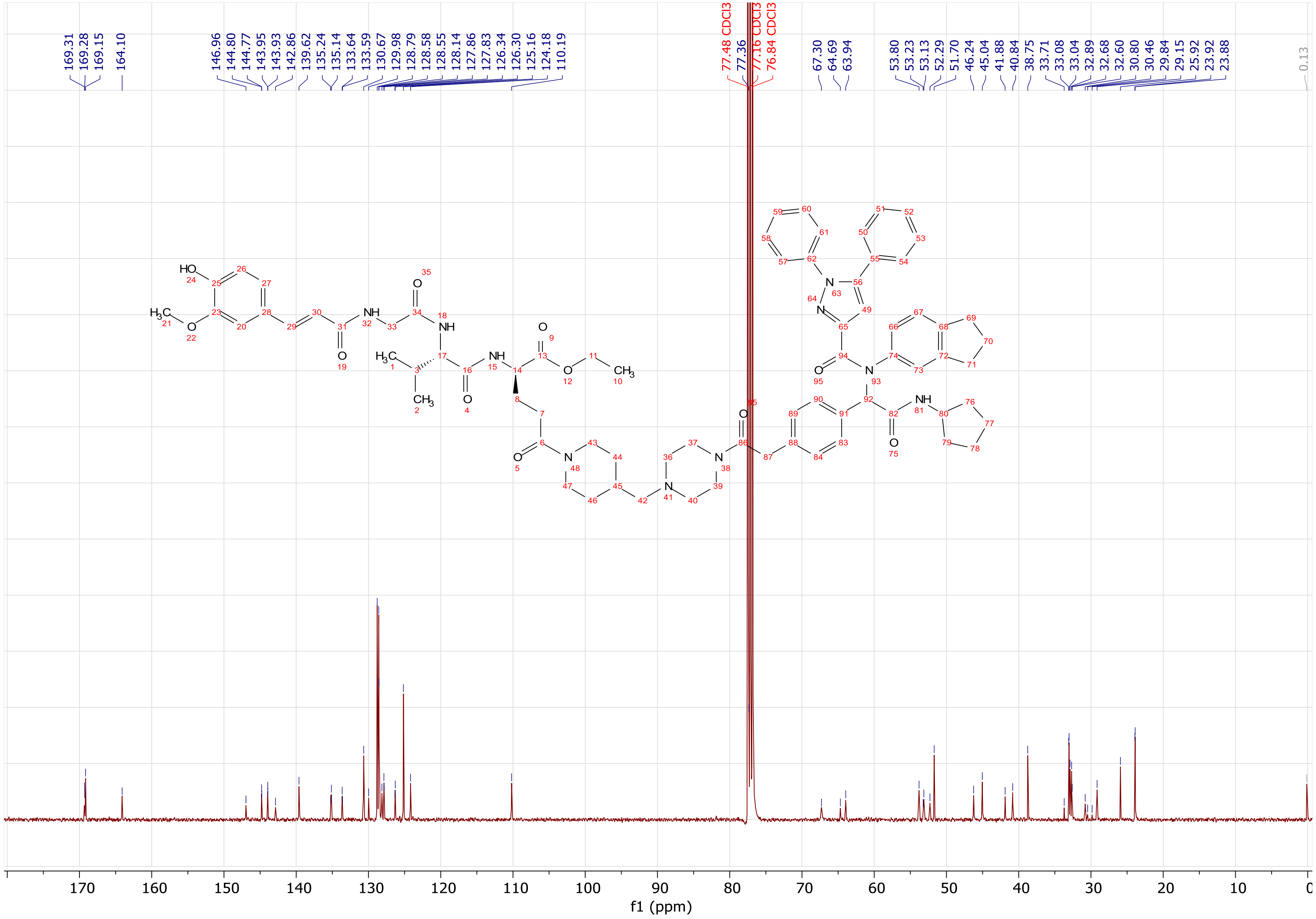

Figure S11. Compound **27**: ^{1}H, 400 MHz, MeOD

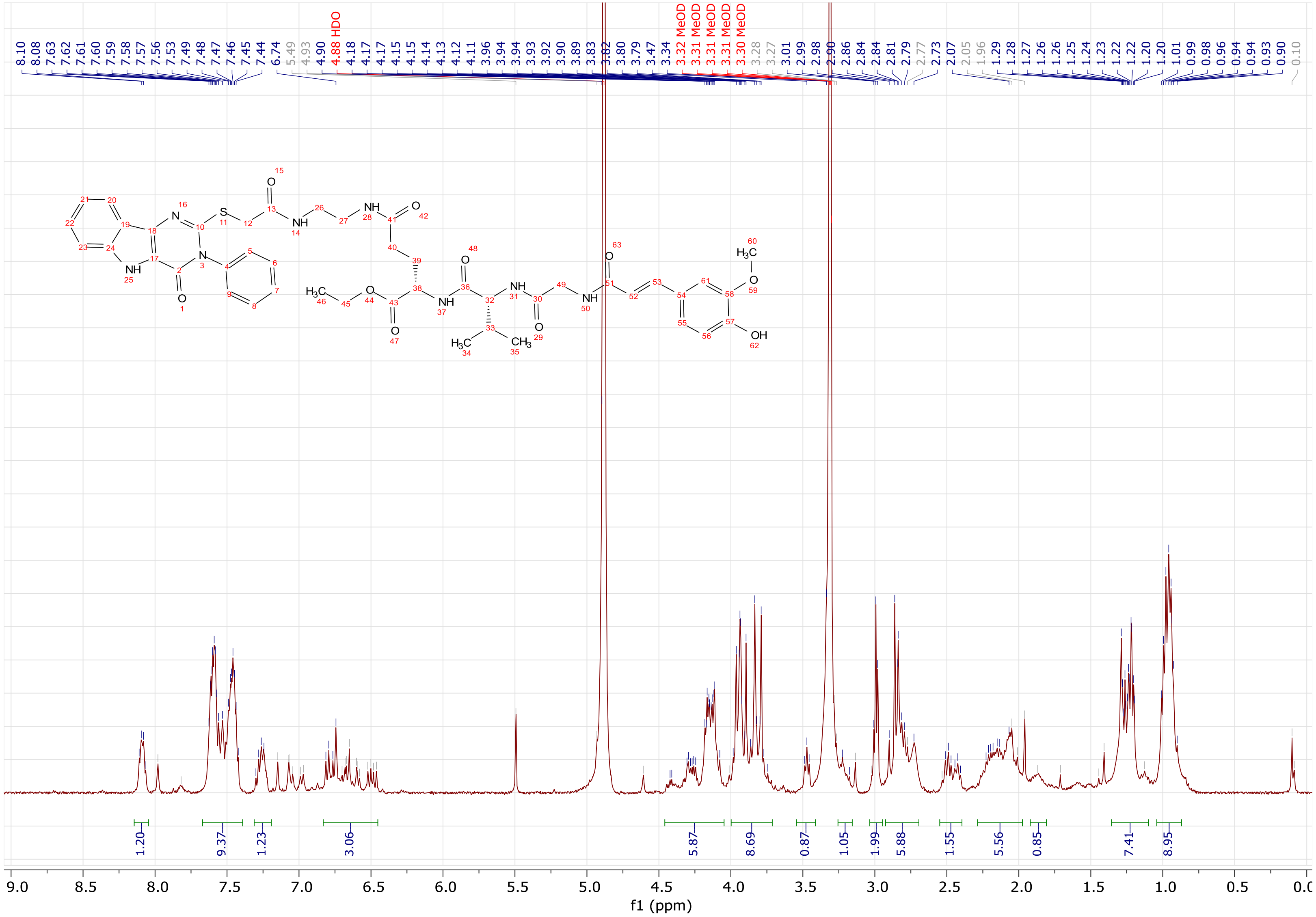

Figure S12. Compound **27**: ^{13}C, 400 MHz, DMSO

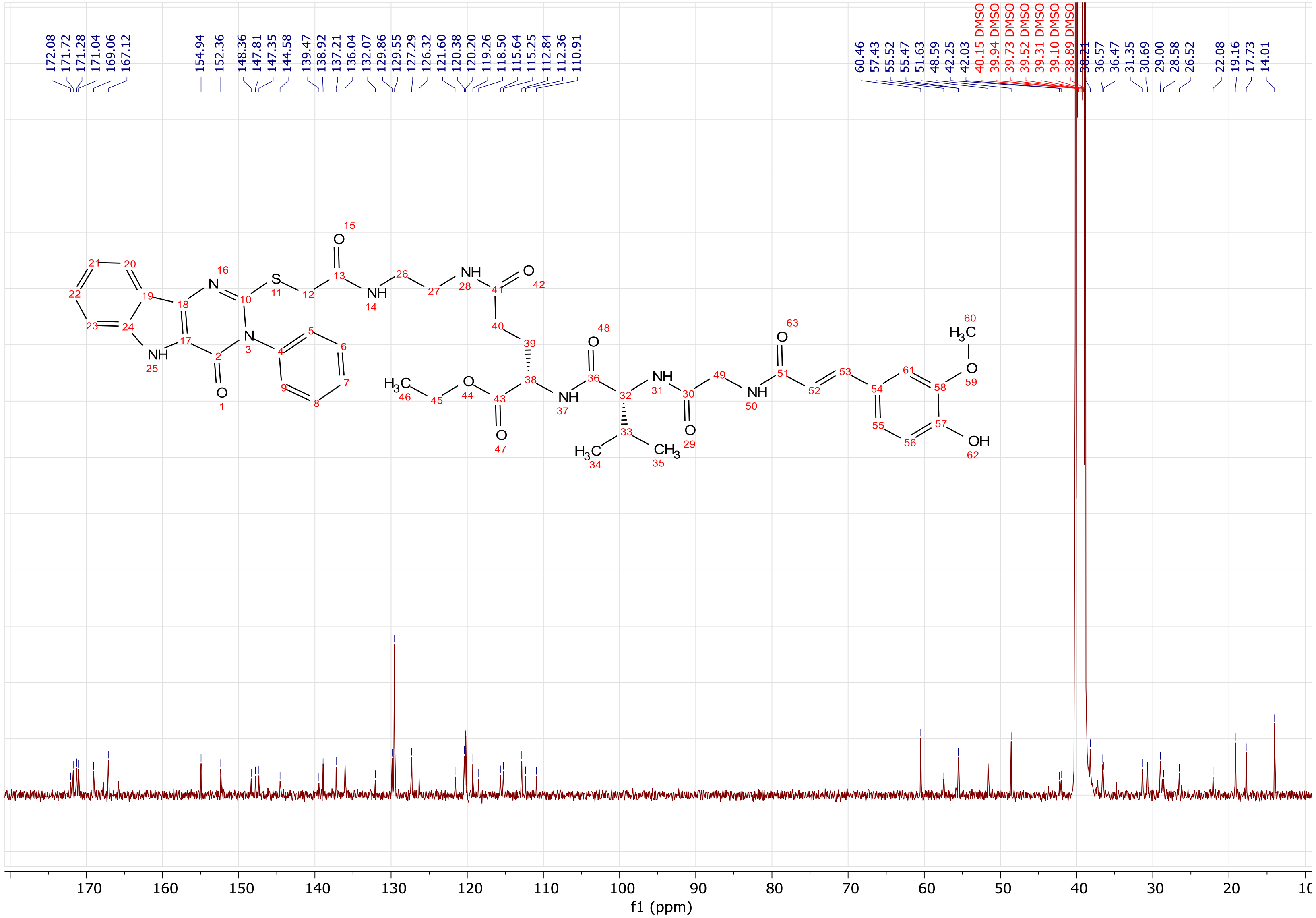

Figure S13. Compound **28**: ^{1}H, 400 MHz, MeOD

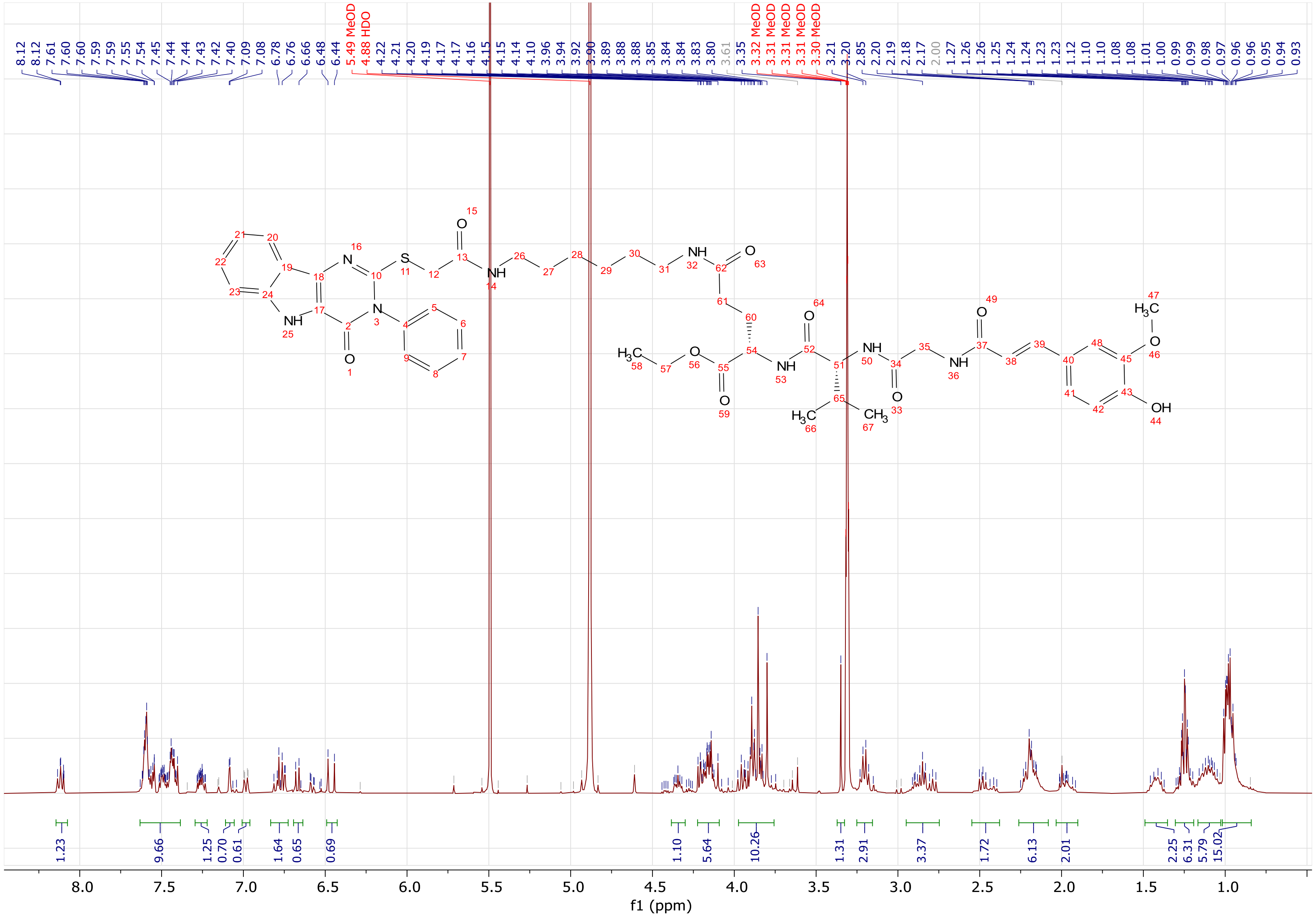

Figure S14. Compound **29**: ^{1}H, 400 MHz, MeOD

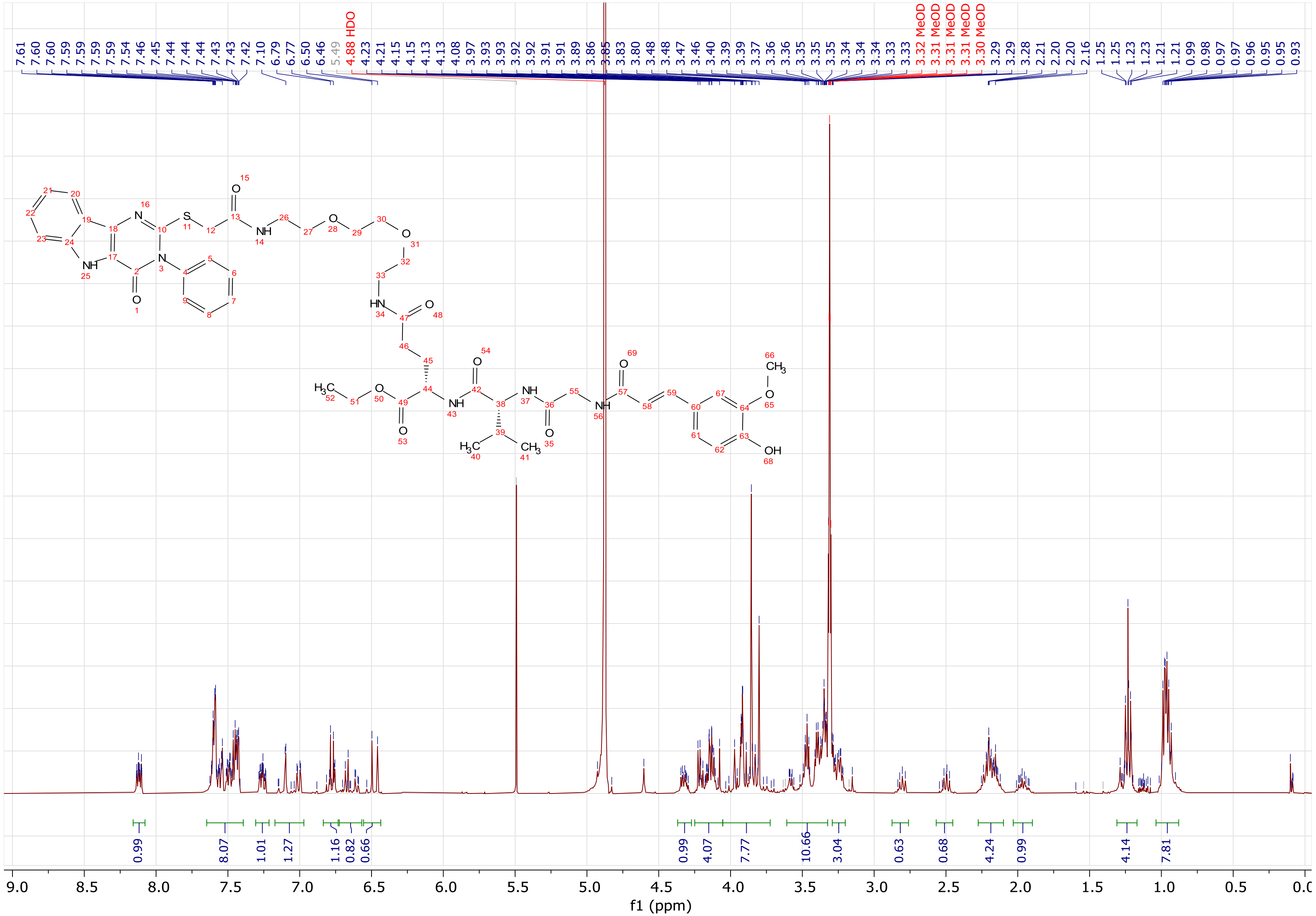

Figure S15. Compound **29**: ^{13}C, 400 MHz, MeOD

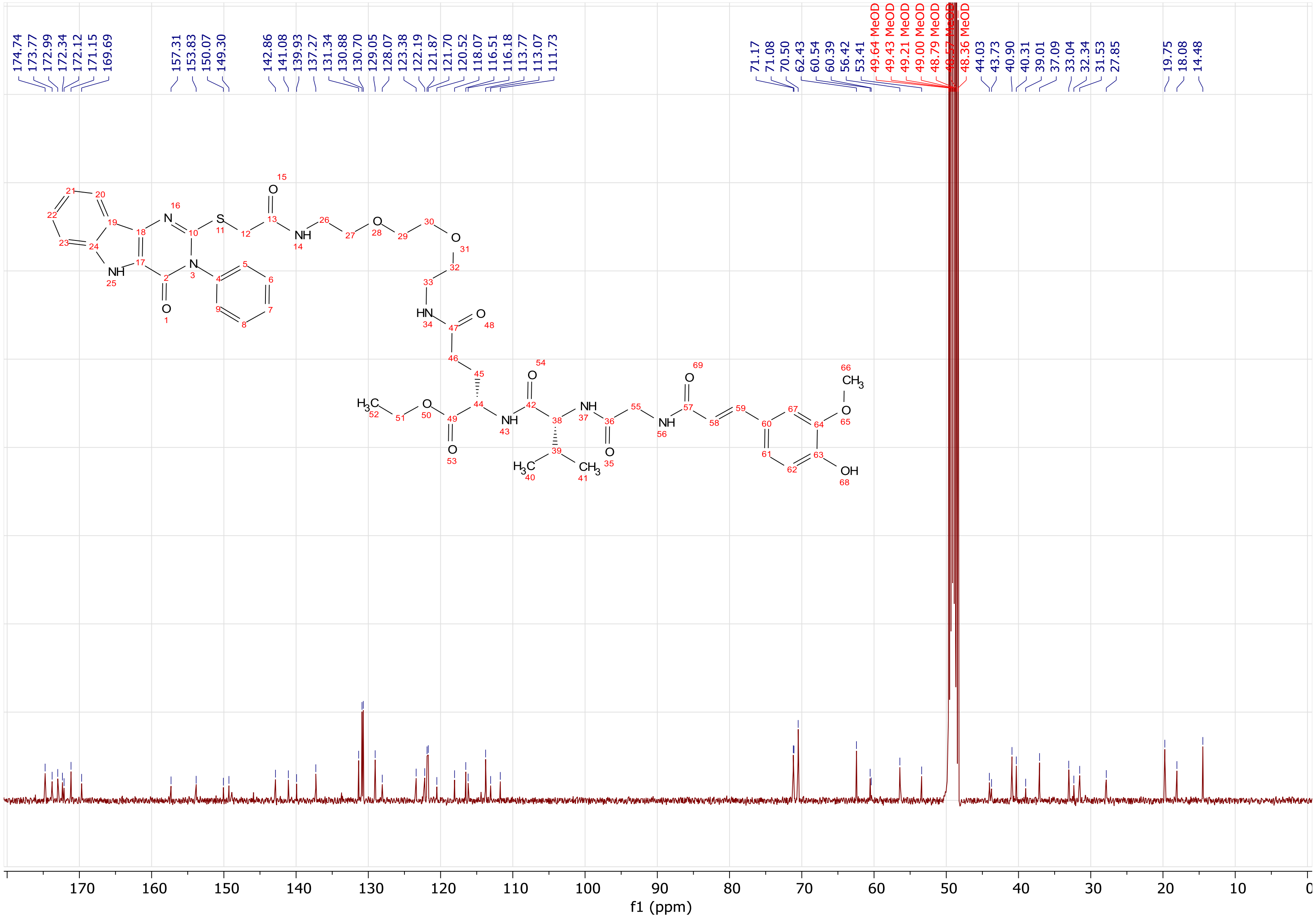

Figure S16. Compound **30**: ^{1}H, 400 MHz, MeOD

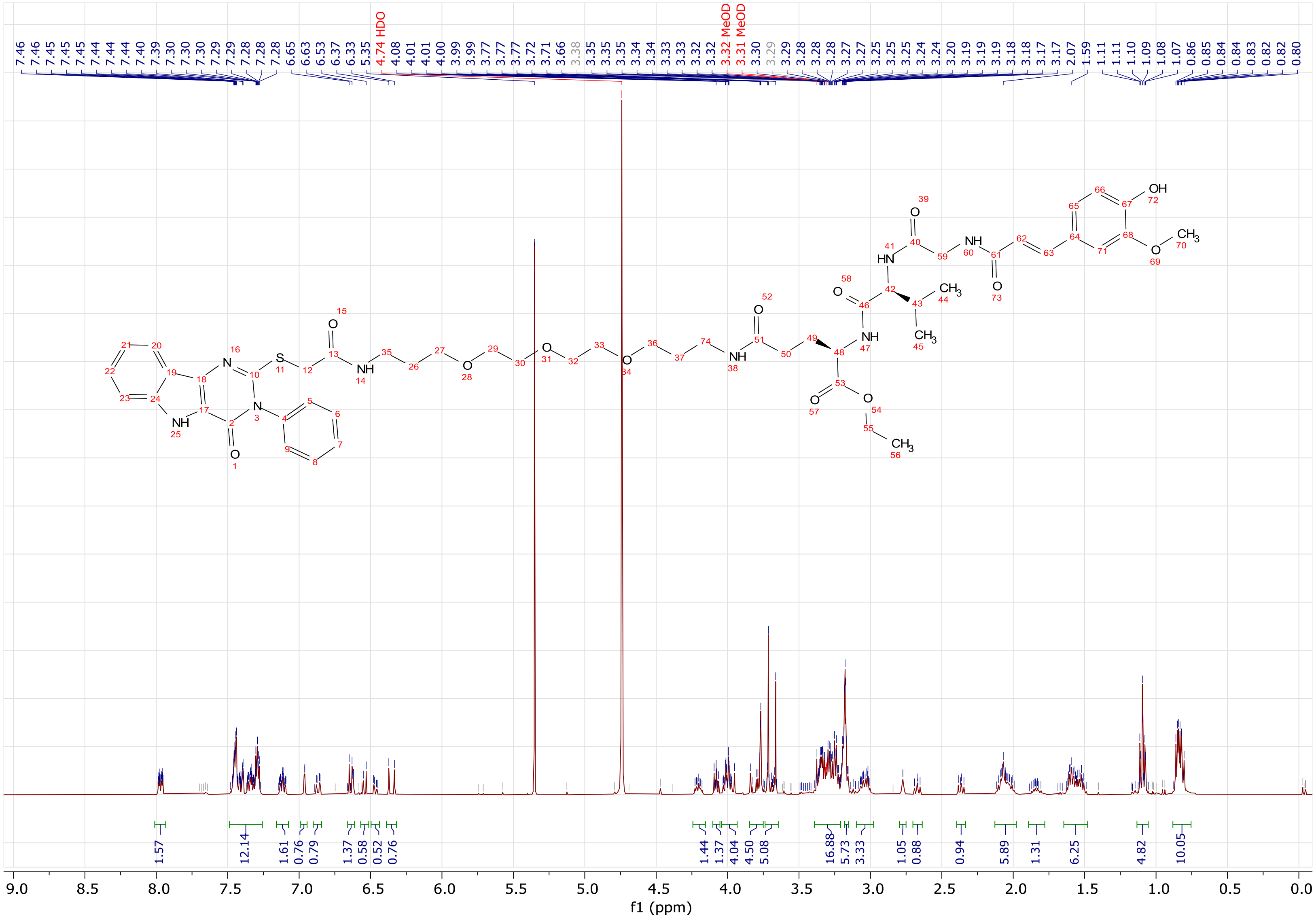

Figure S17. Compound **30**: ^{13}C, 400 MHz, MeOD

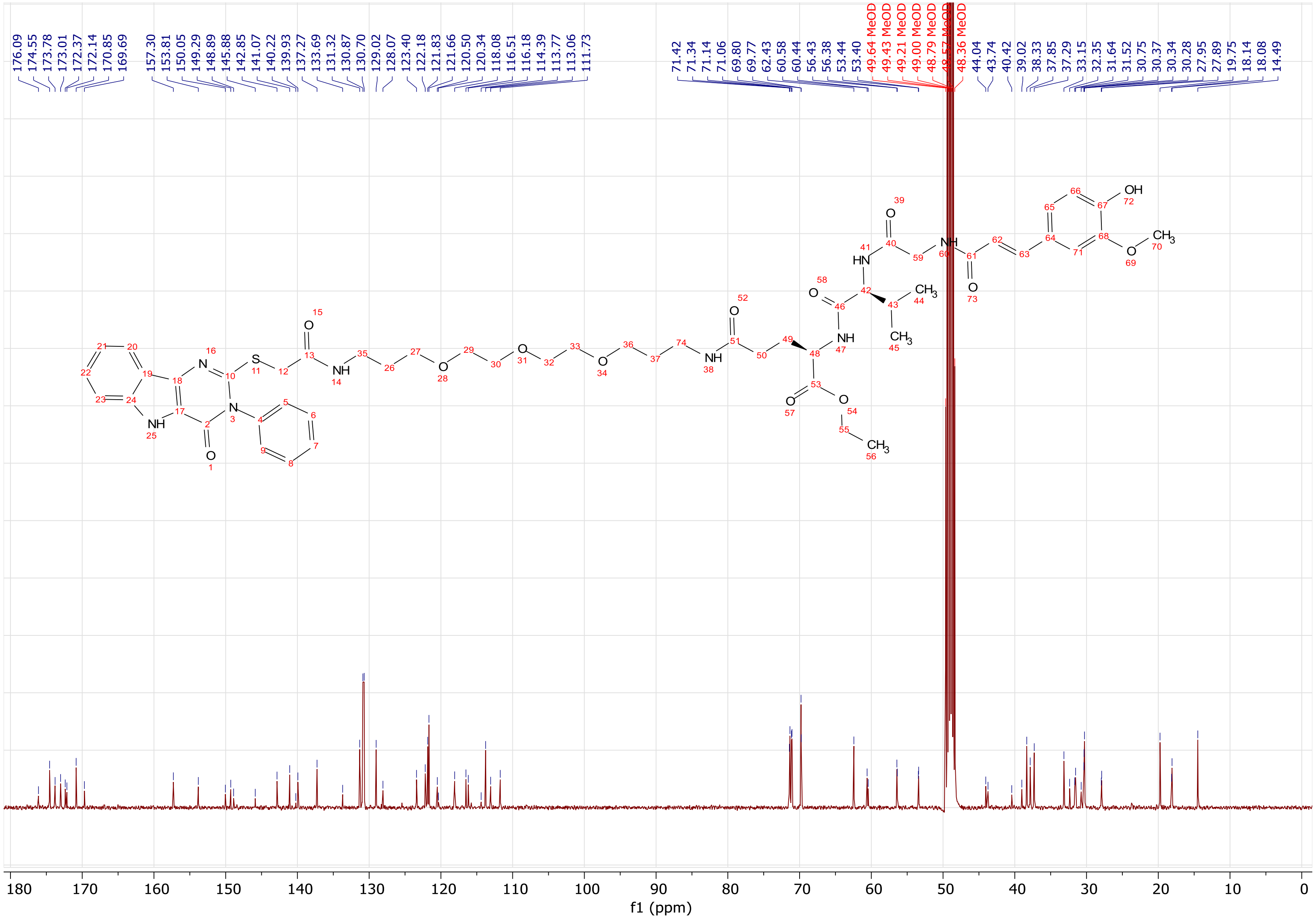

Figure S18. Compound **31**: ^{1}H, 400 MHz, MeOD

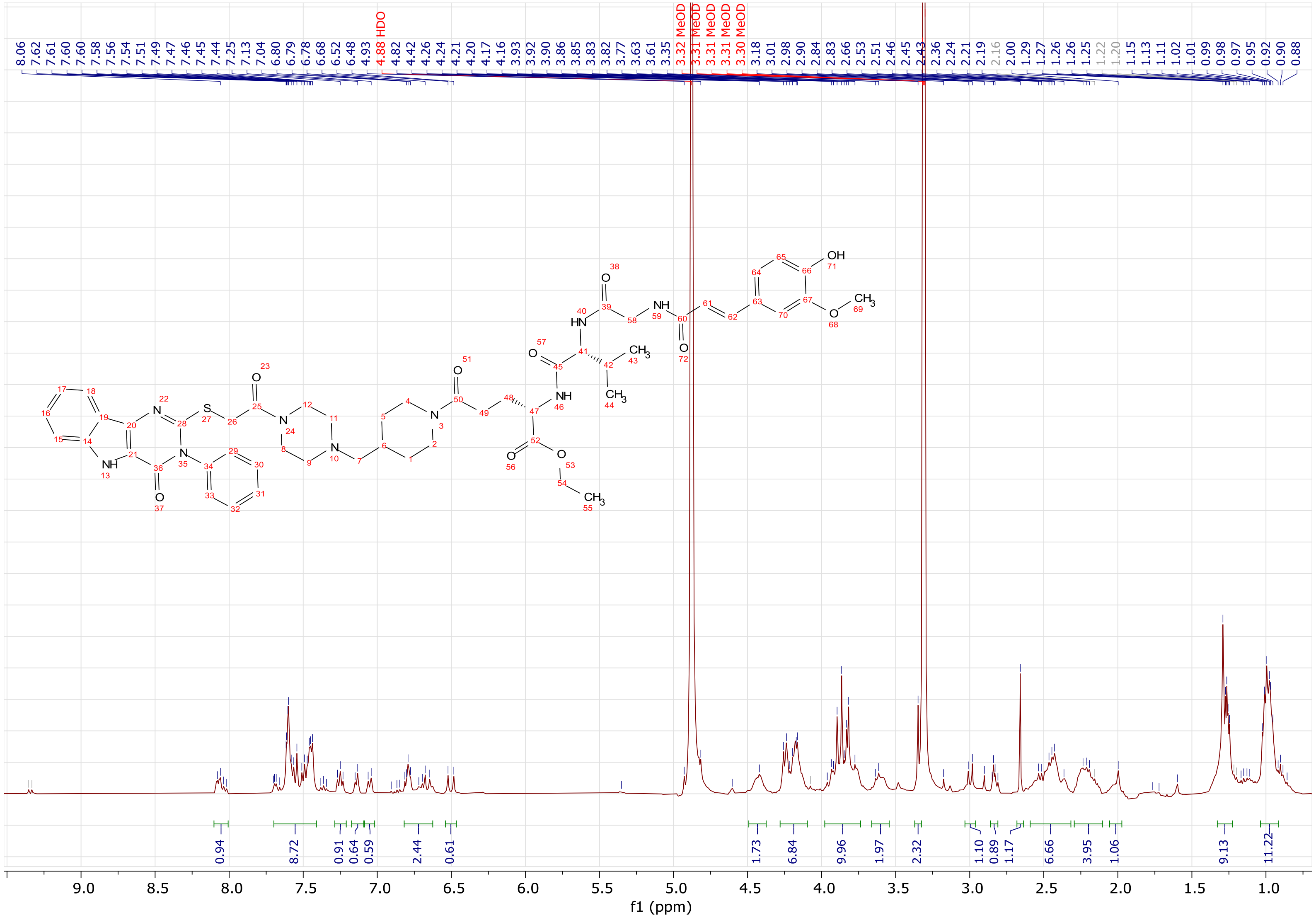

Figure S19. Compound **31**: ^{13}C, 400 MHz, DMSO

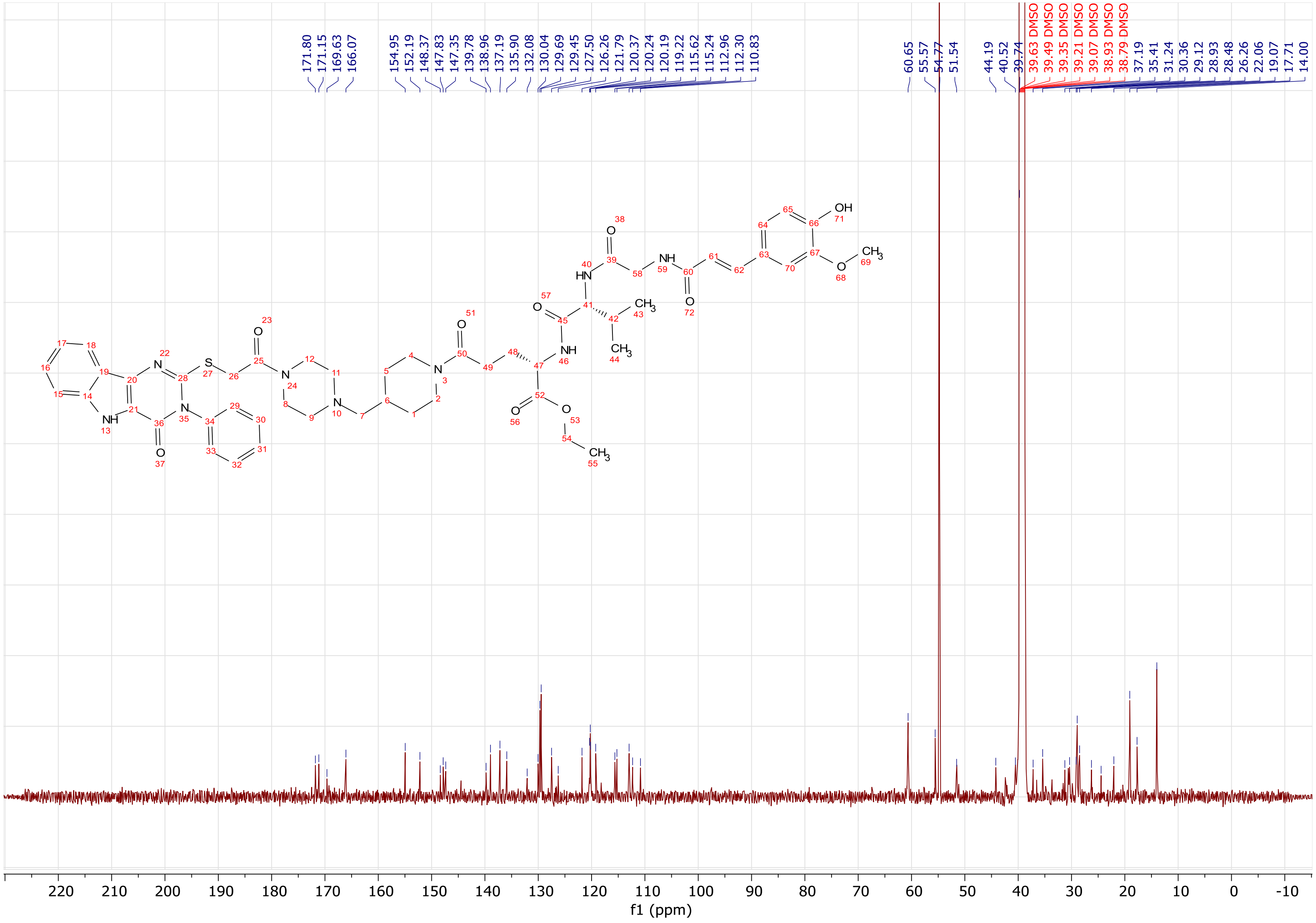

3. Representative UHPLC traces

Figure S20. Compound **17:**

Chromatogram and Results			
**Injection Details**			
Processing Method:	**Quantitative**	*Run Time (min):*	**12,50**
		Wavelength:	**254**

Chromatogram

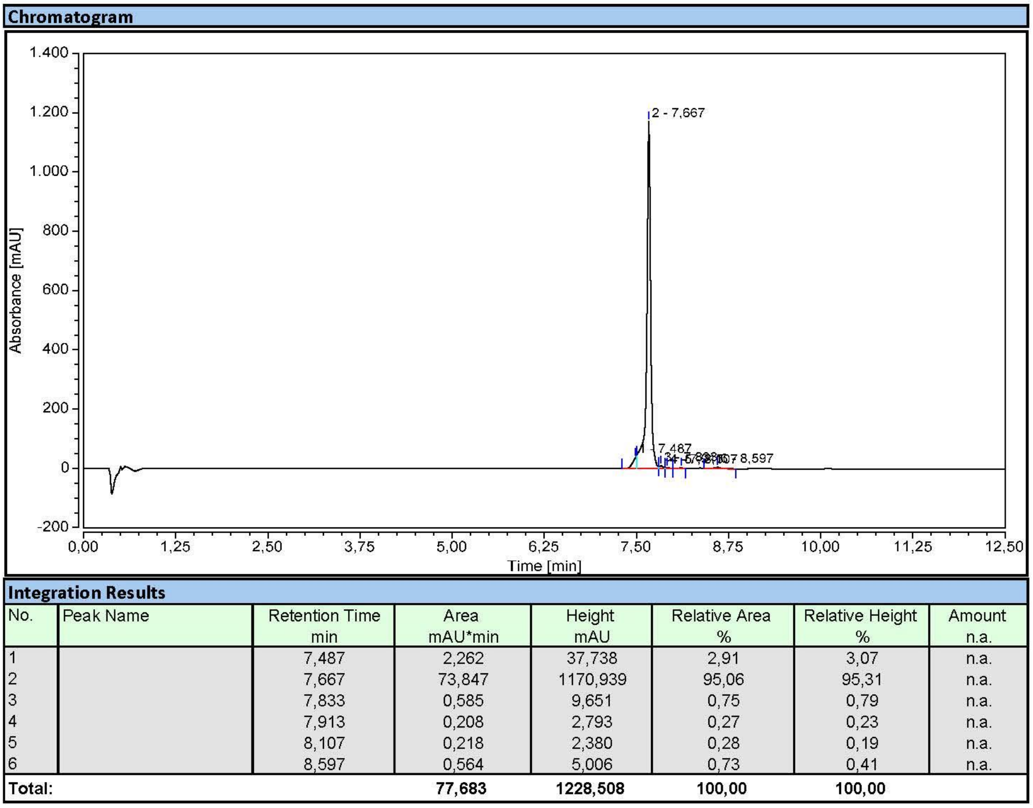

Integration Results

No.	Peak Name	Retention Time min	Area mAU*min	Height mAU	Relative Area %	Relative Height %	Amount n.a.
1		7,487	2,262	37,738	2,91	3,07	n.a.
2		7,667	73,847	1170,939	95,06	95,31	n.a.
3		7,833	0,585	9,651	0,75	0,79	n.a.
4		7,913	0,208	2,793	0,27	0,23	n.a.
5		8,107	0,218	2,380	0,28	0,19	n.a.
6		8,597	0,564	5,006	0,73	0,41	n.a.
**Total:**			**77,683**	**1228,508**	**100,00**	**100,00**	

Figure S21. Compound **18:**

Chromatogram and Results

Injection Details

Processing Method:	**Quantitative**	*Run Time (min):*	**12,50**
		Wavelength:	**254**

Chromatogram

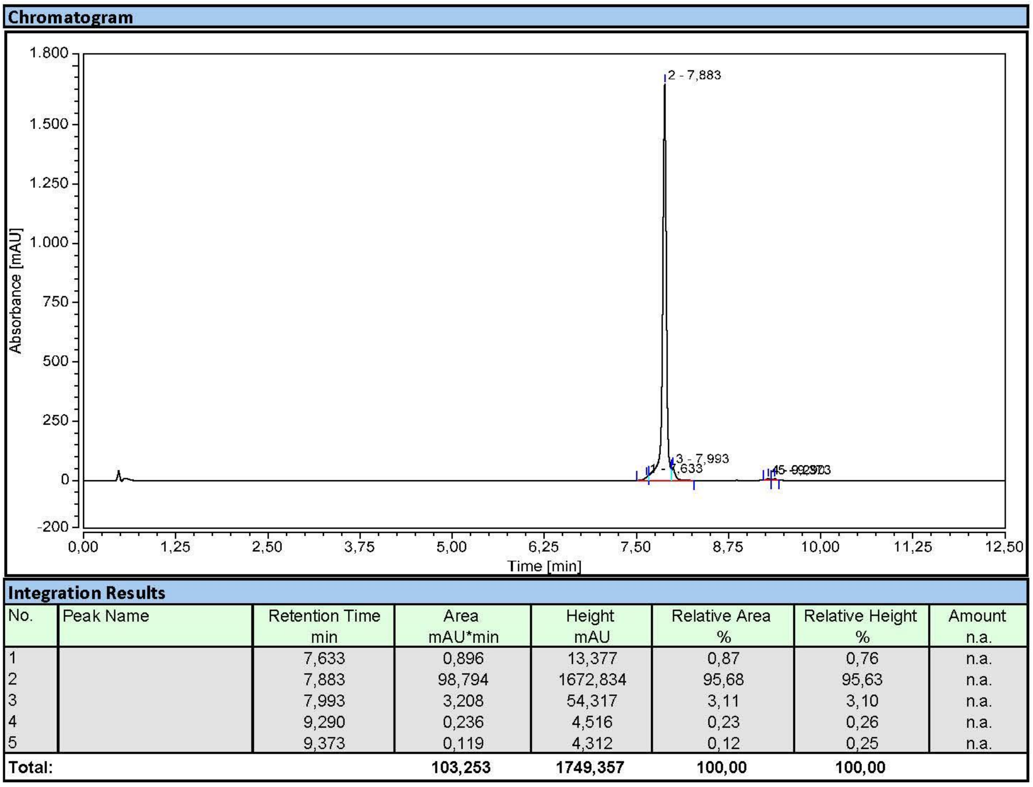

Integration Results

No.	Peak Name	Retention Time<br>min	Area<br>mAU*min	Height<br>mAU	Relative Area<br>%	Relative Height<br>%	Amount<br>n.a.
1		7,633	0,896	13,377	0,87	0,76	n.a.
2		7,883	98,794	1672,834	95,68	95,63	n.a.
3		7,993	3,208	54,317	3,11	3,10	n.a.
4		9,290	0,236	4,516	0,23	0,26	n.a.
5		9,373	0,119	4,312	0,12	0,25	n.a.
**Total:**			**103,253**	**1749,357**	**100,00**	**100,00**	

Figure S22. Compound **19:**

Chromatogram and Results

Injection Details

Processing Method:	**Quantitative**	*Run Time (min):*	**12,50**
		Wavelength:	**254**

Chromatogram

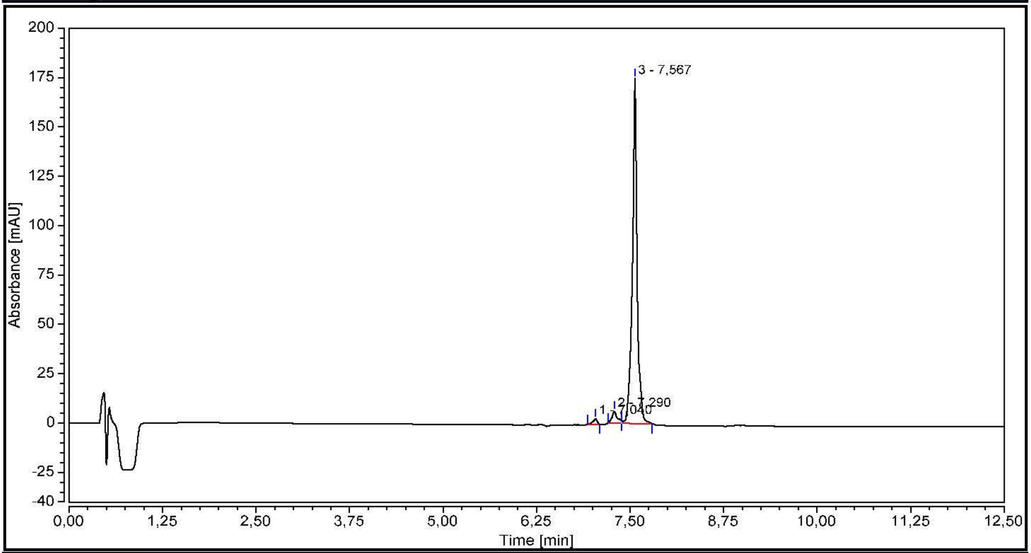

Integration Results

No.	Peak Name	Retention Time<br>min	Area<br>mAU*min	Height<br>mAU	Relative Area<br>%	Relative Height<br>%	Amount<br>n.a.
1		7,040	0,190	2,996	1,41	1,63	n.a.
2		7,290	0,485	6,070	3,58	3,30	n.a.
3		7,567	12,863	174,754	95,01	95,07	n.a.
**Total:**			**13,538**	**183,820**	**100,00**	**100,00**	

Figure S23. Compound **20:**

Chromatogram and Results

Injection Details

Processing Method:	**Quantitative**	*Run Time (min):*	**12,50**
		Wavelength:	**254**

Chromatogram

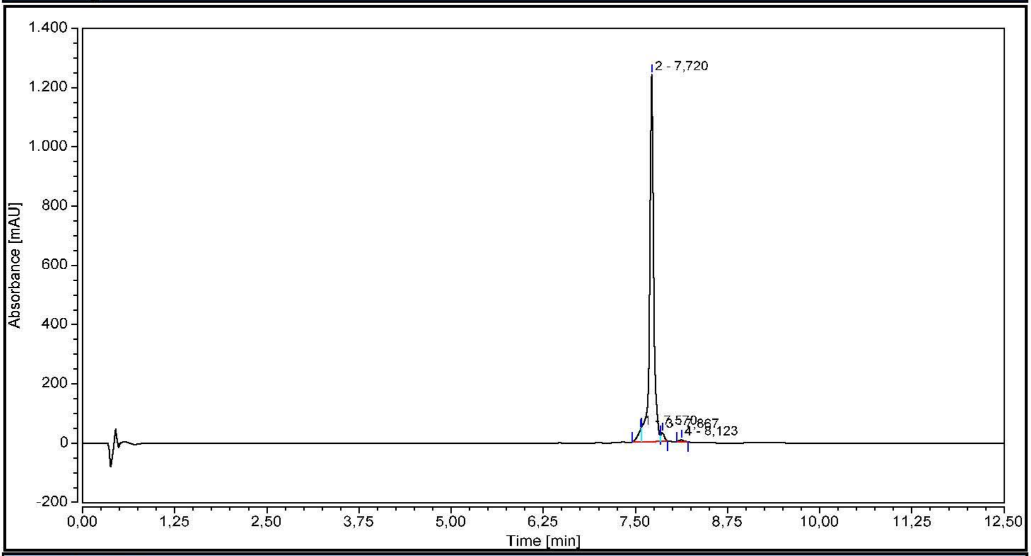

Integration Results

No.	Peak Name	Retention Time min	Area mAU*min	Height mAU	Relative Area %	Relative Height %	Amount n.a.
1		7,570	2,115	43,533	2,57	3,30	n.a.
2		7,720	78,179	1238,654	95,06	93,89	n.a.
3		7,867	1,528	30,550	1,86	2,32	n.a.
4		8,123	0,421	6,566	0,51	0,50	n.a.
**Total:**			**82,242**	**1319,303**	**100,00**	**100,00**	

Figure S24. Compound **21:**

Chromatogram and Results

Injection Details

Processing Method:	**Quantitative**	*Run Time (min):*	**12,50**
		Wavelength:	**254**

Chromatogram

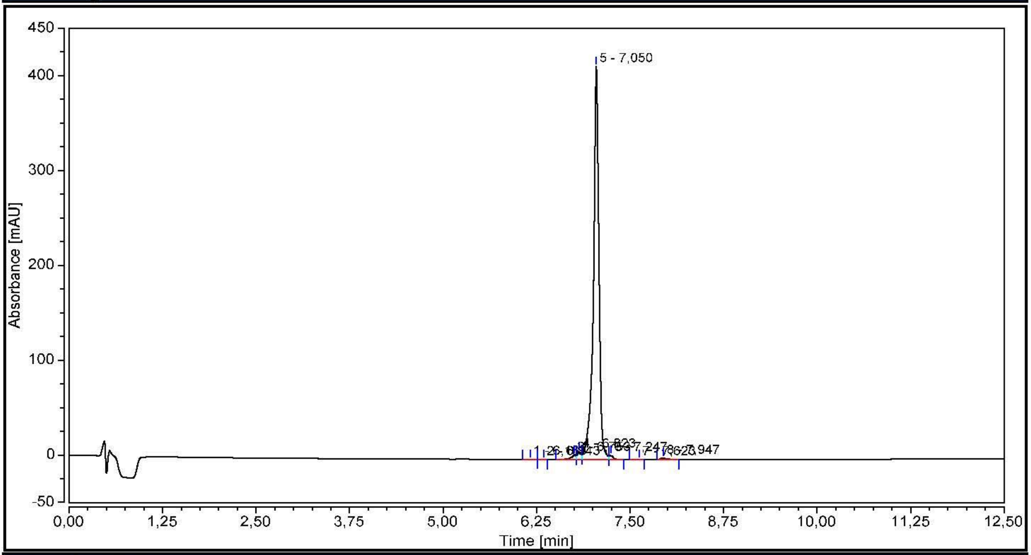

Integration Results

No.	Peak Name	Retention Time min	Area mAU*min	Height mAU	Relative Area %	Relative Height %	Amount n.a.
1		6,163	0,010	0,110	0,03	0,03	n.a.
2		6,343	0,013	0,155	0,03	0,04	n.a.
3		6,753	0,364	4,655	0,98	1,07	n.a.
4		6,823	0,478	7,989	1,29	1,84	n.a.
5		7,050	35,690	414,502	96,42	95,60	n.a.
6		7,247	0,277	4,471	0,75	1,03	n.a.
7		7,623	0,013	0,118	0,03	0,03	n.a.
8		7,947	0,171	1,591	0,46	0,37	n.a.
**Total:**			**37,015**	**433,591**	**100,00**	**100,00**	

Figure S25. Compound **27:**

Chromatogram and Results

Injection Details

Processing Method:	**Quantitative**	*Run Time (min):*	**12,50**
		Wavelength:	**254**

Chromatogram

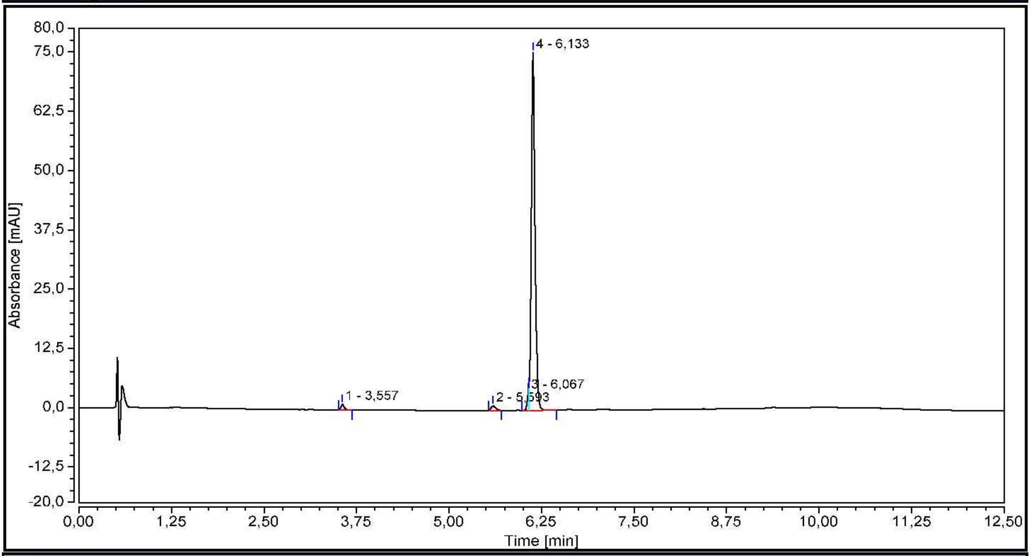

Integration Results

No.	Peak Name	Retention Time min	Area mAU*min	Height mAU	Relative Area %	Relative Height %	Amount n.a.
1		3,557	0,058	1,224	1,28	1,50	n.a.
2		5,593	0,070	0,975	1,52	1,20	n.a.
3		6,067	0,090	3,688	1,97	4,53	n.a.
4		6,133	4,345	75,455	95,23	92,76	n.a.
**Total:**			**4,563**	**81,343**	**100,00**	**100,00**	

Figure S26. Compound **28:**

Chromatogram and Results

Injection Details

Processing Method:	**Quantitative**	*Run Time (min):*	**12,50**
		Wavelength:	**254**

Chromatogram

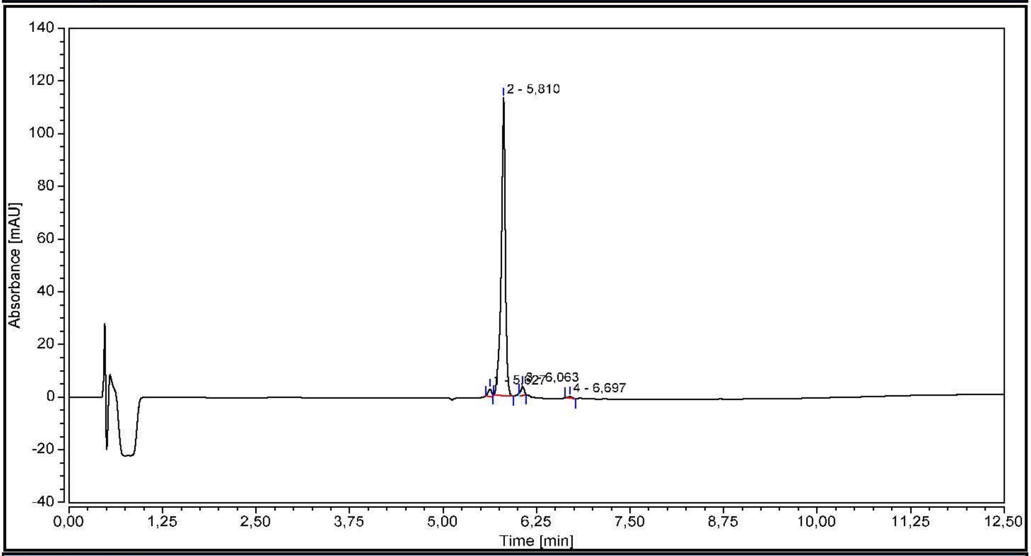

Integration Results

No.	Peak Name	Retention Time min	Area mAU*min	Height mAU	Relative Area %	Relative Height %	Amount n.a.
1		5,627	0,157	3,016	2,09	2,51	n.a.
2		5,810	7,122	113,194	95,08	94,06	n.a.
3		6,063	0,181	3,566	2,41	2,96	n.a.
4		6,697	0,031	0,562	0,42	0,47	n.a.
**Total:**			**7,490**	**120,338**	**100,00**	**100,00**	

Figure S27. Compound **29:**

Chromatogram and Results

Injection Details

Processing Method:	**Quantitative**	*Run Time (min):*	**12,50**
		Wavelength:	**254**

Chromatogram

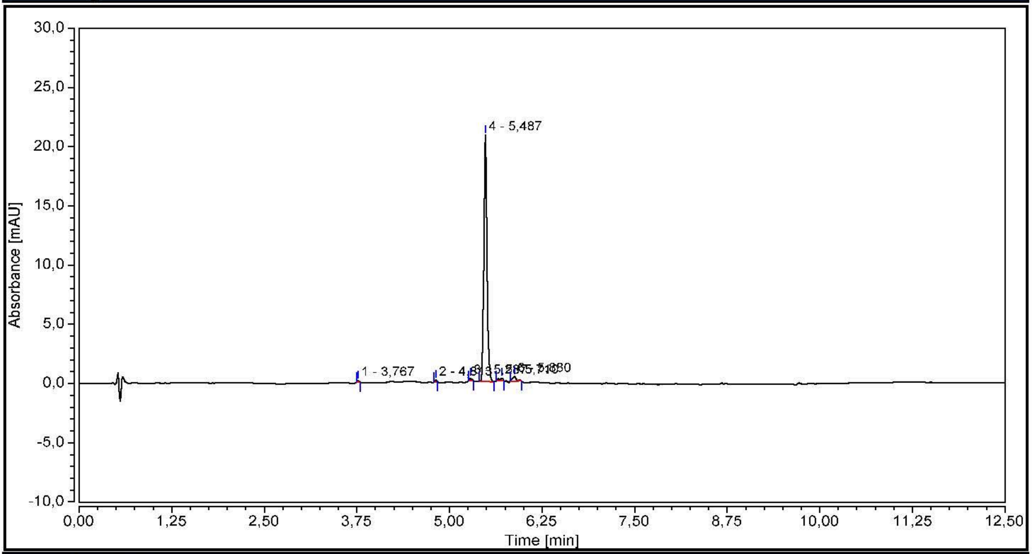

Integration Results

No.	Peak Name	Retention Time min	Area mAU*min	Height mAU	Relative Area %	Relative Height %	Amount n.a.
1		3,767	0,004	0,156	0,40	0,71	n.a.
2		4,813	0,005	0,172	0,44	0,78	n.a.
3		5,287	0,008	0,226	0,72	1,03	n.a.
4		5,487	1,014	20,839	95,01	94,51	n.a.
5		5,710	0,013	0,241	1,23	1,09	n.a.
6		5,880	0,024	0,415	2,20	1,88	n.a.
**Total:**			**1,068**	**22,049**	**100,00**	**100,00**	

Figure S28. Compound **30:**

Chromatogram and Results

Injection Details

Processing Method:	**Quantitative**	*Run Time (min):*	**12,50**
		Wavelength:	**254**

Chromatogram

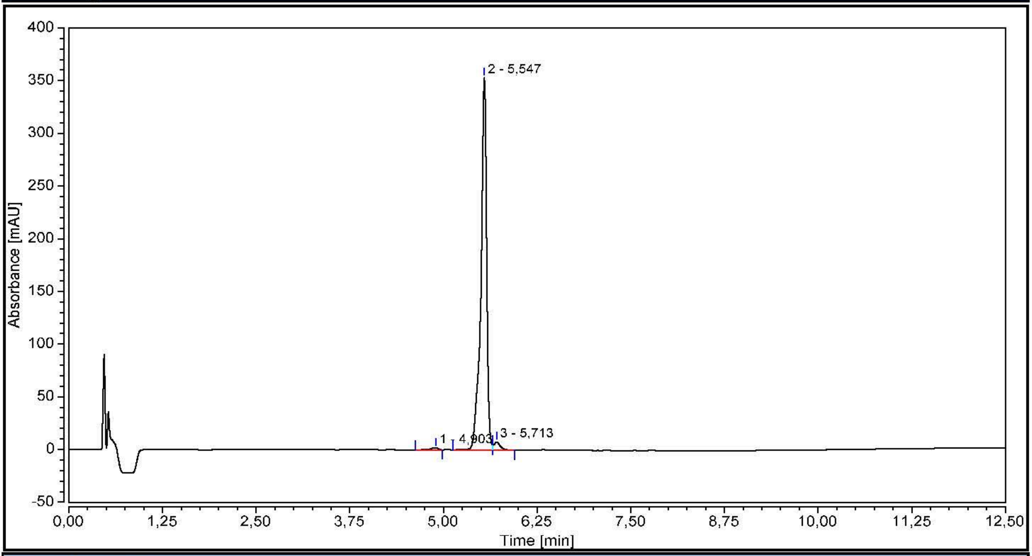

Integration Results

No.	Peak Name	Retention Time min	Area mAU*min	Height mAU	Relative Area %	Relative Height %	Amount n.a.
1		4,903	0,209	1,925	0,64	0,53	n.a.
2		5,547	31,631	353,376	97,04	97,27	n.a.
3		5,713	0,756	8,009	2,32	2,20	n.a.
**Total:**			**32,596**	**363,310**	**100,00**	**100,00**	

Figure S29. Compound **31:**

Chromatogram and Results

Injection Details

Processing Method:	**Quantitative**	*Run Time (min):*	**12,50**
		Wavelength:	**254**

Chromatogram

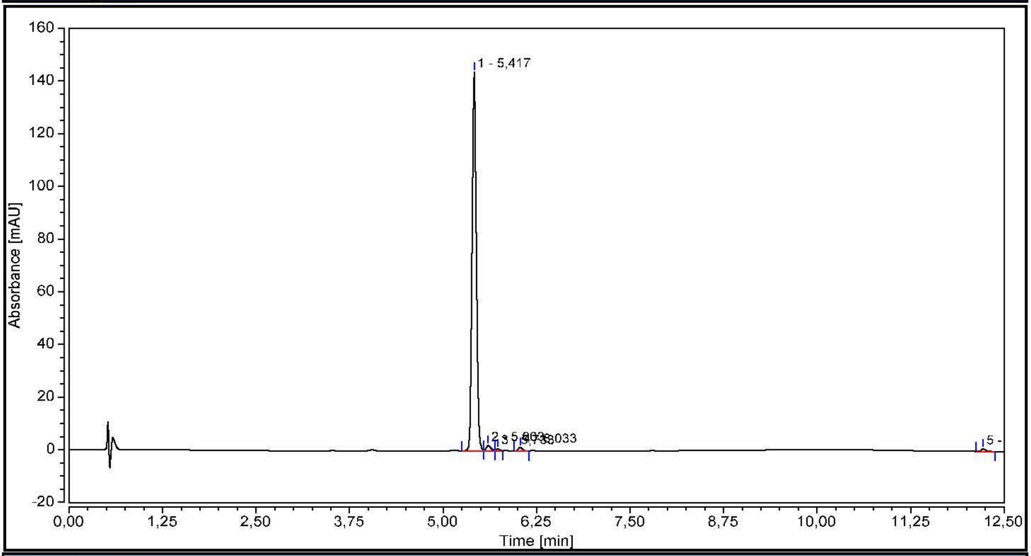

Integration Results

No.	Peak Name	Retention Time min	Area mAU*min	Height mAU	Relative Area %	Relative Height %	Amount n.a.
1		5,417	9,336	143,918	96,34	96,39	n.a.
2		5,603	0,152	2,138	1,57	1,43	n.a.
3		5,733	0,044	0,822	0,45	0,55	n.a.
4		6,033	0,089	1,434	0,92	0,96	n.a.
5		12,220	0,069	0,990	0,72	0,66	n.a.
**Total:**			**9,690**	**149,302**	**100,00**	**100,00**	

4. Solubility data

Table S1.

Compounds		2	4	6
Retention time (sample K1)		*5.63*	*5.67*	*3.913*
**Sample**	Concentration (µM)	Peak Area		
**K5**	0.8	0.05	0.42	0.26
**K4**	4	0.29	1.55	1.10
**K3**	20	1.24	6.28	5.27
**K2**	100	5.48	30.32	21.41
**K1**	500	25.95	114.80	93.38
**Sample 1**		0.01	25.10	22.22
**Sample 2**		0.01	22.84	19.61
**R^2**		1.00	1.00	1.00
**m**		0.05	0.23	0.19
**Solubility sample 1**		**0.26**	**248.79**	**269.89**
**Solubility sample 2**		**0.44**	**226.38**	**238.29**
**Average solubility (µM)**		**0.35**	**237.58**	**254.09**

Table S2.

Compounds		17	18	19	20	21
Retention time (sample K1)		*5.347*	*5.467*	*5.3*	*5.36*	*4.947*
**Sample**	Concentration (µM)	Peak Area				
**K5**	0.8	0.46	0.10	0.16	0.22	1.09
**K4**	4	1.85	0.67	0.51	0.61	4.97
**K3**	20	8.29	2.74	2.18	2.44	18.72
**K2**	100	35.55	12.62	8.53	10.62	76.45
**K1**	500	120.53	43.03	33.91	44.34	267.09
**Sample 1**		0.07	0.03	0.08	0.06	0.08
**Sample 2**		0.09	0.04	0.05	0.05	0.07
**R^2**		0.99	0.99	1.00	1.00	0.99
**m**		0.24	0.09	0.07	0.09	0.52
**Solubility sample 1**		**0.68**	**0.85**	**2.59**	**1.61**	**0.34**
**Solubility sample 2**		**0.83**	**1.17**	**1.68**	**1.38**	**0.32**
**Average solubility (µM)**		**0.76**	**1.01**	**2.14**	**1.50**	**0.33**

Table S3.

Compounds		27	28	29	30	31
Retention time (sample K1)		*3.933*	*4.237*	*4.01*	*4.107*	*3.717*
**Sample**	Concentration (µM)	Peak Area				
**K5**	0.8	0.03	0.03	0.07	0.14	0.07
**K4**	4	0.10	0.12	0.29	0.55	0.28
**K3**	20	0.39	0.69	1.25	2.38	1.55
**K2**	100	1.92	2.65	5.78	11.43	8.27
**K1**	500	8.93	15.24	27.78	52.61	28.69
**Sample 1**		2.54	1.56	5.70	5.77	0.18
**Sample 2**		2.36	1.09	6.01	5.63	0.16
**R^2**		1.00	1.00	1.00	1.00	0.99
**m**		0.02	0.03	0.06	0.11	0.06
**Solubility sample 1**		**321.63**	**115.27**	**231.70**	**123.92**	**7.22**
**Solubility sample 2**		**298.48**	**80.73**	**244.18**	**120.96**	**6.15**
**Average solubility (µM)**		**310.05**	**79.99**	**237.94**	**122.44**	**6.68**

5. High-Resolution Mass Spectra (HRMS)

Figure S30. Compound **17**

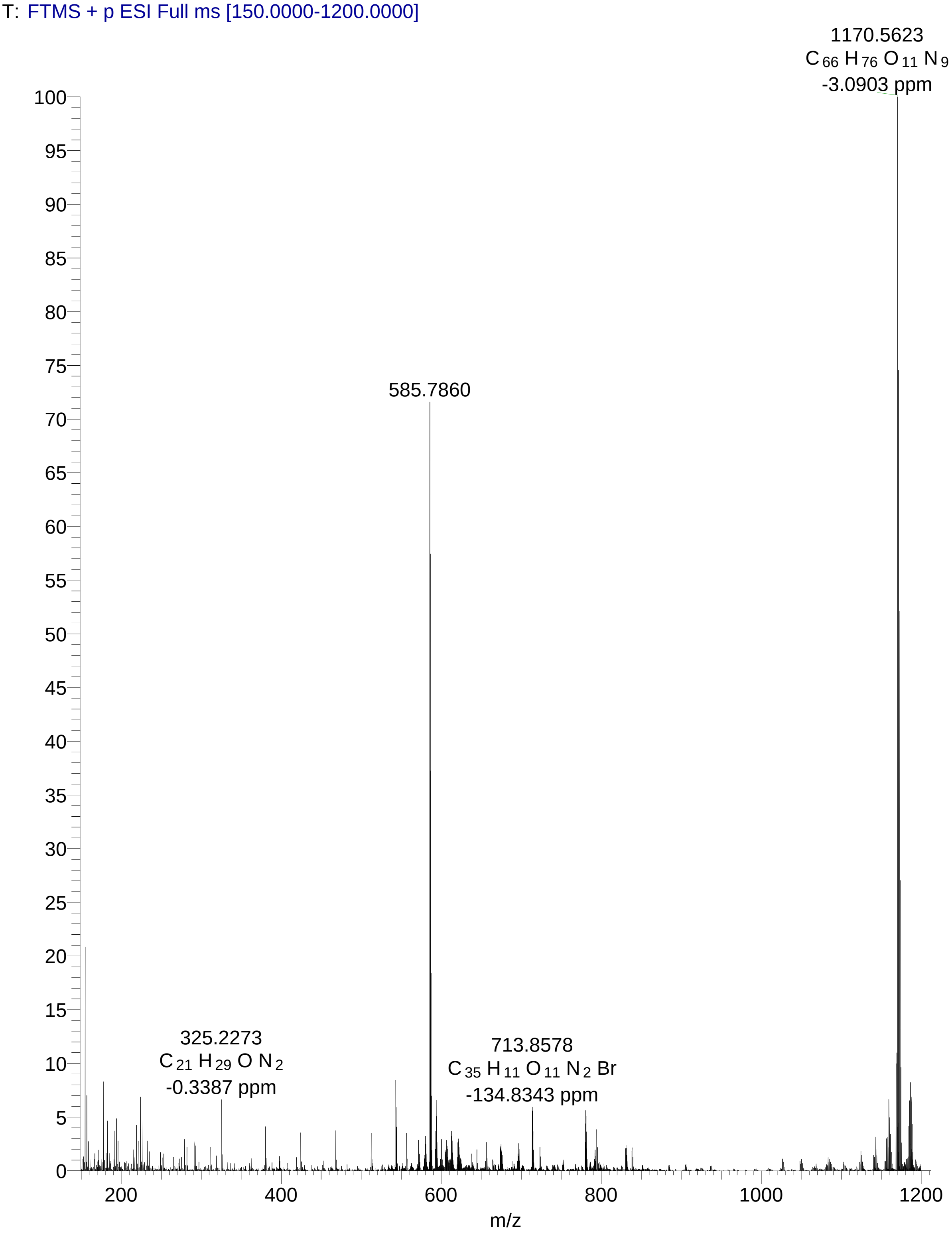

Elemental composition search on mass 1170.56226

m/z= 1165.56226-1175.56226

m/z	Theo. Mass	Delta (mmu)	RDB equiv.	Composition
1170.5623	1170.5659	-3.62	33.5	$C_{66}H_{76}O_{11}N_{9}$

Figure S31. Compound **18**

T: FTMS + c ESI Full ms [100.0000-1300.0000]

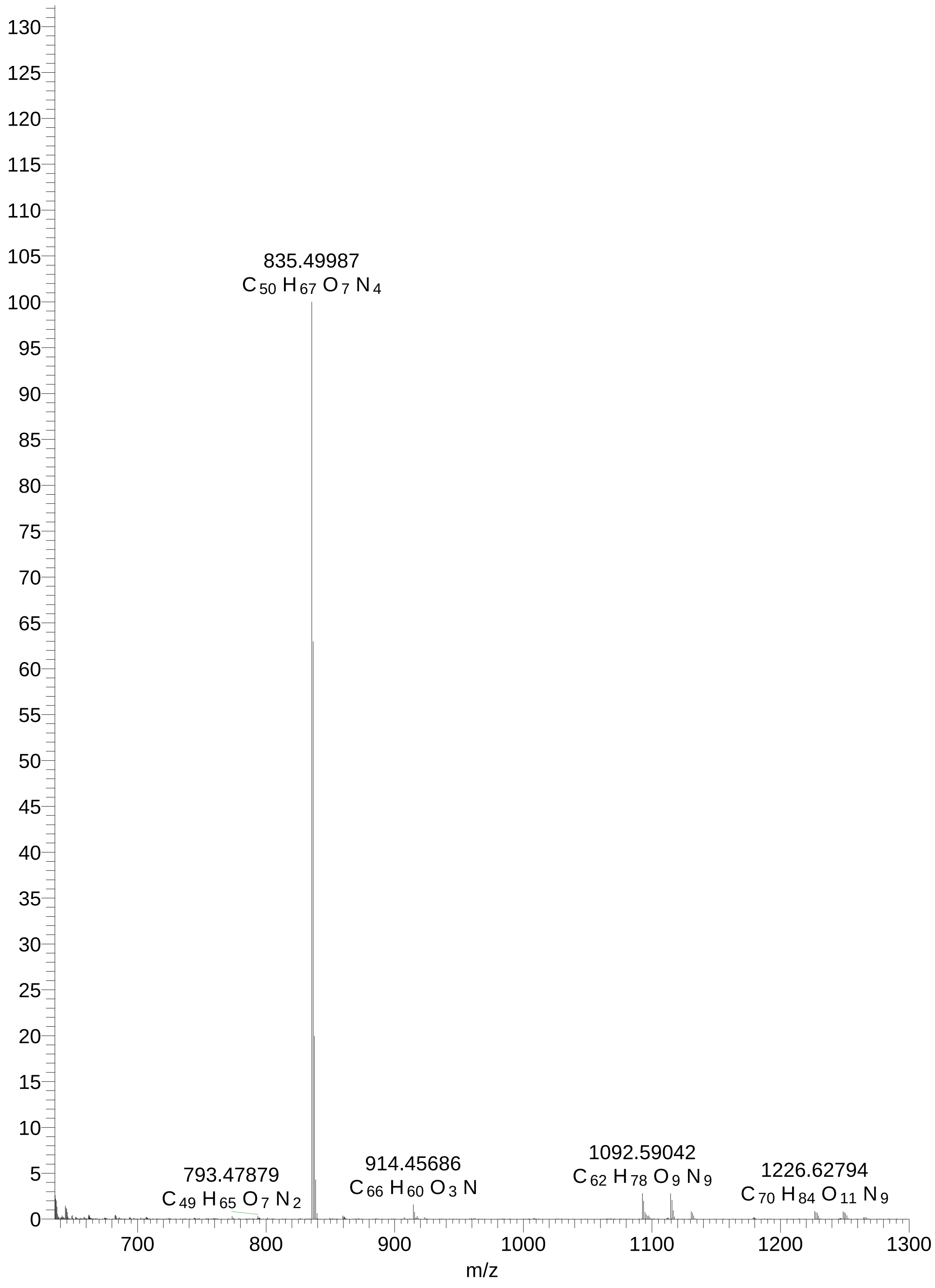

Elemental composition search on mass 1226.62794

m/z= 1221.62794-1231.62794

m/z	Theo. Mass	Delta (ppm)	RDB equiv.	Composition
1226.62794	1226.62848	-0.44	33.5	$C_{70}H_{84}O_{11}N_{9}$

Figure S32. Compound **19**

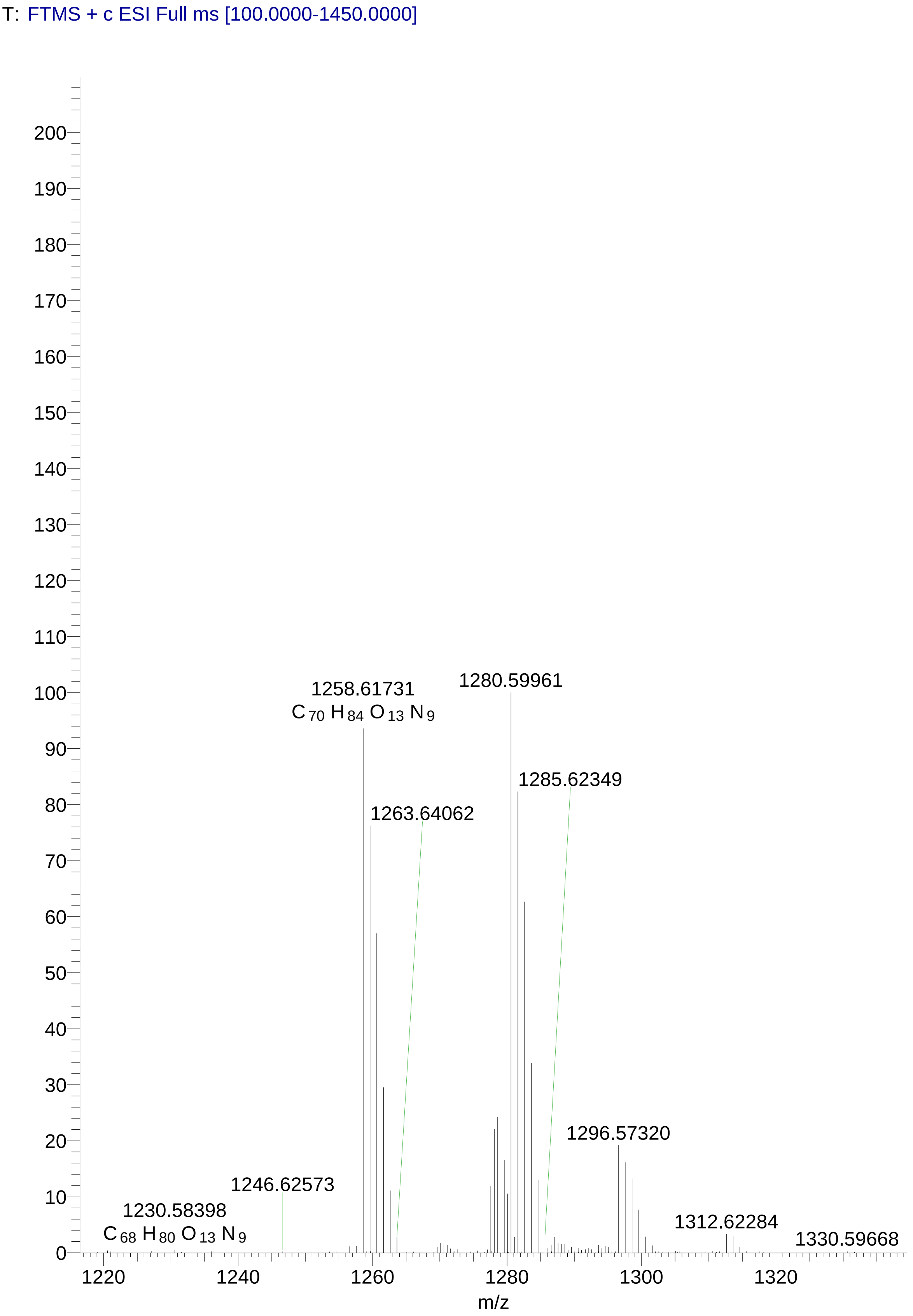

Elemental composition search on mass 1258.61731

m/z= 1253.61731-1263.61731

m/z	Theo. Mass	Delta (ppm)	RDB equiv.	Composition
1258.61731	1258.61831	-0.79	33.5	$C_{70} H_{84} O_{13} N_{9}$

Figure S33. Compound **20**

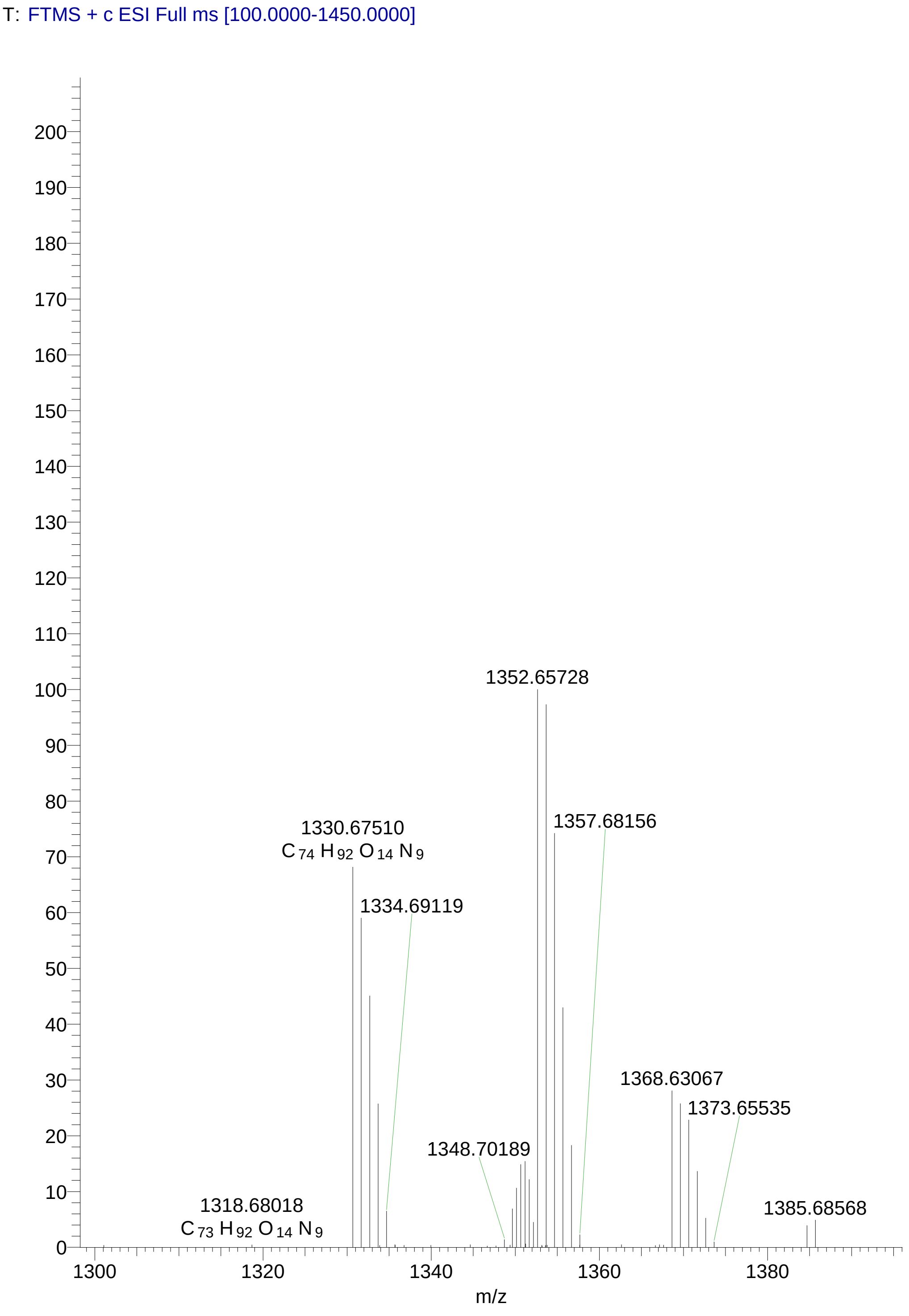

Elemental composition search on mass 1330.67510

m/z= 1325.67510-1335.67510

m/z	Theo. Mass	Delta (ppm)	RDB equiv.	Composition
1330.67510	1330.67582	-0.54	33.5	$C_{74} H_{92} O_{14} N_9$

Figure S34. Compound **21**

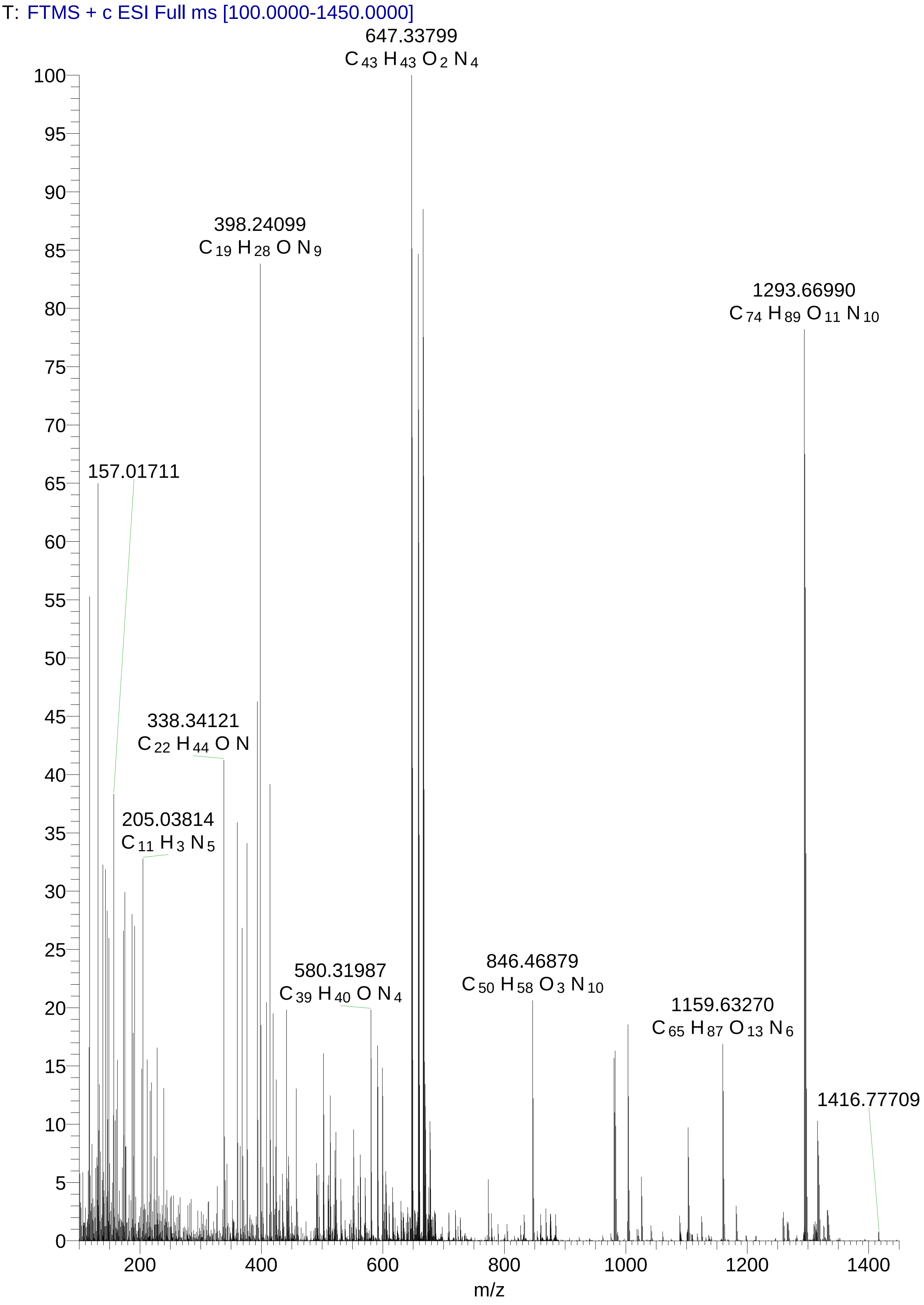

Elemental composition search on mass 1293.66990

m/z= 1288.66990-1298.66990

m/z	Theo. Mass	Delta (ppm)	RDB equiv.	Composition
1293.66990	1293.67068	-0.60	35.5	$C_{74}H_{89}O_{11}N_{10}$

Figure S35. Compound **27**

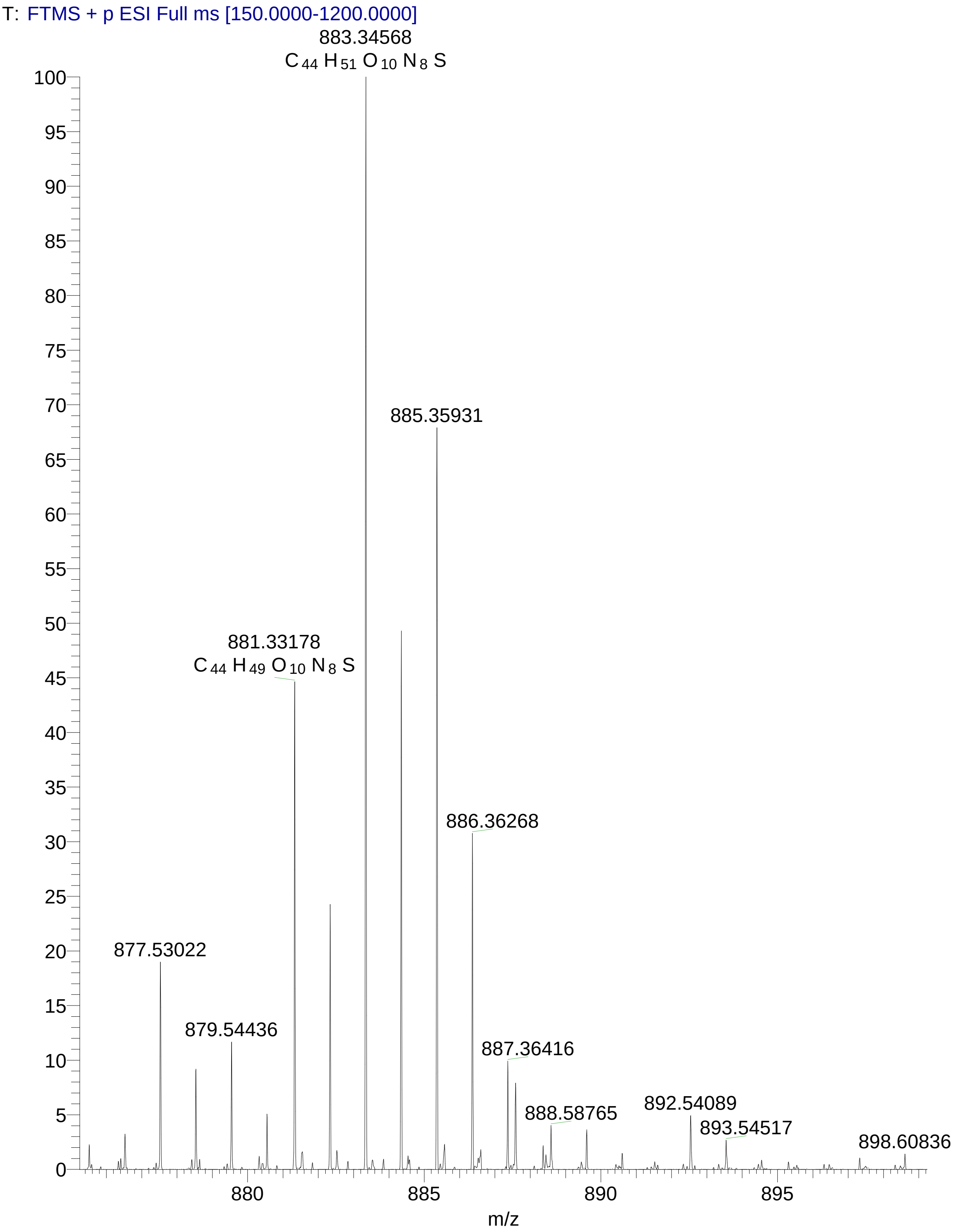

Elemental composition search on mass 883.34568

m/z= 878.34568-888.34568

m/z	Theo. Mass	Delta (ppm)	RDB equiv.	Composition
883.3457	883.3443	1.52	23.5	$C_{44}H_{51}O_{10}N_{8}S$

Figure S36. Compound **28**

T: FTMS + c ESI Full ms [100.0000-1450.0000]

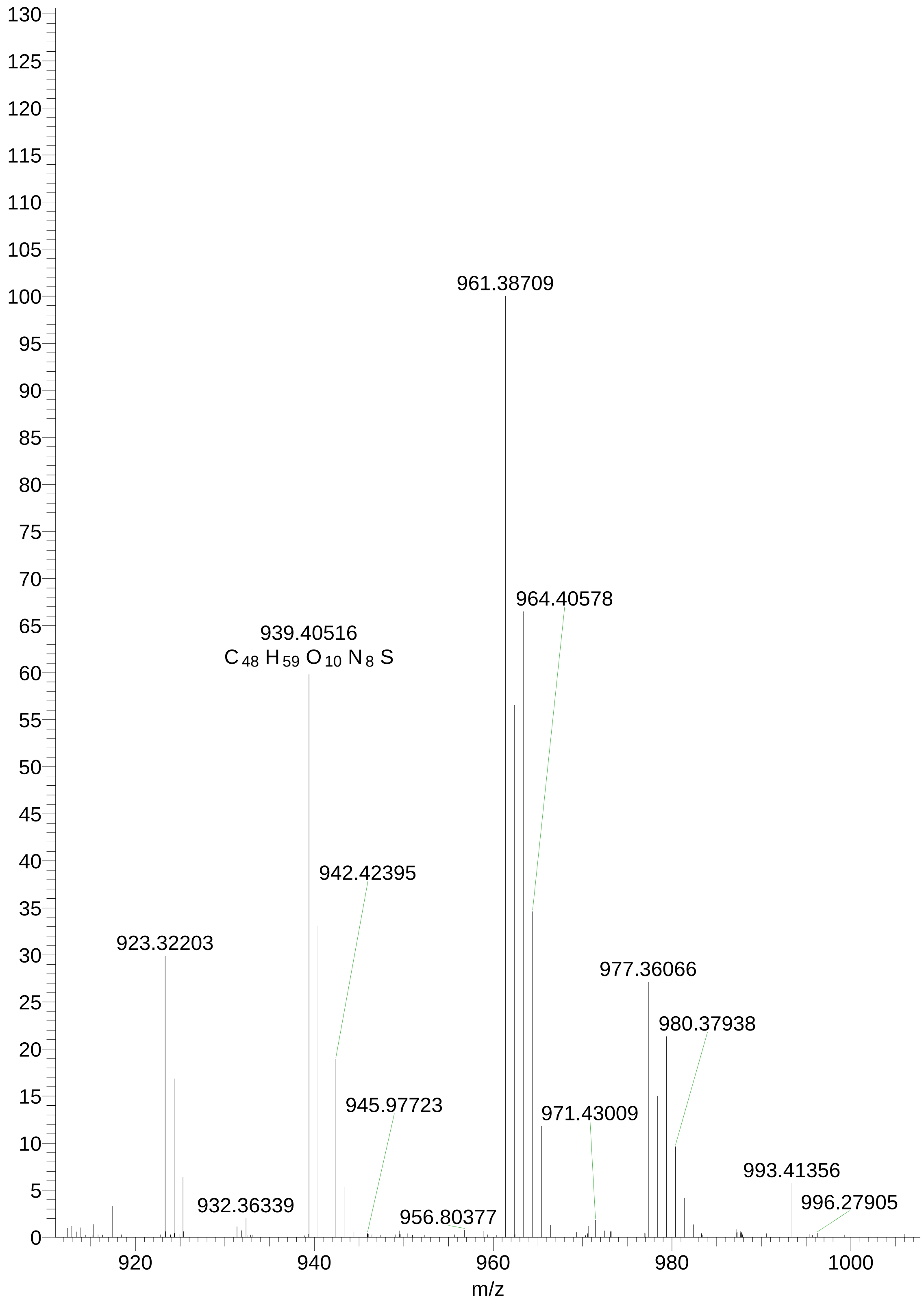

Elemental composition search on mass 939.40516

m/z= 934.40516-944.40516

m/z	Theo. Mass	Delta (ppm)	RDB equiv.	Composition
939.40516	939.40694	-1.89	23.5	$C_{48}H_{59}O_{10}N_8S$

Figure S37. Compound **29**

T: FTMS + c ESI Full ms [100.0000-1450.0000]

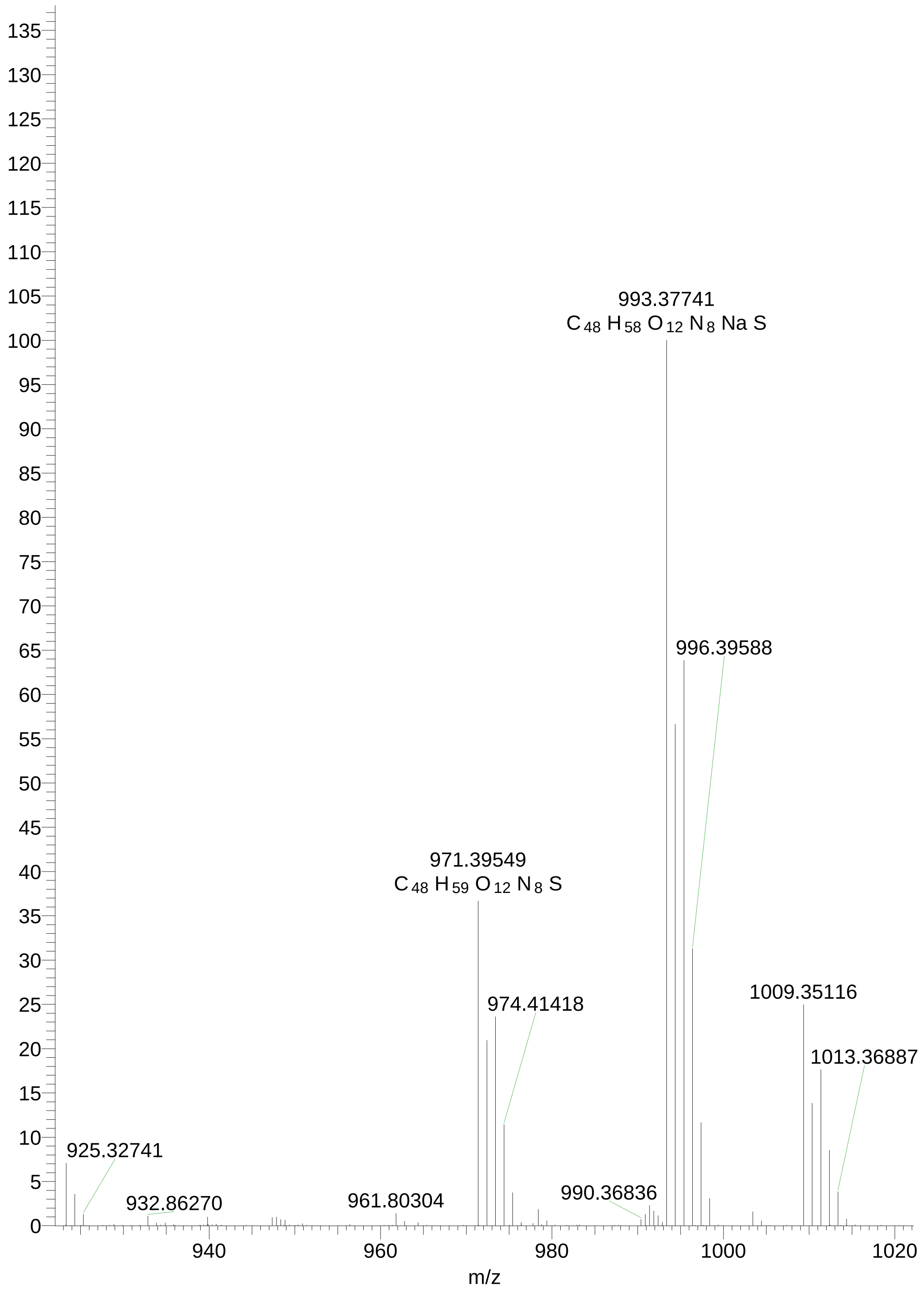

Elemental composition search on mass 971.39549

m/z= 966.39549-976.39549

m/z	Theo. Mass	Delta (ppm)	RDB equiv.	Composition
971.39549	971.39677	-1.31	23.5	$C_{48}H_{59}O_{12}N_{8}S$

Figure S38. Compound **30**

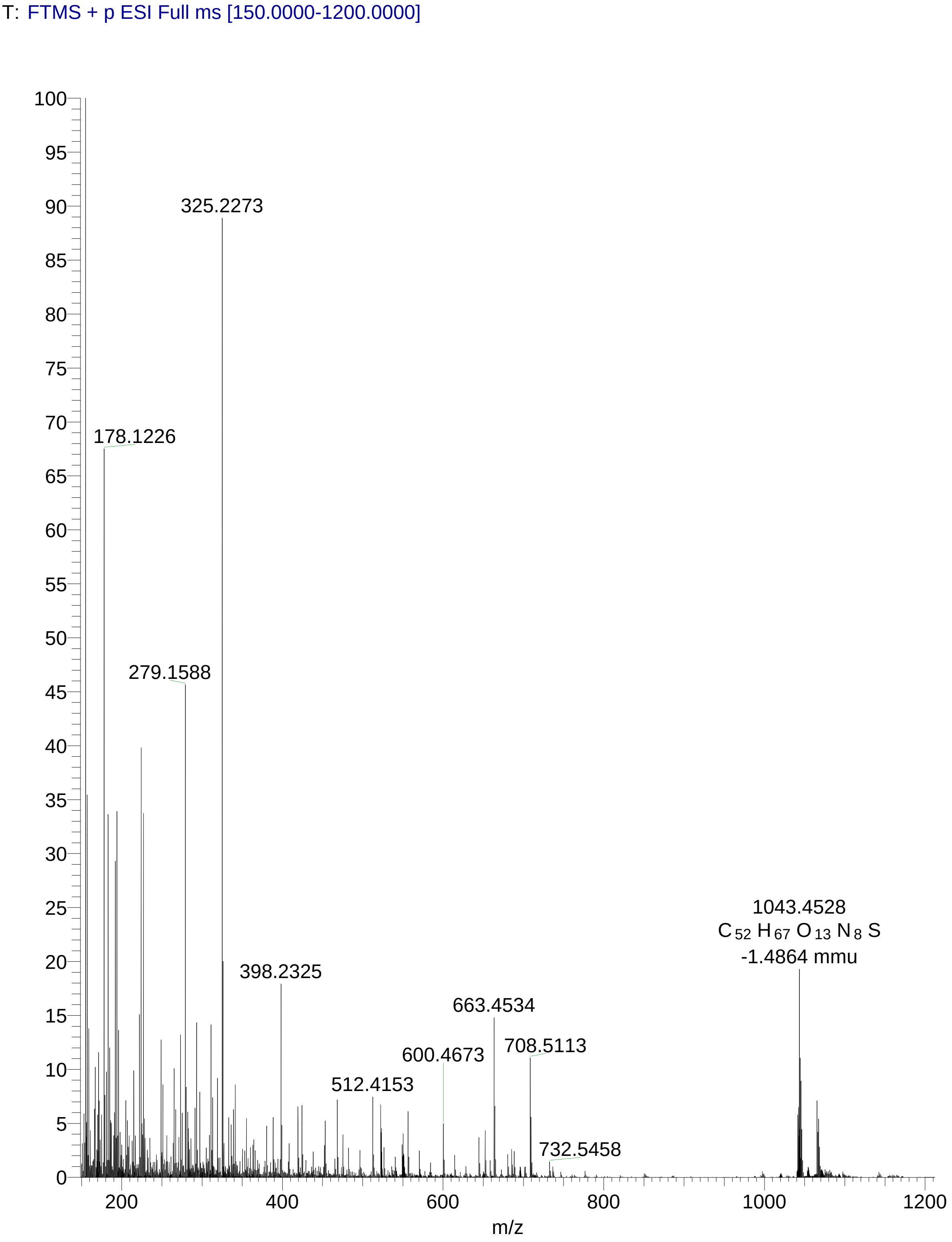

Elemental composition search on mass 1043.45279

m/z= 1038.45279-1048.45279

m/z	Theo. Mass	Delta (ppm)	RDB equiv.	Composition
1043.4528	1043.4543	-1.43	23.5	$C_{52} H_{67} O_{13} N_{8} S$

Figure S39. Compound **31**

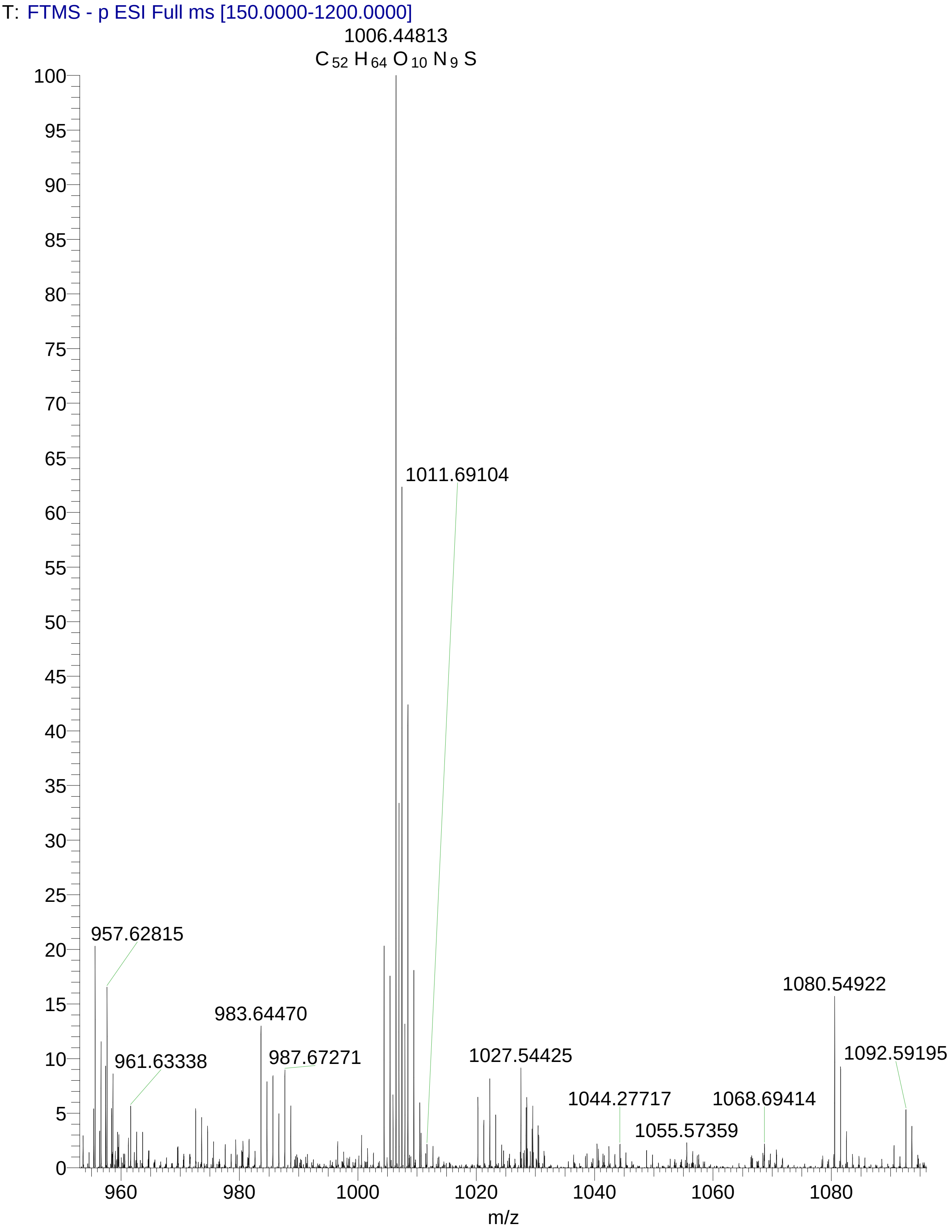

Elemental composition search on mass 1006.44813

m/z= 1001.44813-1011.44813

m/z	Theo. Mass	Delta (ppm)	RDB equiv.	Composition
1006.4481	1006.4491	-1.00	25.5	$C_{52} H_{64} O_{10} N_{9} S$
